# World Heart Federation Roadmap for Secondary Prevention of Cardiovascular Disease: 2023 Update

**DOI:** 10.5334/gh.1278

**Published:** 2024-01-22

**Authors:** Liliana Laranjo, Fernando Lanas, Marie Chan Sun, Deborah Anne Chen, Lisa Hynes, Tasnim F. Imran, Dhruv S. Kazi, Andre Pascal Kengne, Maki Komiyama, Masanari Kuwabara, Jeremy Lim, Pablo Perel, Daniel José Piñeiro, Carlos I. Ponte-Negretti, Tania Séverin, David R. Thompson, Lale Tokgözoğlu, Lijing L. Yan, Clara K. Chow

**Affiliations:** 1 Westmead Applied Research Centre, University of Sydney, Sydney, Australia; 2 Universidad de La Frontera, Chile; 3 Department of Medicine, University of Mauritius, Réduit, Mauritius; 4 The Heart Foundation of Jamaica, Kingston, Jamaica; 5 Croí, the West of Ireland Cardiac & Stroke Foundation, Galway, Ireland; 6 Department of Medicine, Division of Cardiology, Warren Alpert Medical School of Brown University, Providence VA Medical Center, Lifespan Cardiovascular Institute, Providence, US; 7 Department of Medicine (Cardiology), Beth Israel Deaconess Medical Center and Harvard Medical School, Boston, US; 8 Non-Communicable Diseases Research Unit, South African Medical Research Council, Cape Town, South Africa; 9 Clinical Research Institute, National Hospital Organization Kyoto Medical Center, Kyoto, Japan; 10 Department of Cardiology, Toranomon Hospital, Tokyo, Japan; 11 Global Health Dpt, National University of Singapore Saw Swee Hock School of Public Health, Singapore; 12 Non Communicable Disease Epidemiology, London School of Hygiene & Tropical Medicine and World Heart Federation, London, UK; 13 Universidad de Buenos Aires (UBA), Buenos Aires, Argentina; 14 Cardiometabolic Prevention Unit, Instituto Médico La Floresta, Caracas, Venezuela; 15 World Heart Federation, Switzerland; 16 School of Nursing and Midwifery, Queen’s University Belfast, United Kingdom; 17 European Association of Preventive Cardiology, Sophia Antipolis, UK; 18 Department of Cardiology, Hacettepe University, Ankara, Turkey; 19 Global Health Research Center, Duke Kunshan University, China; 20 Faculty of Medicina and Health, Westmead Applied Research Centre, University of Sydney, Australia

**Keywords:** CVD, secondary prevention, fixed combination therapy, polypill, cardiac rehabilitation, influenza vaccination

## Abstract

**Background:**

Secondary prevention lifestyle and pharmacological treatment of atherosclerotic cardiovascular disease (ASCVD) reduce a high proportion of recurrent events and mortality. However, significant gaps exist between guideline recommendations and usual clinical practice.

**Objectives:**

Describe the state of the art, the roadblocks, and successful strategies to overcome them in ASCVD secondary prevention management.

**Methods:**

A writing group reviewed guidelines and research papers and received inputs from an international committee composed of cardiovascular prevention and health systems experts about the article’s structure, content, and draft. Finally, an external expert group reviewed the paper.

**Results:**

Smoking cessation, physical activity, diet and weight management, antiplatelets, statins, beta-blockers, renin-angiotensin-aldosterone system inhibitors, and cardiac rehabilitation reduce events and mortality. Potential roadblocks may occur at the individual, healthcare provider, and health system levels and include lack of access to healthcare and medicines, clinical inertia, lack of primary care infrastructure or built environments that support preventive cardiovascular health behaviours. Possible solutions include improving health literacy, self-management strategies, national policies to improve lifestyle and access to secondary prevention medication (including fix-dose combination therapy), implementing rehabilitation programs, and incorporating digital health interventions. Digital tools are being examined in a range of settings from enhancing self-management, risk factor control, and cardiac rehab.

**Conclusions:**

Effective strategies for secondary prevention management exist, but there are barriers to their implementation. WHF roadmaps can facilitate the development of a strategic plan to identify and implement local and national level approaches for improving secondary prevention.

## A. Introduction

The World Heart Federation (WHF) Roadmaps aim to improve the implementation of evidence-based interventions to reduce cardiovascular disease (CVD) burden and assist with prioritization. They serve as models that can be locally customized or modified and incorporated into national non-communicable disease (NCD) action plans. The WHF Roadmap series covers a large range of cardiovascular conditions, risk factors, and prevention and management strategies [1,2,3,4,5,6,7,8,9,10], which have been developed by the relevant team of WHF experts from around the world. In line with the World Heart Vision 2030: Driving Policy Change, the WHF has taken the initiative to update its various Roadmaps with the goal of reducing cardiovascular mortality and incidence by 30% by 2030 [11].

CVD is currently the world’s main killer, with 20.5 million people dying each year from CVDs, close to one third of all deaths worldwide [12]. Most of the CVD burden is due to atherosclerotic cardiovascular disease (ASCVD), as well as other important CVD manifestations such as heart failure and atrial fibrillation [13]. ASCVDs are the major causes of premature death, disability, and healthcare expenditure globally [14,15,16,17]. ASCVD includes coronary artery disease, cerebrovascular disease, peripheral artery disease, and atherosclerotic aortic disease. More than four out of five CVD deaths are due to heart attacks and strokes, and one third of these deaths occur prematurely in people under 70 years of age [14]. In addition, while CVD accounts for 23% of deaths across high-income countries, it causes more than 42% of deaths in low- and middle-income countries (LMIC) [18, 19].

### WHF Roadmap for Secondary Prevention of CVD

The goal of the present update of the WHF Roadmap for Secondary Prevention of CVD (first published in 2015) [1] is to drive policy change for the reduction of cardiovascular morbidity and mortality in people with ASCVD, through a conceptual framework for the development of ‘sustainable’ and ‘scalable’ national policies and health systems approaches [20]. It also aims to incorporate the substantial new literature and guidelines since 2015, including the 2021 European Society of Cardiology (ESC) Guidelines on Cardiovascular Disease Prevention in Clinical Practice [21], the inclusion of single-pill combinations for hypertension on the World Health Organization Essential Medicines’ list in 2019, of polypills in 2023 [22] and various advances in digital health [10, 23,24,25].

The aims of this Roadmap update are:

1. to describe new and existing strategies for secondary CVD prevention;
2. to identify the roadblocks to evidence-based and high-quality cardiovascular care for the prevention of CVD; and
3. to provide healthcare professionals and policymakers across the world with practical tools to improve cardiovascular healthcare, with consideration for the different contexts patients and health providers live in.

The development of this Roadmap followed an extensive literature review, with input from subject matter experts and researchers with broad representation from both within and external to the WHF. In addition, an online WHF member consultation survey was conducted in November and December 2022, using snowball sampling to include regional members as well as national representatives. The questionnaire was composed of three different sections: 1) sociodemographic questions about the survey respondents; 2) local availability, affordability, and acceptability of specific secondary prevention medications and lifestyle interventions, and existing secondary prevention policies; and 3) perspectives on specific roadblocks and solutions for secondary prevention implementation (identified in the previous version of this Roadmap). In total, 268 responses from 60 countries were collected (see respondent characteristics in Supplement 1).

There is substantial evidence to guide how ASCVD can be prevented and treated and a strong evidence base, including cost-effectiveness data, to support a focus on secondary prevention implementation to reduce CVD burden [21, 26,27,28]. This is also described in multiple international guidelines [21, 29]. In this Roadmap, **secondary CVD prevention** refers to preventing heart attack and stroke through drug therapy and counseling for high-risk individuals—such as those with previous events or known atherosclerotic CVD [1]. Recent scientific discussions also consider secondary prevention for patients with subclinical atherosclerotic disease (i.e., atherosclerotic disease diagnosed through imaging [30, 31]). However, people who have already experienced a major cardiovascular event are at the highest risk of new episodes [21, 32] and they are easy to identify.

Appropriate secondary prevention medication (antiplatelet treatments [aspirin with or without P2Y12 receptor blockers], lipid lowering [statins with or without ezetimibe and other types of lipid lowering medication, e.g., PCSK9 monoclonal antibodies], angiotensin converting enzyme [ACE] inhibitors or angiotensin receptor blockers [ARB] and beta blockers), cardiac rehabilitation, and lifestyle modification (smoking cessation, physical activity, and healthy diet) can significantly reduce the incidence of repeat cardiovascular (CV) events and death (**Box 1**) [21, 33,34,35]. In recent years a range of novel medical strategies have been examined with respect to CVD secondary prevention including anticoagulants [36, 37], novel lipid modifying agents (ezetimibe, PCSK9 monoclonal antibodies, bempedoic acid and inclisiran), SGLT-2 inhibitors and GLP-1 agonists [38, 39]) and anti-inflammatory medicines (e.g., colchicine). A large body of evidence demonstrates chronic subclinical inflammation is a clear contributor to the pathogenesis of atherosclerosis [40,41,42,43,44,45]. In addition, the benefit of influenza vaccination has been demonstrated to reduce the cardiovascular events as a secondary prevention measure in recent randomized clinical trials [46,47,48]. The link between influenza season and increased CVD events has has been observed since the early 1900s. A self-controlled case-series study design describe a sixfold increased risk of myocardial infarction in the seven days after influenza infection [49]. The relationship between respiratory infections and CVD has gained more recognition since the emergence of SARS-CoV-2. Infections such as influenza, pneumococcal pneumonia, and COVID-19 are a contributing factor to major cardiovascular events and all-cause death [29, 50, 51].

**Box 1 Summary of guideline-recommended lifestyle and medication therapy for secondary CVD prevention**

**Medication**

Several medications are first-line therapies in ASCVD, having been shown to reduce the incidence of repeat cardiovascular (CV) events and death [21, 33, 34]:

- Antiplatelets: Aspirin is a foundation treatment for post-acute coronary syndrome and for long-term secondary prevention and supported by large-scale trial meta-analyses [68]. Dual antiplatelet therapy (DAPT), including aspirin and a P2Y_12_ inhibitor, is indicated post ACS, with its duration depending on the individual patient’s risk factors and the type of stent used during percutaneous coronary intervention (PCI). Current guidelines recommend treatment with DAPT for 6 to 12 months after PCI with a drug-eluting stent [69]. However, studies using older stents show that extending DAPT by >12 months after PCI lowers the risk of myocardial infarction (MI) but increases the risk of major bleeding [70]. Studies with second-generation drug-eluting stents suggest shorter-duration DAPT may offer a more favourable benefit/risk ratio in patients with a lower risk of ischemic events or higher risks of bleeding [71]. It is important to discuss the optimal duration of DAPT with the patient and relatives through shared decision making, considering the balance between ischemic/thrombotic risk versus the risk of bleeding.
- Lipid-lowering medication: Cholesterol-lowering statins are the mainstay and indicated in all ACS and secondary prevention guidelines [72,73,74,75]. Additionally, nonstatin therapy (ezetimibe, PCSK9 monoclonal antibodies, bempedoic acid and inclisiran) may have a role in contributing to achieve target goals for low-density lipoprotein cholesterol (LDL-C) in high-risk individuals.
- Beta-blockers are a first line cardioprotective agent for individuals the first year after a myocardial infarction [29]. They are particularly important to be continued in patients with reduced left ventricular function [76, 77].
- Blood pressure (BP) medication: Renin-angiotensin-aldosterone system (RAAS) inhibition of angiotensin-converting enzyme (ACE) inhibitors or angiotensin receptor blockers (ARB) is also first-line therapy post ACS [78]. Achieving lowered BP (e.g., systolic BP <120 mmHg compared to SBP <140 mmHg) with a combination of medicines has been shown to additionally reduce the risk of future CV events [79].

***Other medical treatments***

- Anti-inflammatory treatments are an emerging class of therapeutics in CVD secondary prevention. There is some evidence that anti-inflammatory medications including low-cost colchicine reduces cardiovascular events in people with coronary artery disease [40]. Low-dose colchicine was recently approved by the US FDA for reducing inflammation to prevent CVD [80].
- The body of evidence provides strong support for the impact of influenza vaccination in reducing all-cause death, CV death, and other major CV events [81, 82], especially after acute coronary episodes [83]. Because of its benefits in chronic health conditions, many health authorities recommend annual influenza vaccination. Additionally, with more limited evidence, it is recommended the use of COVID-19 and pneumococcal vaccine [29].

**Cardiac rehabilitation**

Cardiac rehabilitation or secondary prevention programs usually describe a multifaceted program of support, exercise and education generally led by health professionals and provided either after an acute cardiovascular event or cardiac procedure. There is substantial evidence that such programs are effective at reducing events and all-cause mortality, being cost-effective [34, 84,85,86,87,88] and as such, cardiac rehabilitation is recommended by guidelines and routinely offered to patients early after an ACS event [87, 89]. Modern and digital ways of delivering cardiac rehabilitation are rapidly evolving to address implementation gaps and low adherence, as discussed later in this Roadmap.

**Lifestyle**

Smoking cessation

Smoking cessation is associated with a reduction of approximately one third in the risk of recurrent CVD in people who stop smoking at diagnosis [90]. Together with behavioural support, smoking cessation pharmacotherapies are the most effective strategy available to achieve smoking cessation, but more effort is needed to increase their use among cardiac patients [91]. Where available, smokers should be referred to a smoking cessation program post-MI. E-cigarettes are not recommended for smoking cessation [92], with evidence indicating they are harmful to health [93] and may contribute to cardiovascular problems [94,95,96].

Physical activity, diet and weight management

People with ASCVD (and without severe heart failure) should do at least 150 minutes a week of moderate-intensity aerobic physical activity or at least 75 minutes a week of vigorous-intensity aerobic physical activity, and muscle-strengthening activities at least twice per week, as well as aiming to reduce sedentary time by engaging in at least light activity through the day [21, 97].

Guidelines and the most recent meta-analysis evidence advise adopting a Mediterranean or similar diet [21, 98]. Such dietary patterns maintain low or healthy quanta of intake with respect to saturated fat, sodium and simple carbohydrates and are rich in whole fruit and vegetables, nuts, legumes and healthy fats (omega-3 fatty acids). Regarding alcohol, there are no safe recommended levels of alcohol consumption, and abstinence is recommended for secondary CVD prevention [99, 100].

Weight management is an important goal of good lifestyle management. Increased abdominal adiposity is associated with increased cardiovascular risk [101]. Lifestyle modifications (e.g., healthful nutrition, routine aerobic and resistance physical activity) can provide modest weight loss and long-term CV benefits, such as improvements in blood pressure and cholesterol [101]. In patients with severe obesity, bariatric surgery has been shown in observational studies to lower the risk of major adverse cardiovascular events and death, but further evidence from randomised controlled trials is needed to confirm these findings [102]. Emerging evidence also suggest GLP-1 receptor agonists for obesity [103].

Still, poor uptake and adherence to secondary prevention guidelines is common. Observational studies, medical record audits and surveys demonstrate that the proportion of individuals who receive and adhere to recommended secondary prevention care, including medicines and lifestyle modification, is low and inequitable [52, 53,54,55,56,57,58,59,60,61], particularly in LMIC [55, 62,63,64]. Control of underlying risk drivers, such as blood pressure, also remains poor worldwide, with known global inequities and worse rates of control in LMIC [65]. In addition, there are concerns that our rapidly changing climate and environment may drive even greater inequity in the receipt of secondary prevention care, which may lead to larger gaps in CVD burden [66, 67].

## B. Starting point: roadblocks to secondary prevention

There are large gaps between guidelines and practice in the prevention and treatment of CVD, particularly in LMICs [104, 105]. Roadblocks to implementation of CVD prevention are contextual, affected by geography, economic status of countries, health system organization, and sociocultural factors. In this section key barriers to secondary CVD prevention implementation are described at the following levels: individual; healthcare providers; and healthcare system and policy [106,107,108]. Additional important roadblocks also exist across multiple levels, such as structural bias leading to ethnicity and gender-related disparities [56].

### 1. Individual-level roadblocks to secondary prevention

At an individual level, non-adherence to lifestyle recommendations, cardiovascular medications and cardiac rehabilitation are key roadblocks to secondary CVD prevention [56], and these are highly influenced by socio-economic factors [109, 110].

Rates of **lifestyle behaviour change** in secondary CVD prevention remain low, with estimates six months post-event showing around half of the individuals persist in smoking, and 60% do not adhere to physical activity guidelines [55, 57, 111, 112]. Unhealthy lifestyles are difficult to change, and when changed, hard to maintain over time, with relapses common, especially in complex and chronic disease conditions requiring multiple behavioural changes, like ASCVD. The main barriers to lifestyle behaviour change include lack of support from family and friends, beliefs about lifestyle, competing demands, and mental health problems like depression and anxiety [113].

Patient **non-adherence to cardiovascular medications** remains a concern, despite growing research and efforts to mitigate this problem [55, 111, 112, 114,115,116,117,118,119], with self-reported non-adherence rates over the past 10 years remaining at approximately 25% for ACE inhibitors, 20% for statins and beta-blockers, and 10% for anti-platelets, with higher rates of non-adherence in LMIC countries [55, 111, 112]. Reasons for low adherence to medications include out-of-pocket costs of medications, forgetfulness, beliefs that medication is unnecessary or that it will cause side effects, inadequate risk perception, complexity of medication regimens with multiple drugs (frequently not properly understood by patients and families), low health literacy, self-efficacy and social support [120,121,122,123].

Individual-level roadblocks to participation in **cardiac rehabilitation** programs are well-documented and include: out-of-pocket costs, language barriers, and difficulty accessing the cardiac rehabilitation centre (e.g., transportation, limited opening hours) [124, 125]. Results from the EUROASPIRE (European Action on Secondary and Primary Prevention by Intervention to Reduce Events) surveys indicate that of all patients who have been advised to attend cardiac rehab, 20–30% attend less than half of the sessions [55, 112].

For influenza vaccination, limitations to uptake are described as arising from complex behavioural attitudes including complacency, inconvenience, lack of confidence and calculation (individual and societal risk-benefit ratio). The lack of recommendation during medical visits also appears to contribute [126].

### 2. The Healthcare-level roadblocks to secondary prevention

#### i. Inequities in access to cardiovascular care and medicines

Access is a main roadblock at the healthcare level, and there remain inequities in access to all aspects of cardiovascular care, including low availability of evidence-based care in LMICs, uneven distribution of health care providers between urban and rural locations [17], and differential access to secondary CVD prevention medicines, with lower availability and affordability in LMICs [18, 127,128,129].

Cardiac rehabilitation also remains underutilized globally, particularly in female and older adult populations [130,131,132,133,134], and studies commonly report less than one-third of patients post myocardial infarction attend a secondary prevention program [135]. In many countries, barriers to cardiac rehab include a lack of capacity (resulting in waiting lists) or affordability issues.

In our WHF survey, respondents also report the low availability and affordability of programs to support physical activity, diet, smoking cessation, and psychosocial wellbeing as roadblocks (see supplementary material). Across regions, the availability and affordability of lifestyle intervention programmes were highest in Western Pacific and lowest in Africa. Our WHF survey also revealed that priority medications (aspirin, beta blockers, ACE inhibitors, statins) were generally available and/or affordable. Previous studies however have found significant proportions of patients are unable to afford the cost of secondary prevention medicines, especially the combined cost of multiple medicines that are indicated in secondary prevention [136].

#### ii. Clinical inertia

At the healthcare-level, there are known barriers in knowledge, attitudes, and behaviour of healthcare providers, including external barriers such as administrative burden and lack of time, leading to clinician inertia [137] and low implementation of secondary prevention guideline-directed care [106, 138]. Research has suggested that clinical inertia, the lack of treatment initiation or intensification for patients not achieving evidence-based therapeutic goals, is an important contributor to poor clinical outcomes. Patient and clinician education, shared decision making and monitoring with electronic decision support systems could decrease clinical inertia. Some studies have examined GP attitudes identifying themes such as optimism that results will improve and caution relating to potential for adverse effects, as reasons for clinical inertia [139, 140].

#### iii. Limited availability of local evidence-based guidelines for CVD prevention

Secondary prevention implementation has been shown to be worse in low-income countries (LICs), and inversely correlated with country gross national incomes [141], highlighting the importance of localized guidelines (i.e., adapting international guidelines to local settings partnering with local key opinion leaders in implementation, based on acceptable minimum standards) [142, 143]. In our survey, 63% of respondents reported that localized guidelines were in place in their country. Across regions, such localized guidelines were most frequent in Western Pacific (95%), followed by Europe (88%) and South-East Asia (75%), the Americas (57%) and Africa (48%). There was a strong gradient across income groups: HICs: 90%; LICs: 44% (see also supplementary material).

### 3. Policy-level roadblocks to secondary prevention

#### i. Lack of investment in primary care

Primary health care is the most inclusive, equitable, cost-effective, and efficient approach to enhance people’s physical and mental health, as well as social well-being, and is key in secondary CVD prevention [144, 145]. Regular access to primary care is associated with better secondary prevention, including adherence to medication [58]. The COVID-19 pandemic has had a significant negative impact on primary healthcare around the globe and there have been recent calls to action for Governments to further invest in primary care [146, 147].

#### ii. Lack of supportive built-up urban environments

On the community level, rapid urbanization leading to environments with low walkability, increased urban air pollution, and not being supportive of physical activity and healthy eating are known barriers to healthy lifestyles [148]. The rising number of international fast-food outlets in both HIC and LMIC, as well as the unplanned and poorly managed urbanization, are not conducive to healthy lifestyles and are resulting in increased CVD, obesity, and other NCDs [149].

### 4. Structural bias

Structural bias refers to bias that is embedded within the structures and systems of society, such as institutions, policies, and practices, stemming from historical, cultural, and societal norms and values that have been established over time. Structural bias can perpetuate the effects of stereotypes and discrimination, leading to unequal opportunities and outcomes for individuals and groups, minorities. There is growing evidence of the role of structural racism and structural gender bias in health disparities [150, 151]. In secondary CVD prevention, there are well-documented ethnicity and gender disparities [152], and sparse evidence for LGBTQI+ (lesbian, gay, bisexual, transgender, queer, and intersex) people [153,154,155,156].

People of different ethnicities—including Black, Hispanic, American Indian, Asian, and others—experience varying degrees of social disadvantage that puts these groups at increased risk of CVD and poor disease outcomes, including mortality [157]. In the United States, African-American patients are less likely to receive secondary prevention ASCVD treatment [152] and experience the highest mortality rates attributable to CVD and stroke, being more than twice as likely to die of CVD [158]. In the United Kingdom, ethnic disparities in access to cardiovascular care have also been well-documented [159,160,161,162].

Studies in both HIC and LMICs have shown that women with established CVD were significantly less likely than men to be referred to cardiac rehabilitation and less likely to receive BP-lowering medications, lipid-lowering medications, antiplatelets, or any CVD prevention medication [61, 152, 163,164,165]. Not only are women less likely to be referred to cardiac rehab, but they are also more likely to drop out [166], due to multiple factors, including pressure as the primary caretaker of the family, and the lack of financial resources and social support [167], and lack of program tailoring to their specific needs [168, 169].

## C. Implementation strategies for secondary prevention

Addressing the identified roadblocks for secondary CVD prevention is a global priority. The following sections describe key interventions and strategies at the individual level, healthcare level, and policy level to implement guideline-recommended secondary CVD prevention and improve outcomes.

### 1. Individual-level implementation strategies for secondary prevention

#### i. Health literacy interventions

Health literacy is the degree to which individuals are able to access and process basic health information and services and thereby participate in health-related decisions [170]. Limited health literacy prevents people from developing the knowledge, skills, and confidence necessary to engage in their care. Poor health literacy is prevalent in patients with CVD and is associated with increased mortality and morbidity [171, 172]. A 2018 Scientific Statement from the American Heart Association highlights the need to develop healthcare systems that are accessible and for which health literacy is not an obstacle [122], with easily understandable health information materials, clear clinician communication, and improved access to language-appropriate services for culturally and linguistically diverse populations.

Health literacy interventions are key to improve health outcomes [173, 174]. Effective health literacy interventions include the ‘teach-back’ method (checking understanding by asking patients to state in their own words what they need to know or do about their health), methods to improve self-management and empowerment (e.g., encouraging questions; making action plans; helping patients remember how and when to take their medicine), and providing easy-to-understand education materials [175]. Patient education tools and decision aids are key in supporting shared decision making, particularly for complex preference-sensitive decisions, and have been shown to improve treatment adherence and health outcomes across multiple chronic conditions [122, 176,177,178], with standard resources available to assess and ensure their quality [179, 180].

The literature on health literacy interventions for secondary ASCVD prevention is sparse. A 2022 scoping review focusing on secondary prevention of coronary artery disease found that some effective interventions focused on building social support for health and using the ‘teach-back’ method to provide patient education [181], while highlighting a paucity of studies evaluating individual-level health literacy interventions to empower patients [181]. Future research should address this gap and focus on robust evaluations of health literacy interventions for secondary ASCVD prevention.

#### ii. Supporting self-management (lifestyle behaviour change and medication adherence)

Self-management interventions aim to equip patients with skills to actively participate and take responsibility in the management of their chronic condition in order to function optimally [182]. Self-management interventions usually support with knowledge acquisition and a combination of the following: stimulation of independent sign/symptom monitoring, medication management, enhancing problem-solving and decision-making skills for medical treatment management, and changing their physical activity, dietary, and/or smoking behavior [182]. Psychotherapy and cognitive-behavioural interventions focusing on stress management can also be considered in addition to self-management support in ASCVD patients to improve CVD outcomes [19, 21, 183].

Self-management interventions for CVD prevention have different degrees of effectiveness depending on their characteristics. For example, static educational interventions like information booklets seem to be less effective in promoting behaviour change and medication adherence than interactive support with multiple contacts, such as via telephone calls and home visits [115, 184, 185]. Other facilitators of treatment adherence include educational involving both patients and caregivers, reasonably achievable treatment goals, and solutions tailored to addressing a patient’s specific adherence barriers [56, 186]. Interventions applying principles of behavioural economics, such as nudge theory, are also showing promise in supporting behaviour change in patients with ASCVD [187,188,189].

Recent clinical and digital health innovations can be useful in facilitating self-management, by supporting lifestyle behaviour change and medication adherence, and will be further discussed in section 4.I. Fixed-dose combination (FDC) therapy for secondary CVD prevention also can improve medication adherence and clinical outcomes (see section below). There is also demonstrated success of FDC improving adherence and clinical outcomes in hypertension treatment [190], leading to the listing of four different anti-hypertensive combinations on the World Health Organization Essential Medicines List [22, 25].

### 2. Healthcare provider and system-level interventions for secondary prevention

#### i. Strategies to improve secondary prevention in primary care and hospitals

Facilitating access to **primary health care** is key to improve health outcomes in secondary ASCVD prevention [144, 145, 191]. Evidence from systematic reviews shows that primary care interventions like regular planned recall of patients for appointments, electronic decision support interventions [192,193,194], structured monitoring of risk factors and prescribing, and patient education can be effective in increasing the proportions of patients within target levels for cholesterol control and blood pressure [195] and in reducing the risk of death [196, 197].

To maximise the impact of secondary prevention interventions, it is also key to optimise the hospital discharge process after an acute cardiovascular event. Implementing protocols and/or decision support interventions that encourage and systematize the prescription of secondary prevention medications, lifestyle recommendations, and referral to cardiac rehabilitation can increase the systematic uptake of secondary prevention. Such post-MI interventions can include utilising post-discharge care coordinators, and leveraging electronic health record systems (e.g., incorporating decision support in electronic health record [EHR] and making cardiac rehabilitation referral the default option, i.e., from opt-in to opt-out option in the automated electronic health record) [198,199,200]. Additionally, offering all patients influenza vaccination after ACS before discharge or during cardiac rehabilitation (if not already vaccinated) [201] can have a beneficial effect. It is also important to further reduce barriers to cardiac rehabilitation, such as by including partners/caregivers to ensure optimal uptake and completion providing, providing different options and flexibility for attending the program (individual or group; location), reducing or eliminating out-of-pocket expenses for patients, and incorporating motivational incentives based on session attendance [87, 124, 125, 168, 169, 202,203,204].

#### ii. Interventions that strengthen secondary prevention services delivery

All health professionals need to be empowered with training, time and resources to provide compassionate and sustained support for behavioural modification, self-management, and medication adherence. In addition, universities and/or professional cardiovascular societies need to ensure the dissemination of evidence-based guidelines, developed by international bodies, for CVD prevention and management and the training of all healthcare providers, including primary healthcare clinicians. Alternate models of care, better integrating community and hospital, involving a multidisciplinary approach, and supporting community health workers and non-health workers, can be considered as strategies to promote secondary prevention. Evidence shows that regardless of the profession of the health worker, patient counselling is an effective strategy to improve medication adherence in secondary CVD prevention, suggesting the use of task-shifting may address barriers such as clinician lack of time and the global shortage of health workers [105].

**Figure 1:**
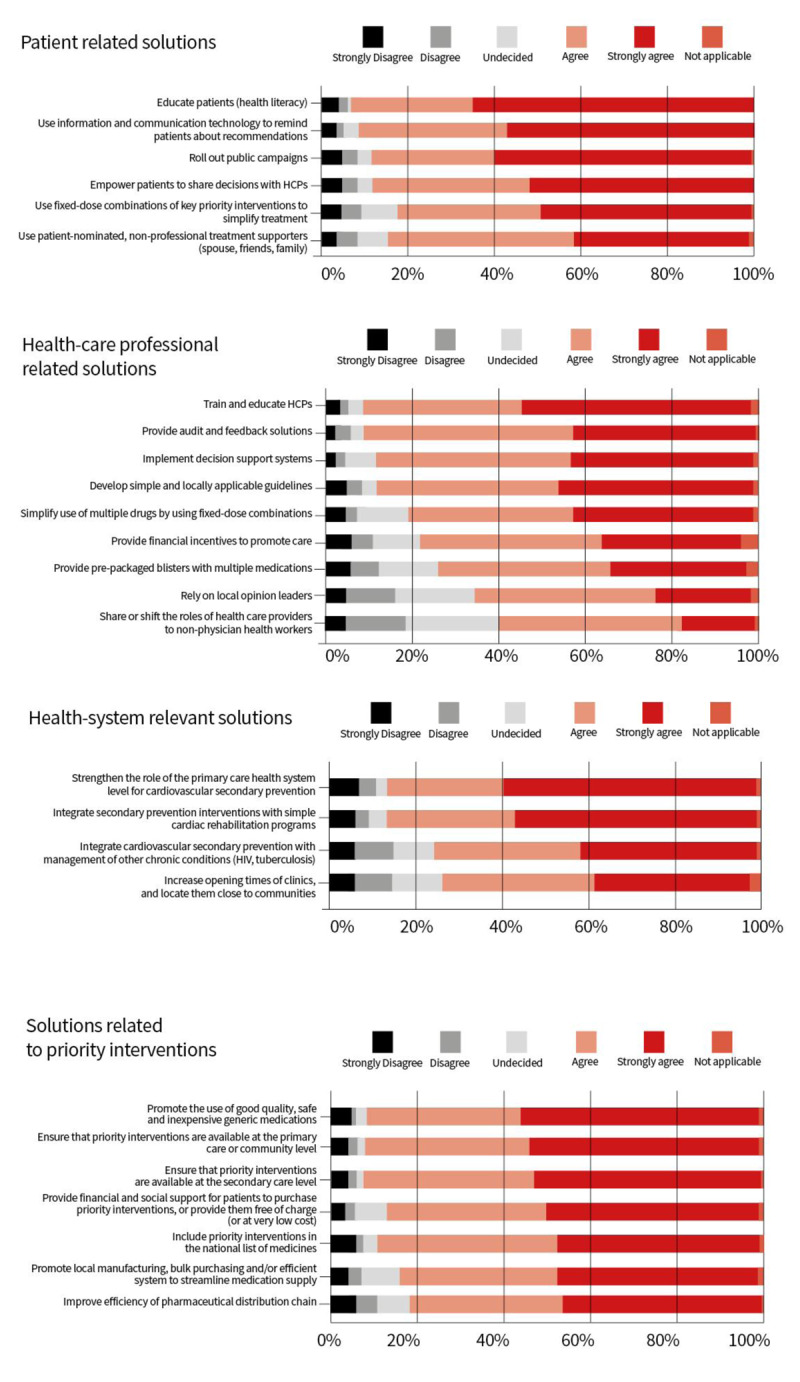
Possible solutions to enable secondary prevention - Responses to the WHF survey on which of the solutions was relevant to their setting.

Respondents to the WHF survey rated the training and education of healthcare professionals, the provision of audit and feedback solutions and the implementation of decision support systems as the most relevant solutions to improve the quality of service delivery. There was a slightly weaker consensus on sharing or shifting the roles of health care providers to non-physician health workers and on relying on opinion leaders (Figure 1).

### 3. Health policy-level interventions for secondary prevention

Health policy interventions are key to support strategies at the individual level and at the healthcare provider and system levels.

#### i. Access to secondary CVD prevention medication

Given the effectiveness of existing medications in secondary prevention, it is critical to guarantee their availability and accessibility. Full prescription coverage and generic medications have been proposed as strategies to improve medication adherence in secondary CVD prevention, but remain under-implemented worldwide [105]. Reducing co-payments has been associated with improvement in adherence [205,206,207,208,209,210,211,212], while evidence of impact on health outcomes is still limited [213, 214].

In that regard, respondents to our WHF survey rated policy solutions that would ‘promote the use of good quality, safe and inexpensive generic medications,’ ‘ensure that priority interventions are available at the primary care or community level,’ and ‘ensure that priority interventions are available at secondary care level’ as particularly relevant (Figure 1).

#### ii. National policies for secondary CVD prevention

One way to rapidly implement secondary prevention strategies at the national level is through national health policy interventions. For example, in Japan, the Stroke and Cardiovascular Disease Control Act was enacted in 2019, and the National Plan for Promotion of Measures Against Cerebrovascular and Cardiovascular basis on the law were released and are being implemented. It is important to address the prevention of cardiovascular disease as a national policy [215].

#### iii. Enabling fiscal policies

**Figure 2:**
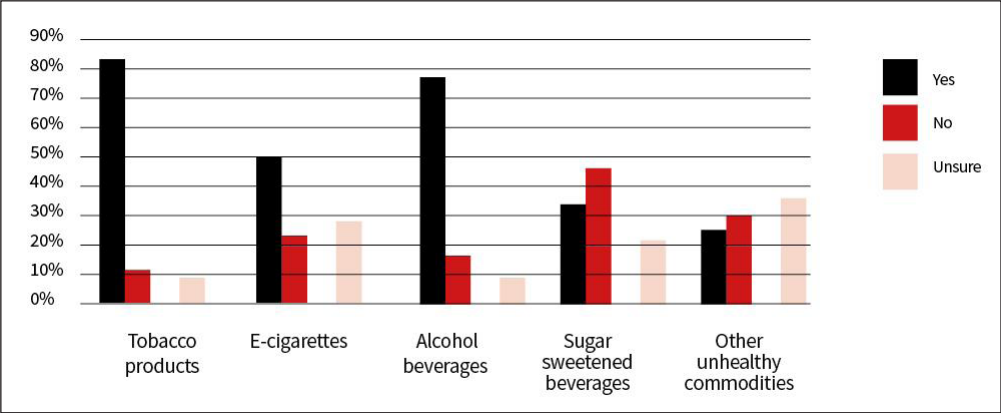
Answers to the question: “In Your country, do you have excise taxes for:…”.

Even though fiscal policies like health taxes (levied on tobacco products, alcoholic and sugar-sweetened beverages, and polluting fuels) could play a role in boosting revenue while also supporting better health, they seem to be under-implemented across the world [216,217,218,219,220,221,222]. This is also reflected in our WHF survey results, which found 81% of respondents were aware of taxes in their region/country for tobacco products, 76% for alcoholic beverages, a figure that reached only 49% for e-cigarettes, 33% for sugar sweetened beverages, and 25% for other unhealthy commodities (Figure 2). A 2022 systematic review and meta-analysis on food taxes and subsidies worldwide found ‘conclusive evidence that fruit and vegetable subsidies to populations with low income were associated with increased sales, whereas food taxes were associated with higher prices and reduced sales’ [218]. There is thus a need to sensitize policymakers on the relevance of fiscal policies to promote healthier purchases [219,220,221,222]. Further research is needed to investigate the implications of food taxes and subsidies, based on country specificities, for food consumption, diet quality, and health outcomes at population level [218].

#### iv. Healthy settings/environments

With the view to reduce CVD risk factors and to reinforce secondary prevention of CVD within the community, there is a need to strengthen community action for public health policies to provide incentives for healthy behaviours with the goal to achieve improved health outcomes. Public health education and regulations are needed to further reduce smoking rates globally. There is also a need to implement health-promoting food courts and supermarkets in close proximity to people and to support urban planning for optimization of the air quality [223] and to create the required environment for the promotion of regular physical activity, as a strategy for secondary prevention of CVD. Another public health strategy is for the health sector to collaborate with governments and businesses to reduce advertising for unhealthy foods and goods, such as sugar-sweetened beverages [224,225,226].

### 4. Recent implementation strategies for secondary prevention

#### i. Digital health for secondary prevention and cardiac rehabilitation

##### a. Digital health to support long-term risk factor management

Digital health is the use of information and communication technologies (e.g., computers, websites) in health and healthcare, and it encompasses the concept of mobile health, which refers more specifically to the use of mobile technologies (e.g., text messaging, smartphone apps, wearables) [227, 228]. Digitally enabled models of care vary with respect to the degree of clinician support and degree of integration into usual clinical care pathways [229]. Digital health interventions can be targeted at the patient, health care provider or the health system more broadly [230].

##### b. Digital health interventions for patients with ASCVD

Recent meta-analyses show promising results of digital health secondary prevention interventions overall, with significant improvements in LDL cholesterol, systolic blood pressure, rehospitalisation, reoccurrence and mortality [229, 231]. Meta-analyses focusing on mobile health for patients after a coronary event also find significant improvements in exercise capacity, physical activity, adherence to medication, and quality of life [232, 233].

Specific landmark randomised controlled trials also demonstrate the effectiveness of mobile phone text-messaging interventions in improving cardiovascular risk factors (e.g., LDL cholesterol, systolic blood pressure, body mass index, lifestyle behaviours) in patients with coronary heart disease [234, 235] living in the community post discharge from hospitals after a CV event. There is also data to support the cost-effectiveness of text message–based secondary prevention of CVD. Another trial tested the use of a wearable fitness tracker in a behavioural economics intervention for patients with ischemic heart disease, showing significant improvements in physical activity [188].

Enablers for digital health intervention use for chronic disease management have been identified in a systematic review focusing on LMIC and include: designs incorporating behavioural science, personalization of content, emphasis on human interactivity, and involvement of end-users in the design and development, taking into account their perspectives and needs (co-design) [236]. Other studies have shown that personalisation of the content and features of mobile health interventions for lifestyle behaviour change are associated with higher effectiveness [237, 238].

Limitations include lack of interoperability which remains a barrier to integration and effective communication and data transfer between these different types of interventions, such as between patient digital interventions and electronic health records, and even between different electronic health records (e.g., between primary and secondary care) [239]. Research gaps include uncertainty about which specific elements of digital health technologies, and in what combination, work for specific populations [240].

One emerging area of research is the use of conversational artificial intelligence (AI) to support and engage patients in lifestyle behaviour change and self-management. Preliminary research suggests conversational AI may be able to support patients by providing CVD prevention information [241] and answering hypertension-related queries [242] but randomised controlled trials are needed to robustly evaluate their efficacy, as well as thorough assessments of their acceptability, trustworthiness and potential safety concerns [243].

##### c. Digital health interventions for healthcare providers in secondary CVD prevention

The most common interventions for healthcare providers in secondary CVD prevention are clinical decision-support systems (CDSSs), usually incorporated within the Electronic Health Record (EHR). CDSSs assist healthcare providers in implementing clinical guidelines at the point of care, using patient data to provide tailored evidence-based recommendations (e.g., reminders for overdue CVD preventive services including screening for CVD risk factors; alerts when indicators are not at goal; recommendations for intensification of treatment) [244, 245]. CDSSs have been shown to be effective in improving clinician practices related to screening and other preventive care services, clinical tests, and treatments in CVD prevention but most studies are in primary prevention and evidence of impact on health outcomes is lacking [246,247,248]. Recent trials are now incorporating principles of behavioural economics into CDSSs [187, 249], with promising effects of electronic nudges delivered to clinicians and patients [189]. Cluster randomised clinical trials in primary care suggest electronic decision support tools have improved cardiovascular risk assessment but not prescription of cardiovascular prevention medicines [250].

The evidence on the effectiveness of mobile health interventions for healthcare providers in secondary CVD prevention is sparser. A cluster randomised controlled trial in rural China showed that a primary care–based mobile health intervention, integrating provider-centered (mobile app) and patient-facing technology (daily voice messages) was effective in reducing blood pressure and improving stroke secondary prevention [251]. In another trial in Tibet, China, and India, a decision-support app for community health workers to promote primary and secondary CVD prevention was found effective at increasing the use of anti-hypertensive medication and aspirin and improving systolic blood pressure [252]. In Australia, a trial assessing electronic decision-support for CVD prevention (primary and secondary), found improvements in risk factor measurements but no significant differences in recommended prescriptions for the high-risk cohort, though a more recent study from the U.S. suggests physician directed reminders improve prescription of statins [250, 253].

##### d. Digital cardiac rehabilitation

Digital health technologies are increasingly being used to deliver cardiac rehabilitation in a more flexible manner [34, 86, 89]. Digital cardiac rehabilitation (also named home-based telerehabilitation, virtual, remote, ehealth or telehealth cardiac rehabilitation) involves the use of information and communication technologies to deliver cardiac rehabilitation remotely, outside of traditional cardiac rehabilitation centres [254, 255]. Studies of digital cardiac rehabilitation have used phone calls, text messaging, the Internet, smartphone apps, virtual reality, and wearable and wireless monitoring devices to deliver patient education, behavioural change support, remote exercise supervision, risk factor management, and psychosocial support [256, 257].

There is accumulating evidence that digital and hybrid cardiac rehabilitation models can have similar safety and efficacy to traditional in-person centre-based cardiac rehabilitation, particularly in low- and moderate-risk patients, and might lead to higher adherence [34, 89, 258,259,260]. However, real-world implementation evaluations of digital cardiac rehabilitation remain sparse [261], though increased implementation occurred during the COVID-19 pandemic [262, 263]. In addition, reimbursement for digital cardiac rehabilitation by government or insurance companies remains scant, raising questions of sustainability and patient access [264].

When deciding which model to recommend, it is important to consider potential predictors of non-participation in digital cardiac rehabilitation, such as higher age, lower digital literacy and lower educational level [265]. Other factors include patient reported barriers to adherence to digital cardiac rehabilitation, like lack of interaction and social support, long duration of education sessions, and technology issues [266]. Ultimately, the choice of digital versus traditional centre-based cardiac rehabilitation can reflect local availability and consider the preference of the individual patient, as part of a shared decision-making process. Further research is needed to understand which technologies work best, for which participants, and in what contexts, as well as their effects on outcomes in diverse populations [89].

#### ii. Fixed-dose combination therapy

**Figure 3:**
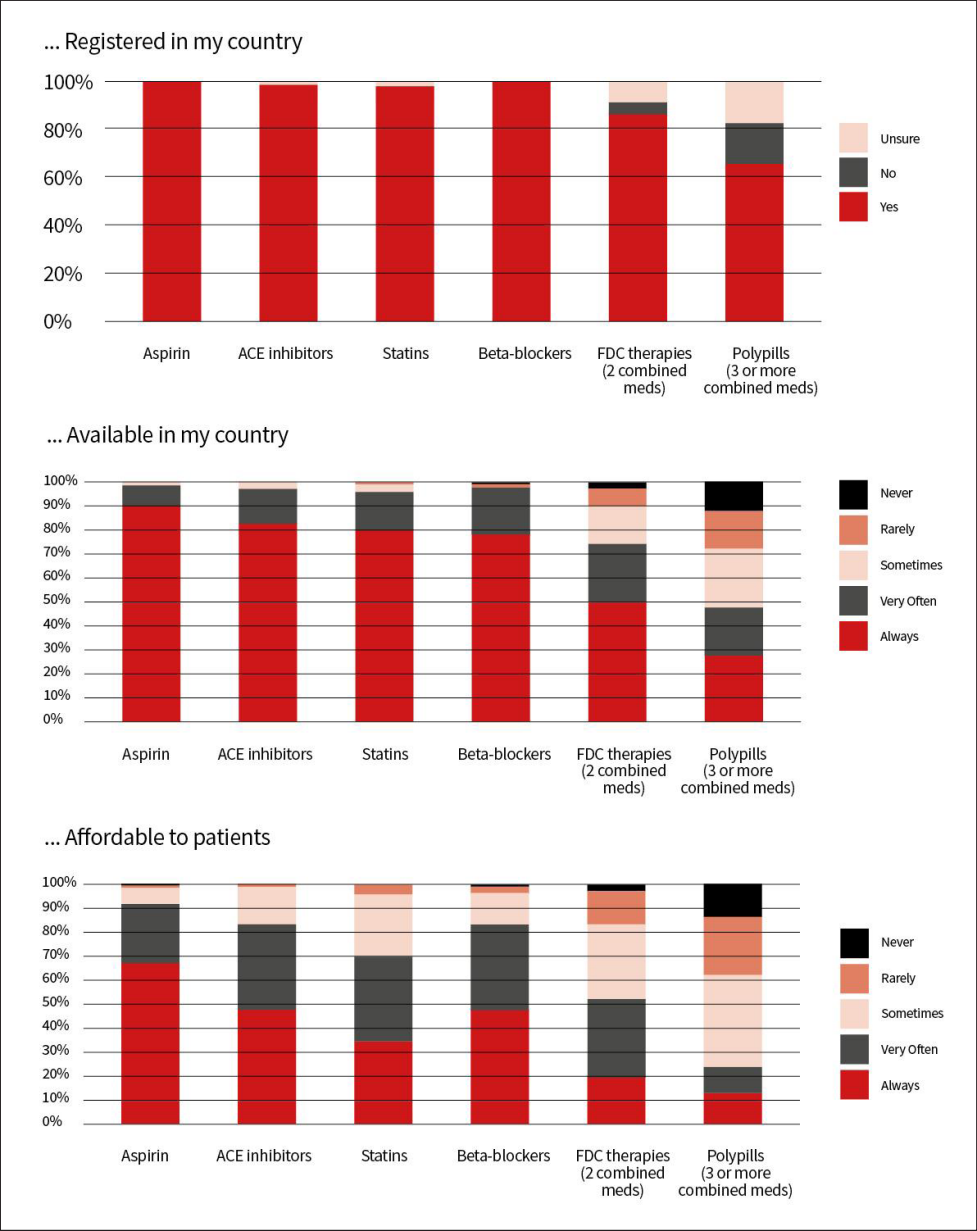
Perceptions with respect to availability and affordability of secondary prevention medicines.

Fixed-dose combination therapies are medications that combine multiple active pharmaceutical ingredients. Fixed-dose combination therapy can simplify a patient’s therapeutic scheme by reducing the number of tablets needed per day, thereby promoting medication adherence [267]. This approach was also successful in hypertension treatment [190], leading to the listing of four different anti-hypertensive combinations on the World Health Organization Essential Medicines List [22, 25]. Randomised controlled trials evaluating the use of fixed-dose combination therapy in secondary CVD prevention have shown improvements in medication adherence and risk factor control [268,269,270,271]. Recently fixed-dose combination therapy for secondary CVD prevention also resulted in lowering risk of major adverse cardiovascular events. The SECURE trial of 2499 patients randomized to the polypill strategy, with a polypill containing aspirin, ramipril, and atorvastatin, or usual care within 6 months after myocardial infarction resulted in a significantly lower risk of major adverse cardiovascular events than usual care (hazard ratio 0.76; 95% CI 0.60 to 0.96; p = 0.02) [272]. Given the accumulated evidence showing the benefits of fixed-dose combination therapy it is important to improve its availability and access worldwide, particularly in LMIC, where the biggest gaps are seen [25, 273]. Survey results for fixed-dose combination (FDC) medicines and the polypill in terms of their registration, availability, and affordability were mixed, with the greatest barriers in LICs (Figure 3). One important milestone for the successful dissemination of fixed-dose therapies in secondary ASCVD prevention is their inclusion in the WHO Model List of Essential Medicines in 2023, after the addition of anti-hypertensive fixed-dose combinations to the List in 2019 [274].

## D. Cost-effectiveness of scaling up secondary prevention

Careful evaluation of the cost-effectiveness of preventative interventions, individually and in combination, can help motivate the investments necessary to scale up effective programs. In general high-quality evaluations will systematically include all costs and benefits of the intervention, including the cost of the intervention (e.g., the cost of the therapy), programmatic costs (e.g., costs of finding eligible patients and initiating the therapy or supporting adherence), downstream savings and health benefits from averted cardiovascular events, and any increased costs related to adverse events [275].

Cost-effectiveness analyses must be contextualized because of differences in disease burden, health care delivery, and costs; so that results may not be directly generalizable among health systems [276]. Each health system must identify its own ‘cost-effectiveness threshold’ in order to determine how much expenditure per incremental life-year—or quality-adjusted life year—would be considered reasonable. Finally, in addition to cost-effectiveness, which is a measure of the efficiency of the intervention, policy-makers must also consider two other related but different issues related to the adoption of cardiovascular prevention strategies: the total effect on population health, including the effect on health equity, and the budget impact (i.e., the effect on total healthcare spending [277]).

Novel, high-cost medications, such as self-injectable monoclonal antibodies against PCSK9 enzyme, may be clinically beneficial [276, 278] but may not meet traditional cost-effectiveness thresholds in many countries without substantial price discounts [279]. On the other hand, analyses have shown that the adoption of generic statin formulations in eligible individuals is likely cost-saving, that is, the therapies more than pay for themselves in the long run due to savings that result from averted events [280, 281]. The economic value of other medical interventions, such as blood pressure medications, varies with the uptake and cost of generic formulations, but blood pressure control in populations at risk of recurrent events, particularly stroke, is typically cost-effective.

The most impactful analyses must also examine novel delivery interventions that may help scale-up prevention efforts, improve adherence, or both. For instance, an evaluation of barbershop-based pharmacist-led intervention to improve blood pressure control in a higher-risk population in the USA suggested that the program was likely to be cost-effective at scale [282]. Another prevention strategy that is gaining traction is the use of a polypill containing a fixed-dose combination of secondary prevention medications, with several analyses suggesting that the adoption of the polypill would be very cost-effective across a wide range of health systems; nevertheless, meaningful health impact would require simultaneous investments in the primary care infrastructure to increase uptake of cardiovascular prevention [283,284,285]. There is also some emerging data on the cost-effectiveness of digital health interventions [286, 287].

## E. Conclusion

In summary, improving secondary prevention is critical to reducing cardiovascular morbidity and mortality. Further comparative effectiveness research on interventions to address disparities in CVD secondary prevention is needed to evaluate potential strategies to close existing gaps [288].

National and local roadblocks and strategies to overcome them should be addressed by Cardiology Societies and Foundations, professional experts, together with consumers and Health Authorities together. The 2015 World Heart Federation Roadmap for Secondary Prevention of Cardiovascular Disease, this new version and the WHF Roundtables can support this process. Critical elements are guidelines adapted to national conditions, available and affordable lifestyle interventions and secondary prevention medication, support to the health care provider team, making adherence to prescription a central part of patient care, increasing evidence of the effectiveness of digital health, and fixed-dose combination therapy.

## F. Case studies

### Data-driven identification of disparities in the secondary prevention of cardiovascular disease in rural South Africa [289]

Secondary prevention is vital for reducing repeated cardiovascular events, and controlling cardiovascular risk factors is critical. This south-African study sought to evaluate disparities in the management of cardiovascular disease in rural communities by determining the frequency of CVD and how it is managed in the rural community of the Agincourt Health and Demographic Surveillance System (HDSS) site in the Mpumalanha province. The CVD population was more likely to be female, older, with a higher BMI, greater waist circumference, and higher total cholesterol.

Self-reported stroke, myocardial infarction, or a diagnosis of angina pectoris using the Rose criteria were used to characterize cardiovascular illness [290].

Out of 5,890 eligible participants, 592 had self-reported CVD. Angina was recorded in 77.0% of the individuals, stroke in 25.2%, and myocardial infarction in 3.7%. In the stroke group, 65.8% were being treated, and in the group with a previous myocardial infarction, 86.4% were under treatment. In total, 24% of patients with CVD were receiving treatment.

Overall, more than 57% of the patients with CVD had only two or fewer risk factors controlled and less than 5% had all five risk factors under control. When the number of risk factors controlled was broken down by sex, 55.8% of the male patients and 34.2% of the female subjects had three or more risk factors under control. In comparison, 13.0% of men and 28.3% of women had only one risk factor under control.

### Outcomes

This study shows that secondary prevention of CVD is currently not optimally managed especially considering the need to control all cardiovascular risk factors. It also sheds light on notable differences in how CVD is managed in rural South Africa. Women and people with lower incomes and educational levels had a reduced likelihood of receiving appropriate management. The treatment of hypertension, a prominent risk factor for CVD, was also shown to have large gaps. The study emphasizes the requirement for focused interventions to enhance CVD management in rural South Africa, especially among vulnerable groups. According to the study, efforts to enhance secondary prevention should concentrate on women, individuals from lower socioeconomic backgrounds, and people who have disabilities.

### Equitable access to medication improves 1-year survival after acute myocardial infarction [291] (Chile)

In an effort to increase equitable access to high-quality medical care for a number of priority health conditions, including acute myocardial infarction, Chile adopted a universal system of health guarantees in 2005, called AUGE.

A practice guideline for the management of MI was created as part of this program with the aim of optimizing diagnosis, treatment, and rehabilitation. One of the guidelines’ recommendations was to initiate secondary preventative medication therapy before hospital discharge (aspirin, beta-blockers and statins, as well as ACE inhibitors or ARBs if relevant).

### Outcomes

A study compared one-year survival of MI patients enrolled in the pre-AUGE period and in the post-AUGE period. Overall one-year case fatality decreased from 9.3% in the pre-AUGE period, to 6.4% in the post-AUGE period. One of the main findings of this study was the significant increase in in-hospital and discharge medication in the post-AUGE period, in particular with regard to statins (40% vs. 89.9%). In addition, in the post-AUGE period, the rates of medication at hospital discharge and after one year of follow-up were highly similar: 90% versus 95% for aspirin, 70% versus 71% for beta-blockers; 89% versus 87% for statins and 72% versus 71% for ACE inhibitors and ARBs. Although causal inference is typically not possible from pre-/post- comparisons, these findings suggest that this intervention is associated with increased uptake of and adherence to cardioprotective drugs, which is expected to result in improved survival. This stability suggests that the early initiation of cardioprotective drugs and adequate access to medication benefits adherence, with a positive outcome on survival.

In conclusion, the implementation of AUGE in Chile has contributed to improved treatment of MI in public hospitals, thus increasing one-year survival. Better access to care, improved care, improved adherence to clinical guidelines by medical professionals and improved patient adherence to medication have all contributed to this positive outcome.

### Implementing a primary care-based integrated mobile health intervention for stroke management in rural China [292]

Stroke is a major public health issue in China, particularly in rural areas where access to treatment is restricted. The SINEMA trial sought to assess the efficacy of a primary care-based integrated mobile health intervention for the secondary prevention of stroke in rural China.

In this programme, 50 villages were randomly assigned (1:1) to either the intervention or control group (usual care). Doctors practicing in villages that belonged to the intervention group received training, carried out monthly follow-up visits supported by an Android-based mobile application, and received performance-based payments. Participants also received automated daily voice messages in addition to monthly medical visits. To assess the effectiveness of this programme, the primary outcome was the 12 months change in systolic blood pressure. Secondary outcomes included diastolic BP, health-related quality of life, level of physical activity, self-reported medication adherence (antiplatelet, statin, and antihypertensive), and performance in ‘timed up and go’ test. Overall, 611 intervention and 615 control patients were monitored for a year across all villages.

After this follow-up period, a significant reduction in systolic BP was observed in the intervention group as compared with the control group, with an adjusted mean difference: –2.8 mmHg. In addition, improvements were observed in 6 out of 7 secondary outcomes (diastolic BP reduction, health-related quality of life, level of physical activity, statin and antihypertensive medications adherence, and performance in ‘timed up and go’ test). Furthermore, reductions in all exploratory outcomes, including stroke recurrence (4.4% versus 9.3%; risk ratio; risk difference, hospitalization, and death), were observed.

The implementation of a robust primary care-based integrated mobile health intervention helped improve stroke management in rural China. When compared to the control group, the intervention group adhered to secondary prevention strategies such as blood pressure control, cholesterol management, and antiplatelet therapy at a much greater rate. Recurrent stroke and all-cause death were also considerably reduced in the intervention group. This programme’s results could guide further initiatives in China and in other low- and middle-income nations dealing with similar stroke management challenges.

### Improving adherence to cardiac rehabilitation [293] (US)

A large body of literature supports the use of CR after an acute MI. Despite these known benefits, only 14% to 30% of eligible patients participate in cardiac rehabilitation, because of difficulty in attending and low referral rates, among others. In addition, participation in CR appears to have declined further during the pandemic [294].

In this programme/study, some enrolees used a mobile technology application to support their participation in cardiac rehabilitation. This application (wellframe.com) featured an interactive daily checklist of written- and video-based educational and support materials as well as twoway messaging between the patients and the cardiac rehabilitation programme staff.

Tailored educational material was provided (i.e., smoking cessation materials for patients who smoke or recently quit smoking; the importance of weight change for overweight or obese patients, etc.).

The staff monitored each patient’s progress on a daily basis through a real-time webbased dashboard. This allowed health care professionals to answer questions; monitor whether the patient had opened the material sent via the Wellframe application; monitor physical activity via the application; and provide support and encouragement.

At the end of the programme, those in the mobile technology cardiac rehabilitation group had attended a higher number of prescribed sessions (mean 28 versus 22), were 1.6 times more likely to complete the programme, and had a slightly greater weight loss (pounds); other outcomes were similar between the groups. In total, the percentage of completion was 68.4% for the standard cardiac rehabilitation group and 80.2% for the mobile technology group, showing that the use of mobile technology can play a positive role in improving adherence to cardiac rehabilitation and number of attended sessions.

### Implementing a polypill strategy in older patients for secondary prevention [272] (Europe)

Even though effective pharmacotherapy for secondary prevention exists, the incidence of recurrent ischemic events remains high. Because of treatment complexity, polypharmacy, treatment of asymptomatic conditions, coexisting illness and age, patients tend to fail to adhere to their medication, and such a lack of adherence to medication tends to lead to poorer outcomes.

Between 2016 and 2019, a randomized controlled trial was conducted in 113 centres across 7 European countries (Spain, Italy, France, Germany, Poland, the Czech Republic and Hungary). It included 2499 patients over age >=75 or >=65 plus one cardiovascular risk factor who had myocardial infarction in the past six months. Patients were randomly assigned to either a polypill strategy (aspirin 100mg, atorvastatin 20 to 40mg, ramipril 2.5, 5, or 10mg) or usual care as per European Society of Cardiology guidelines.

The primary outcome was a composite of cardiovascular death, nonfatal MI, stroke, or urgent revascularization. Treatment adherence at two years and treatment satisfaction were also investigated as secondary outcomes.

Regarding primary outcome, the median follow up was three years. The study found that the risk for primary outcome was significantly lower in the polypill group (9.5%) versus the usual care group (12.7%). Adverse events were similar across groups.

In addition, after two years, self-reported medication use was higher in the polypill group than in the usual-care group (74.1% vs. 63.2%). Similarly, treatment satisfaction was higher in the polypill group (74.4% vs. 67.8% for the usual-care group).

This study highlights the benefits of the polypill strategy for secondary prevention in older patients.

## Additional File

The additional file for this article can be found as follows:

10.5334/gh.1278.s1 Supplementary material. Supplementary summary of WHF member survey.
