# Supplementary material for: World Heart Federation Roadmap for Secondary Prevention of Cardiovascular Disease: 2023 Update
Source: Glob Heart. 2024 Jan 22;19(1):8. doi: 10.5334/gh.1278 (PMC10809857; doi:10.5334/gh.1278)

World Heart Federation Roadmap for Secondary Prevention – WHF member Consultation 2022

Summary of results

Demographics

An online consultation of all WHF Members was conducted in November and December 2022, using snowball sampling to include regional members as well as national representatives. This online survey was composed of three different sections. The first section included demographical questions. The second section included a range of questions on secondary prevention medications and lifestyle interventions as well as secondary prevention policies. The last section focused on the roadblocks and solutions that had been identified in the original WHF Roadmap for secondary prevention. In total, we collected 268 responses from 60 different countries. The highest number of responses were collected in the WHO Region for the Americas (46,7%), followed by the African Region (25,6%). The Western Pacific, South-East Asian and European Regions each accounted for between 10, 4% and 7,2% of responses. Finally, there were only 4 respondents from the Eastern Mediterranean Region (1,5%)[1]. Looking at country income level, 33,83% of responses were from upper-middle income countries (UMIC); 27,1% from lower-middle income countries (LMICs), 24,44% from high-income countries (HICs) and 16,2% from low-income countries (16,2%). Respondents predominantly worked in an urban setting (82,4%); only 6,4% worked in rural locations and 16,1% in semi-urban settings. 33,2% worked in public hospitals, 22% in private hospitals, 13,81% in research organisations, including universities. Other work settings included government organisations and NGOs. 60,3% of the respondents were cardiologists, followed by nurses (8,2%), researchers (5,2%) and physicians (4,5%). Other professions mentioned included paediatricians, pharmacists, physiotherapists, exercise physiologists, among others.

Priority medication, lifestyle and policy interventions

The second section of our online survey focused on the registration, availability, affordability and acceptability of a range of priority secondary prevention medications and lifestyle interventions. It also looked at the availability of specific policy interventions and included a specific question on patient adherence.

Questions on priority secondary prevention medications focused on Aspirin, ACE inhibitors, Statins, Beta-blockers, FDC therapies (2 or more combined medicines for one purpose) and polypills (3 or more combined medicines for different purposes). For each of the four aspects investigated (registration, availability, affordability and acceptability by patients), a gradient can be observed. Aspirin, ACE inhibitors, Statins and beta-blockers are registered almost everywhere. They are “always” available in a strong majority of countries: from 78,7% (statins) to 88,2% (aspirin); Aspirin is the most affordable of these single medications (66,67% always; 26,1% very often). It is followed by ACE inhibitors and beta-blockers (46% always and 39% very often), and then by statins which were reported to be only “sometimes” or “rarely” available by 30% of the respondents. Finally, Aspirin is best accepted by patients (61.9% always and 35.1% very often), followed by ACE inhibitors, beta-blockers and then statins.

[1] Because of the very small number of respondents from the Eastern Mediterranean Region, responses from this region are not discussed in regional trends as they are less representative.

With regard to combined medicines, FDC therapies were reported to be registered by 85,9% of all respondents and polypills by 64,3%. Of note, 17,8% of all respondents were "unsure" whether polypills were registered in their country, a figure which amounted to 68,4% in Western Pacific. FDC therapies were reported to be only "sometimes" or "rarely" available by 14,9% and 5,9% of all respondents, respectively. This figure amounted to 23,3% and 15,9% for polypills, which were also reported to be "never" available by 12,7% of survey respondents. Similarly, FDC therapies were reported to be "sometimes" or "rarely" affordable by 33.9% and 11.8% of respondents; polypills by 36.3% and 25,1% of respondents (13.4% indicated polypills were "never" affordable). Their acceptability by patients was also lower than that of single medications. Across regions, the availability and the affordability of polypills represented a bigger challenge in South-East Asia, Africa and Western Pacific. It also represents the greatest challenge in LICs.

The picture was more contrasted for lifestyle intervention programmes. 48,2% of all respondents reported that programmes related to physical activity were "always" available; a figure that amounted to 39,0% for healthy diet; 37,1% for smoking cessation and 21,5% for psycho-social management. Psycho-social management programmes were reported to be only "rarely" available by 22,4% of respondents. They were also considered to be the least affordable. Across regions, the availability and affordability of lifestyle intervention programmes is highest in Western Pacific, and lowest in Africa.

From the respondents' perspective, lifestyle interventions are also only moderately well-accepted by patients. There was also a strong correlation between the perceived acceptability of various medication and lifestyle interventions and the perceived patient long-term adherence to such interventions. Overall, 59% of the respondents reported that more than 75% of their patients adhered to aspirin treatment. This figure amounted to 52% for ACE inhibitors, but to only 22,3% for polypills, 7,0% for physical activity programmes and 3,1% for healthy diet. Conversely, 21,6% of the responded reported that less than 25% of their patients adhered to smoking cessation. This figure amounted to 21,4% for polypills and 20,3% for health diet. It amounted to only 2-4% for single drug treatments.

Across regions, reported adherence tended to be slightly higher in South-East Asia and Western Pacific, and slightly lower in Africa. In the Americas, it tended to be slightly lower for lifestyle interventions, and higher than average for medical interventions. Across income groups, there tended to be a gradient from lower to higher income countries, whereby reported adherence was higher in higher income countries.

At policy level, excise taxes were reported to be available by 80,6% of all respondents for tobacco products; 76,3% for alcoholic beverages; 49,3% for e-cigarettes, but only 32,7% for sugar sweetened beverages and 24,8% for other unhealthy commodities. Of note, more than one in four respondents did not know whether an excise tax was in place for e-cigarettes, a figure that amounted to more than one in three for other unhealthy commodities. Excise taxes were most frequently reported in Europe, South-East Asia and Western Pacific for tobacco products, e-cigarettes and alcoholic beverages. This is in contrast with sugar sweetened beverages and other unhealthy commodities, where excise taxes were more frequently reported in Africa, South-East Asia and in the Americas. Across income groups, excise taxes were more frequently reported in higher income countries for tobacco products, e-cigarettes and alcoholic beverages. Conversely, excise taxes for sugar sweetened beverages and other unhealthy commodities tended to be more frequently reported in lower-income countries.

With regard to bans on advertising, promotion and sponsorship, total bans were reported to be in place by 41,9% of respondents for tobacco products, and by 26,4% for e-cigarettes. Total bans were much less frequent for alcoholic beverages, sugar sweetened beverages and other unhealthy

commodities. Partial bans were reported to be in place by 14,9% of respondents for tobacco products and 29,1% for e-cigarettes and 45,5% for alcoholic beverages. “No bans” was most frequently reported for sugar sweetened beverages (63,8%). Total or partial bans on advertising, promotion and sponsorship were more frequently reported to be in place in higher-income countries for tobacco products and e-cigarettes; in low-income countries for alcoholic beverages and in upper-middle income countries for sugar sweetened beverages and other unhealthy commodities.

62,8% of all respondents reported that localized guidelines were in place in their country. Across regions, such localized guidelines were most frequent in Western Pacific (95%), followed by Europe (88,2%) and South-East Asia (75%), the Americas (57,3%) and Africa (47,5%). There was a strong gradient across income groups: HICs: 90%; LICs: 44,1%.

Section 3: Barriers and solutions

Overall, the level of agreement with the barriers identified in the previous iteration of the WHF Roadmap for secondary prevention was only moderate. On a 1-5 Likaert scale, the unavailability of priority lifestyle intervention programmes was perceived to be the most relevant roadblock (3,29), followed by the fact that “patients are not aware of the importance and need of long-term treatment” “do not follow recommendations” and that “priority lifestyle intervention programmes are not affordable”. At the other end of spectrum, most respondents disagreed with the statement that “priority medications (aspirin, beta blockers, ACE inhibitors and statins) are not available or not affordable (1,79 and 2,37 respectively). In open comments, other perceived roadblocks pertained to clinical inertia, lack of coordination, cultural barriers, distance to health facilities, myths about medication side effects, misinformation on Internet, long waiting times, lack of trained personnel, priorisation of economic considerations over health consciousness, lack of rehabilitation programmes, insufficient government focus on primary prevention, among others. Across income groups, there tended to be a gradient whereby HICs reported a lower level of agreement than LMICs and LICs. In some cases the level agreement reported by UMICs followed this gradient, in other instances UMIC respondents displayed a slightly higher level of agreement than LMICs. Across regions, suggested barriers were generally perceived to be more relevant in Africa and in the Americas, and less relevant in Europe, South-East Asia and Western Pacific.

Regarding solutions, the overall level of agreement with suggested approaches was quite high. The most relevant Health system related solutions were felt to be to “strengthen the role of the primary care health system level for cardiovascular secondary prevention” (4.32) followed by “integrating secondary prevention interventions with simple cardiac rehabilitation programmes” (4.29). The most relevant patient related solutions were felt to be to “educate patients (health literacy) (4,45), followed by the “use of information and communication technology to remind patients (4.38). Of note, on a 1-5 Likaert scale, all patient-related suggested solutions scored higher than 4. The most relevant Health care professional related solutions were perceived to be to “train and educate HCPs (4,37) to “provide audit and feedback solutions (4,25) and to “implement decision support systems (4,19). There was a slightly weaker consensus on “sharing or shifting the roles of health care providers to non-physician health workers” and on “relying on opinion leaders” (3,69). Finally, the most relevant solutions related to priority interventions (lifestyle and medication) were reported to be to “promote the use of good quality, safe and inexpensive generic medications” (4,31), to “ensure that priority interventions are available at the primary care or community level (4,31) and to “ensure that priority interventions are available at secondary care level (4,31). Of note, on a 1-5 Likaert scale, all suggested solutions related to priority interventions scored higher than 4. Across categories, the overall level of agreement was

slightly lower in Europe, however, support for suggested solutions remained quite high (on a 1-5 Likaert scale, the lowest score was for “improve efficiency of pharmaceutical distribution chain” with a score of 3,25). Across income groups, interestingly, agreement rates were in general slightly lower in HICs and in LICs. In LICs, there was a proportion of approximately 10% of respondents that tended to “strongly disagree” with many of the suggested solutions.

Other suggested solutions included patient education, banning the production and sale of harmful products, better use of IT systems, more group therapy sessions, policies to reduce salt intake, policies to promote safe and healthy environments (urban planning, food sales), telehealth and e-learning for health care professionals, UHC, among others.

WHF Roadmap for Secondary Prevention of Cardiovascular disease Consultation 2022

Survey summary based on 268 responses

Q2: In which WHO Region do you live and work?

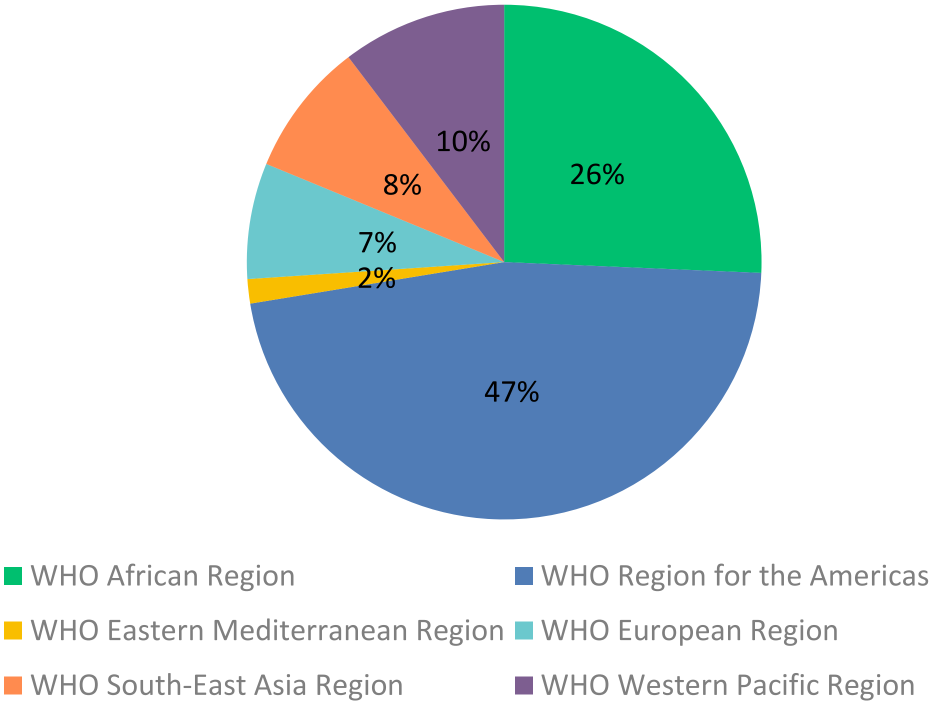

Q3: In which country do you live and work?

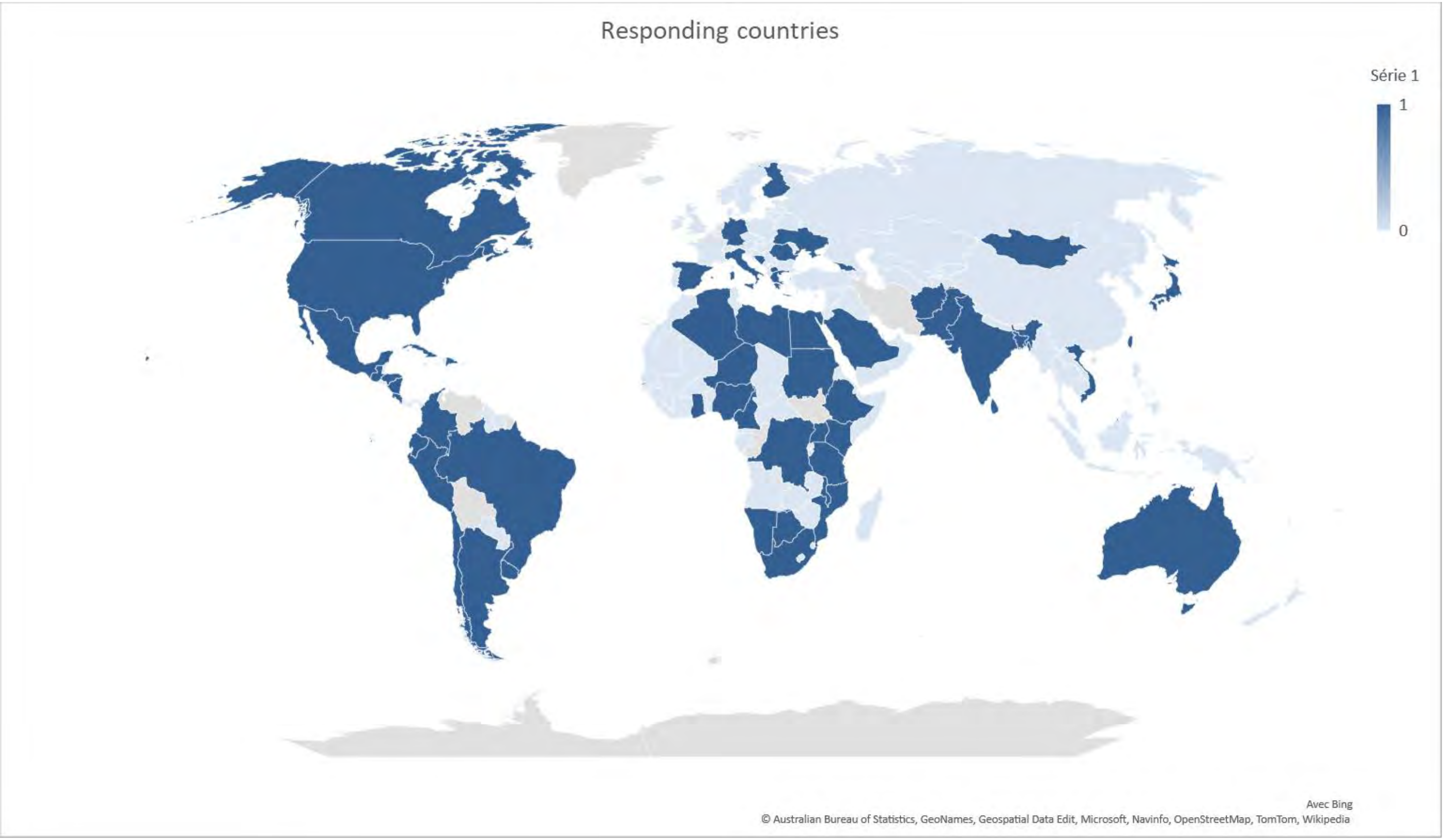

Country	Responses
Mexico	38
Australia	28
Argentina	18
Venezuela (Bolivarian Re	18
Cuba	13
Kenya	10
Nigeria	10
Japan	9
Chile	8
Namibia	7
Germany	6
Ghana	6
United Republic of Tanza	6
Dominican Republic	5
Ethiopia	5
Guatemala	5
Brazil	4
Uruguay	4
Bosnia and Herzegovina	3
Ecuador	3
Egypt	3
India	3
Mauritius	3
Pakistan	3
Uganda	3
United States of America	3
Bangladesh	2
Brunei Darussalam	2
Cameroon	2
Georgia	2
Malawi	2
Mozambique	2
Saudi Arabia	2
Sudan	2
Afghanistan	1
Algeria	1
Botswana	1
Canada	1
Colombia	1
Congo	1
Costa Rica	1
Finland	1
Gambia	1
Greece	1
Honduras	1
Italy	1
Libya	1
Mongolia	1
Nicaragua	1
Niger	1
Peru	1
Romania	1
Singapore	1
South Africa	1
Spain	1
Sri Lanka	1
The former Yugoslav Republic of Macedonia	1
Ukraine	1
Vietnam	1
Taiwan	1

Q4: Which income category does your country belong to? If you are unsure, you can check on the map below or here

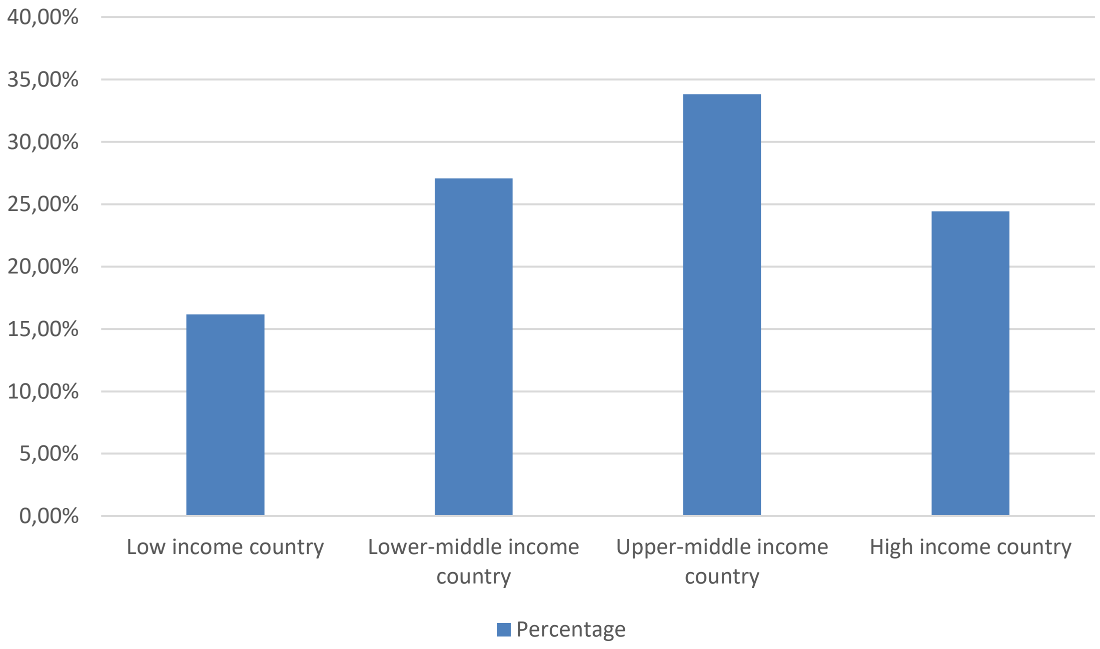

Q5: Which best describes your work setting?

Answered: 267 Skipped: 1

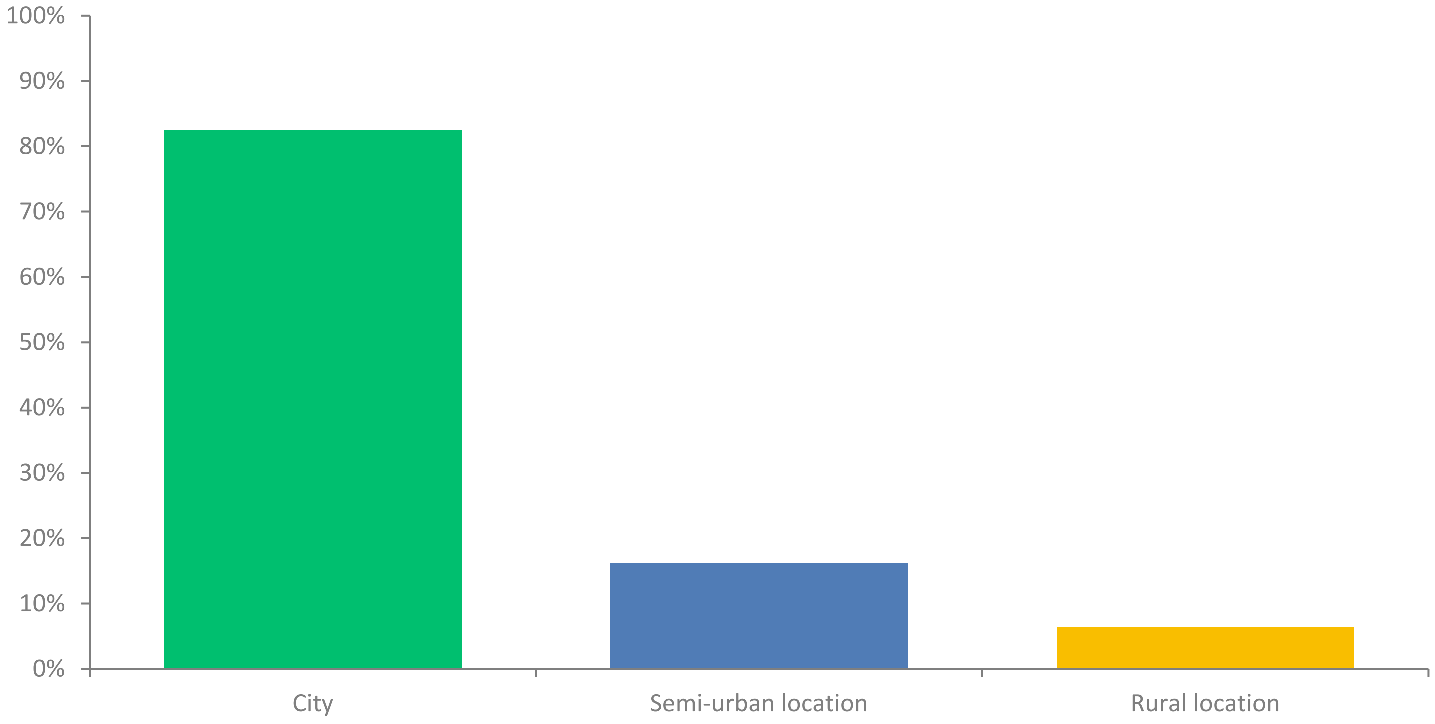

Q6: Setting where you practice/work at least 50% of your time:

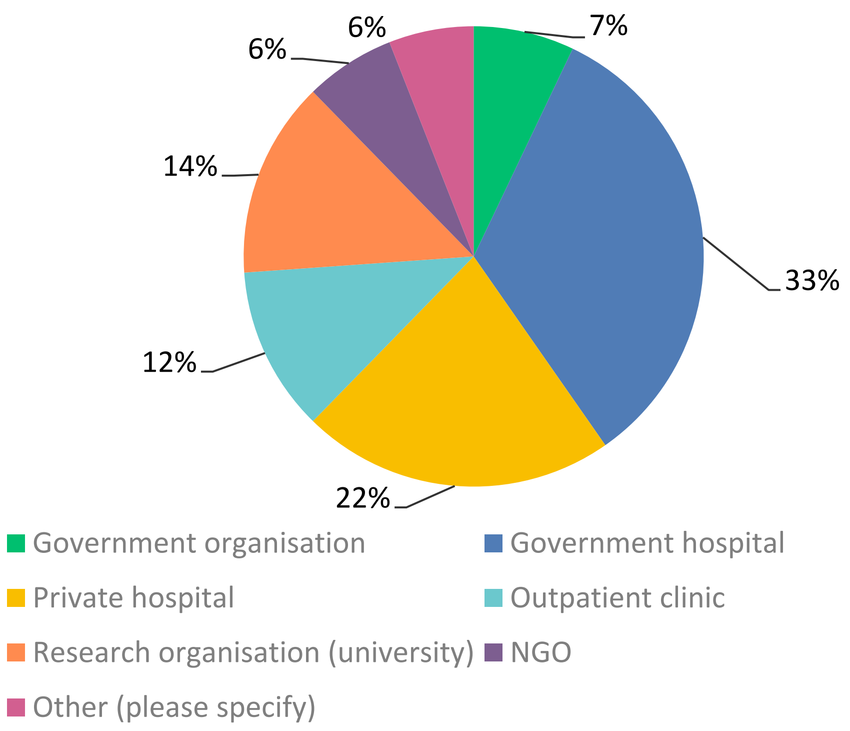

Q7: Occupation:

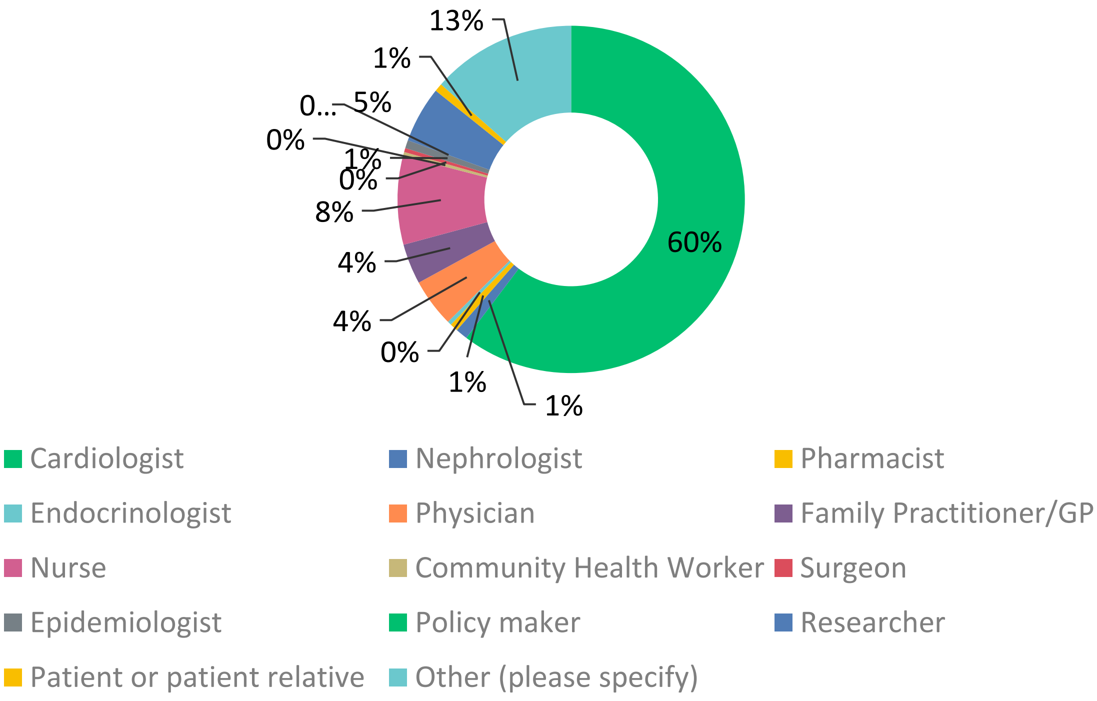

Q9: The following priority secondary prevention medications

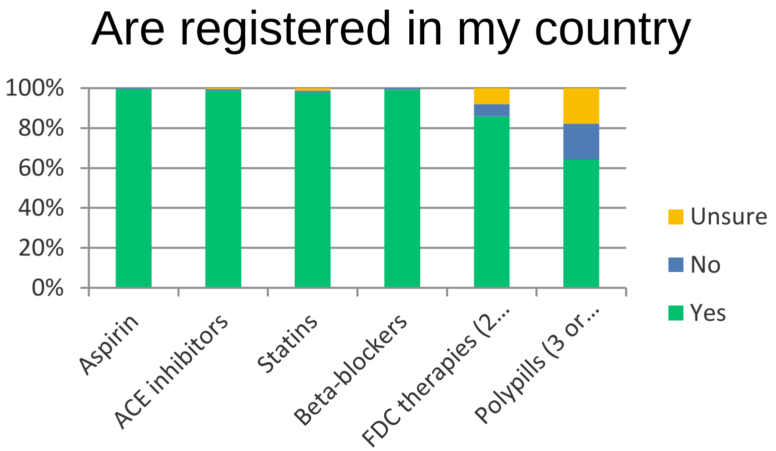

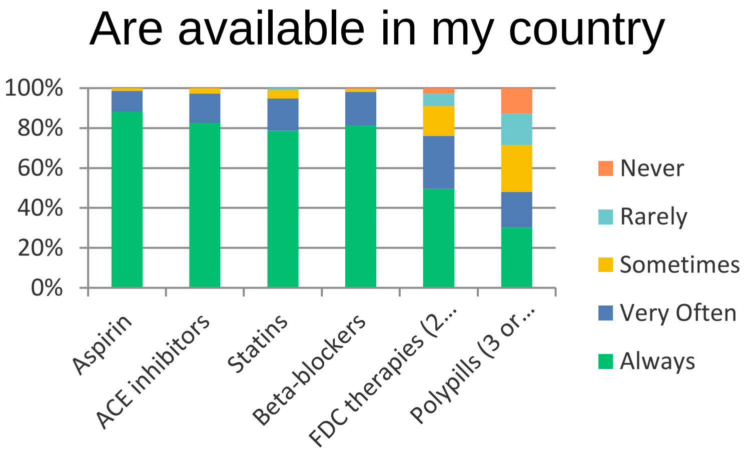

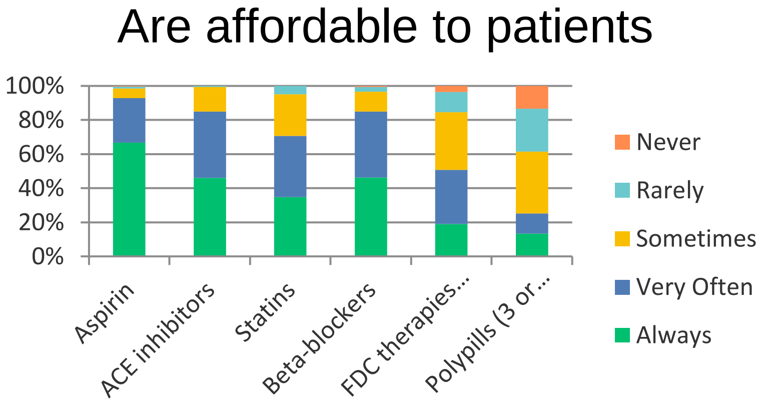

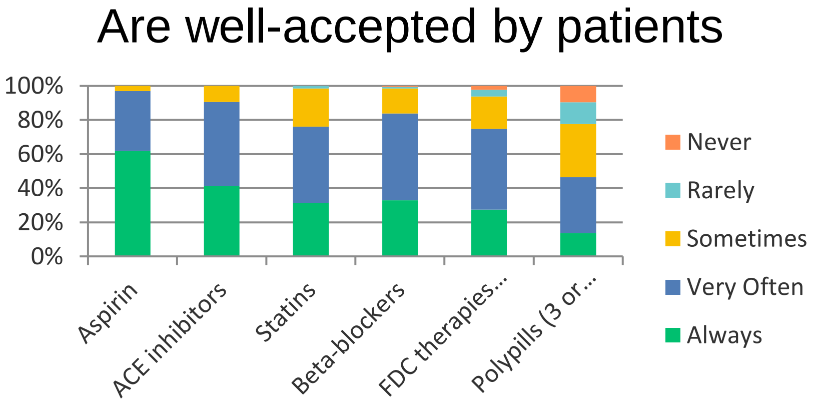

Q9: The following priority secondary prevention medications

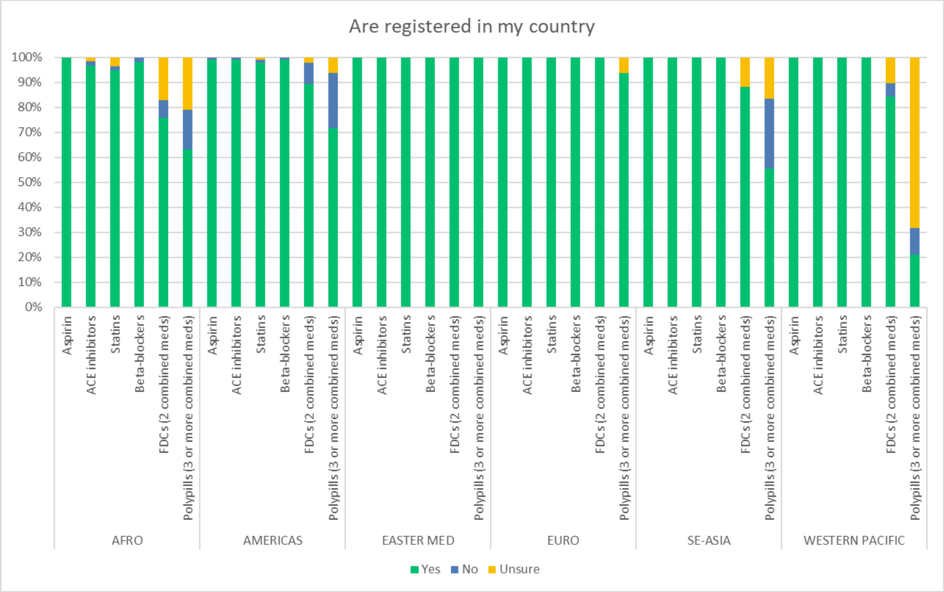

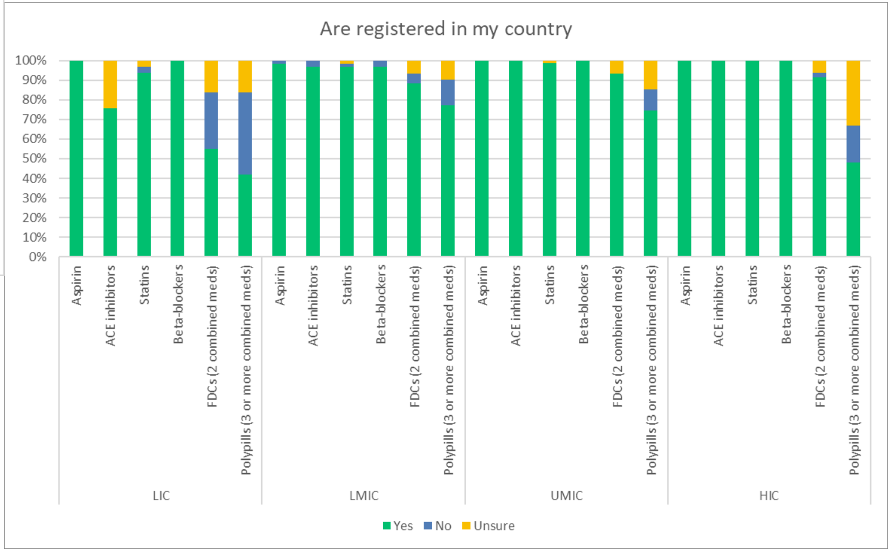

Q9: The following priority secondary prevention medications

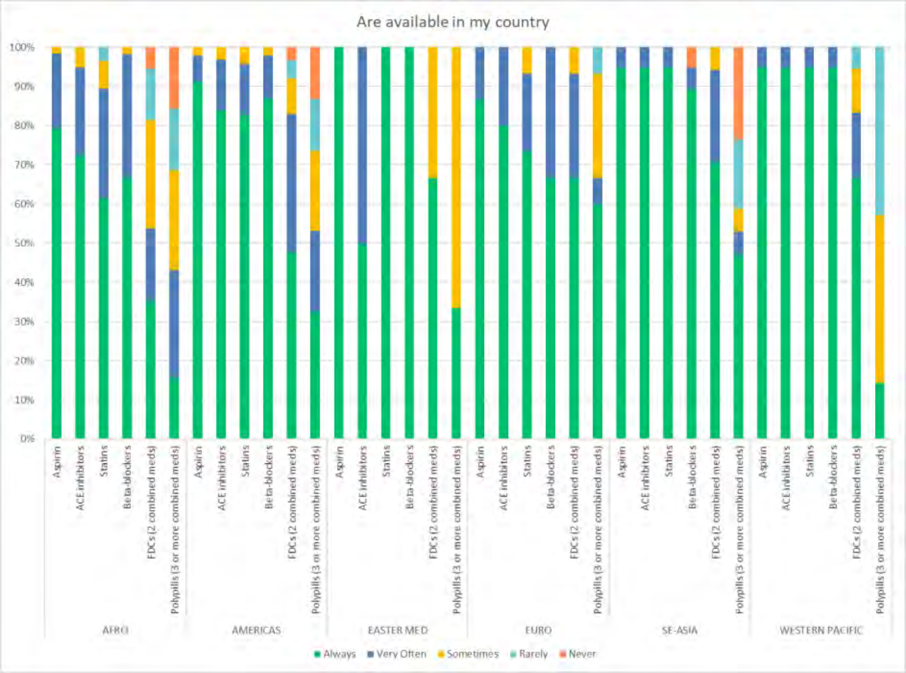

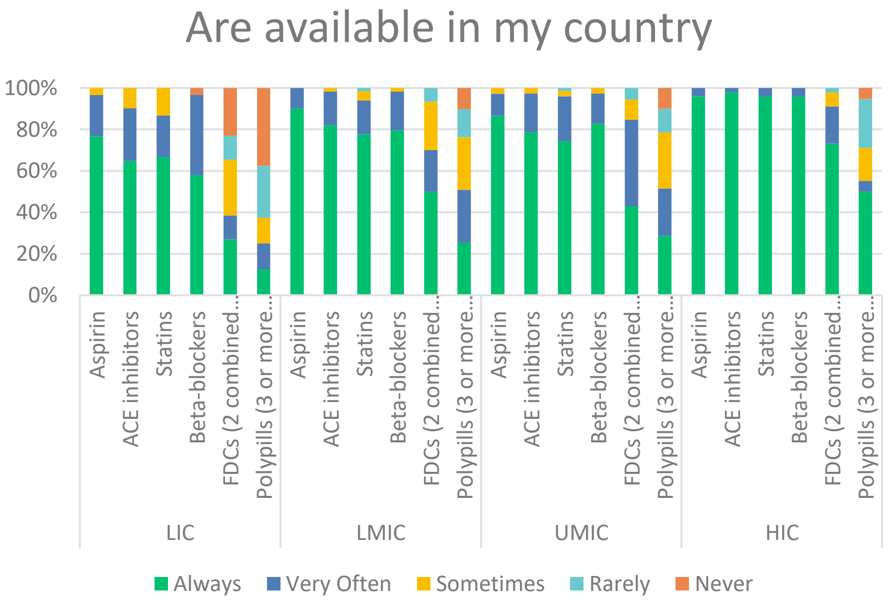

Q9: The following priority secondary prevention medications

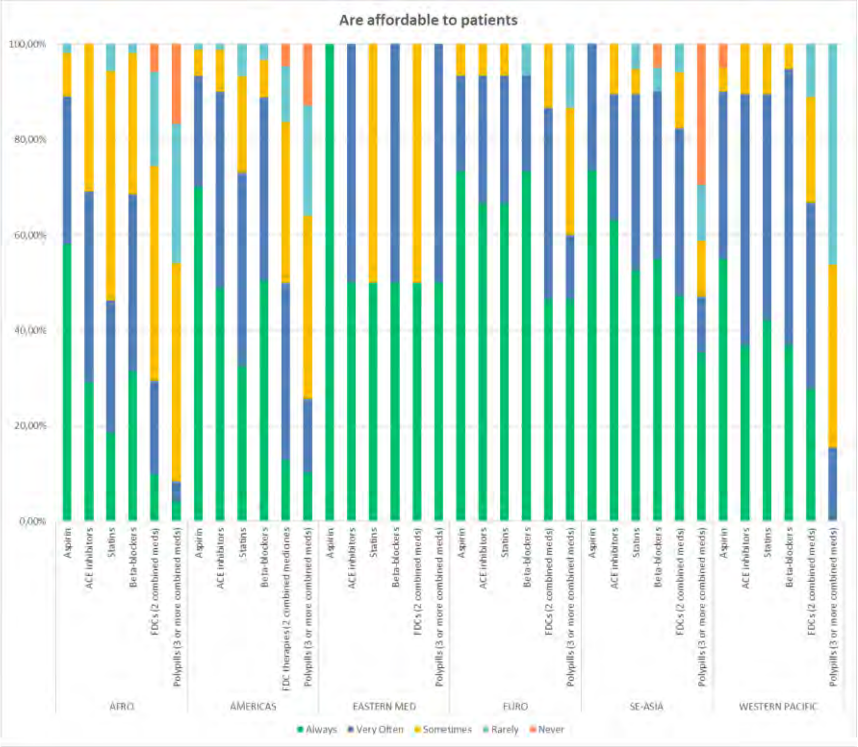

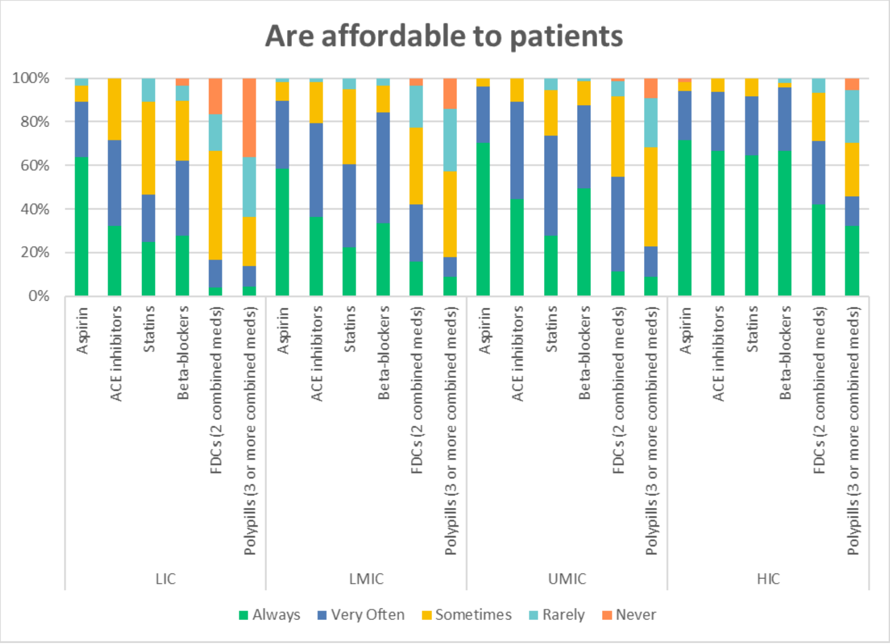

Q9: The following priority secondary prevention medications

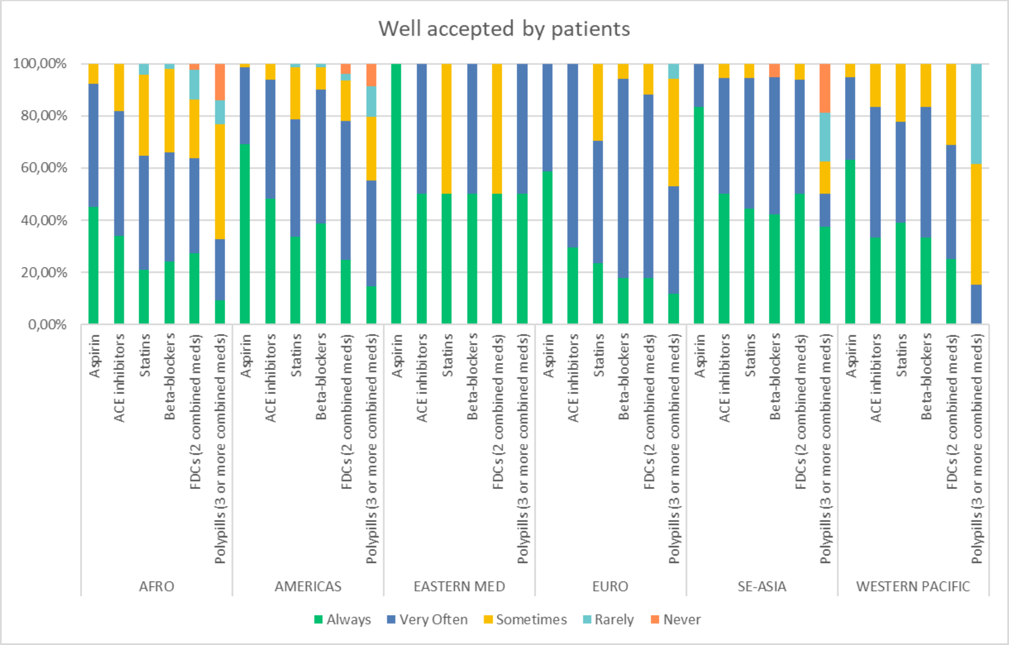

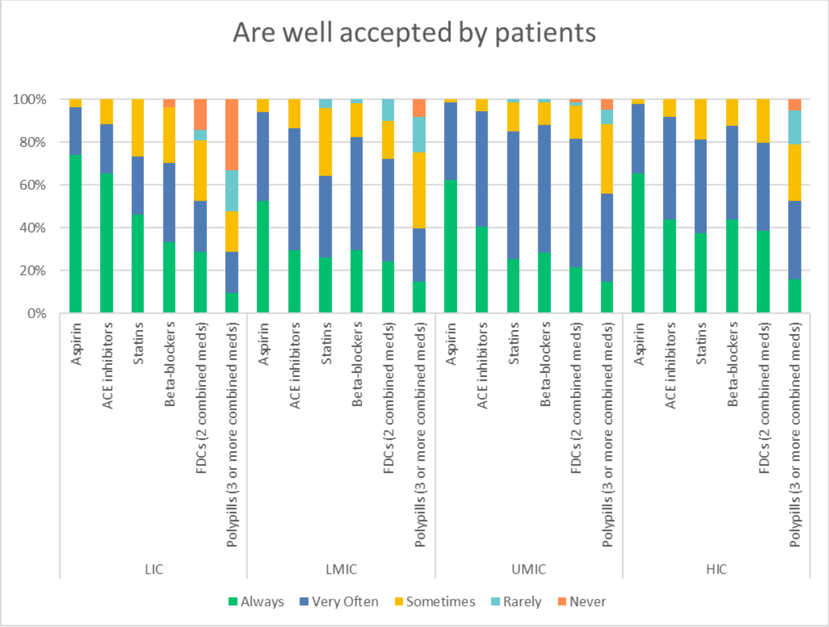

Q10: The following lifestyle interventions programmes

Are available in my country

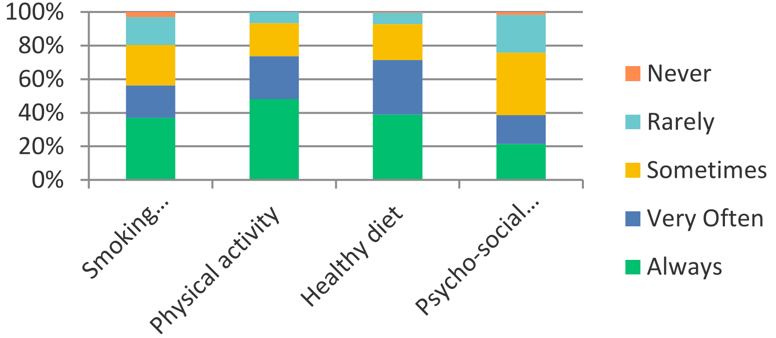

Are affordable to patients

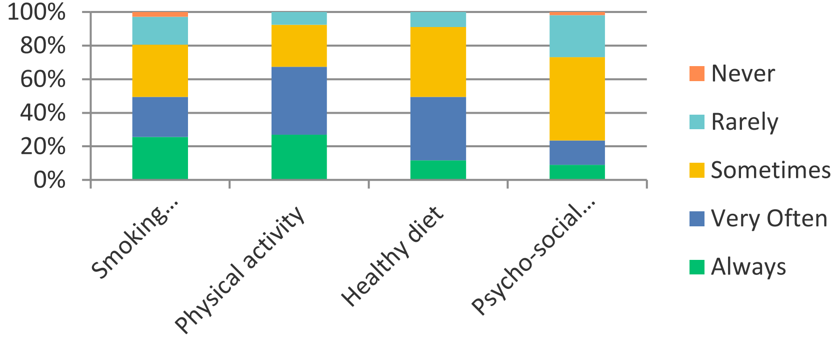

Are well-accepted by patients

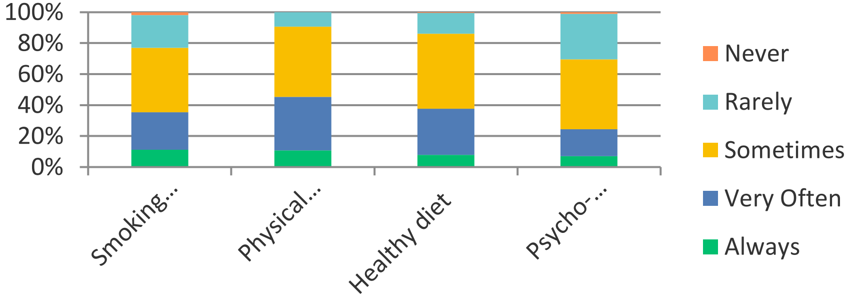

Q10: The following lifestyle interventions programmes

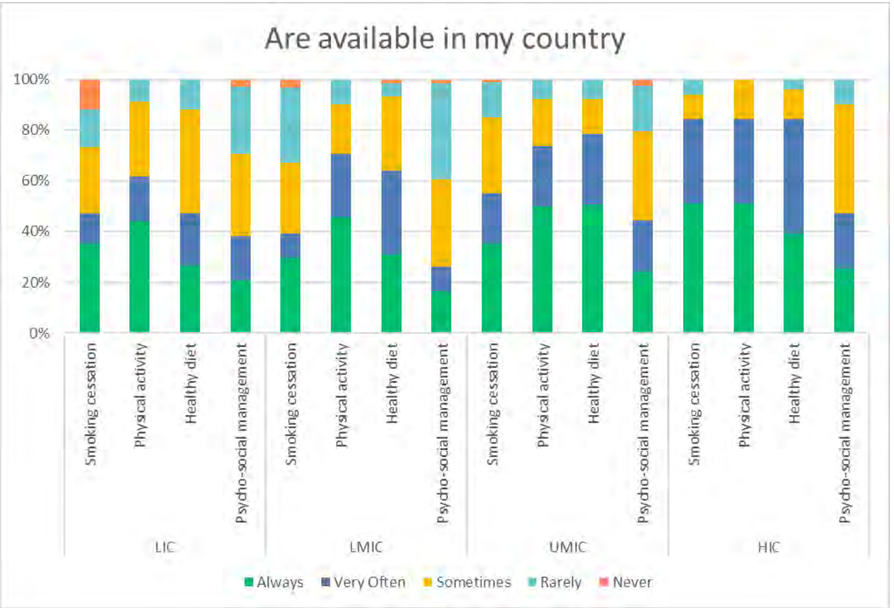

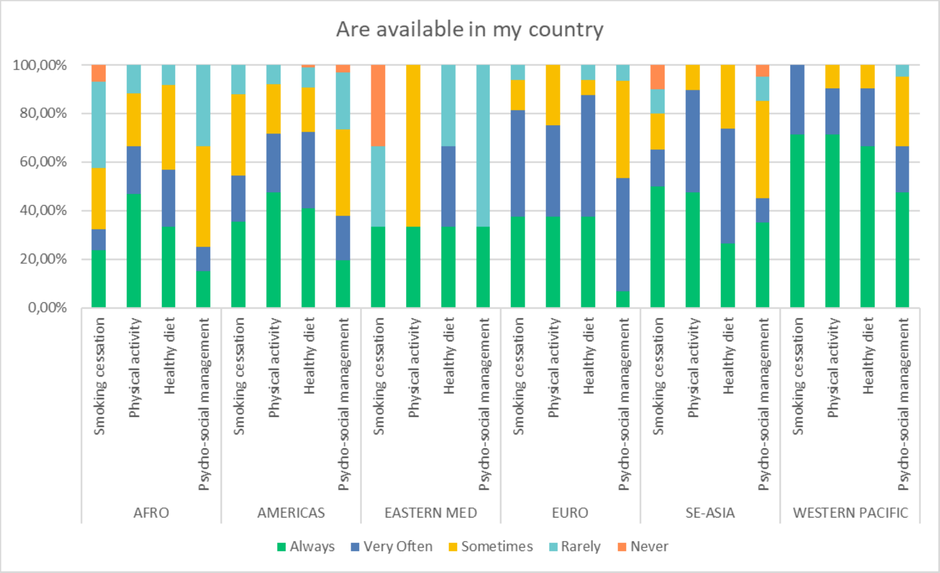

Q10: The following lifestyle interventions programmes

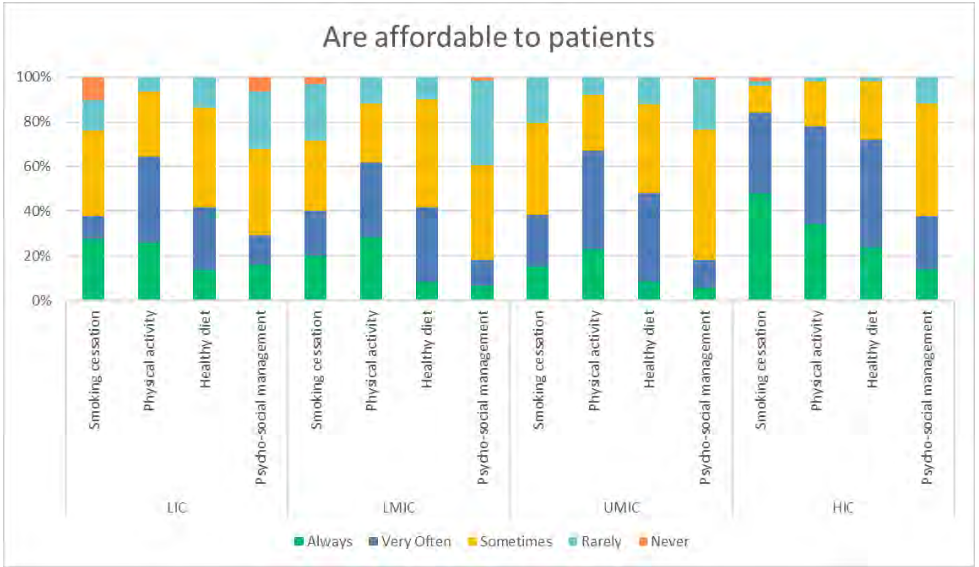

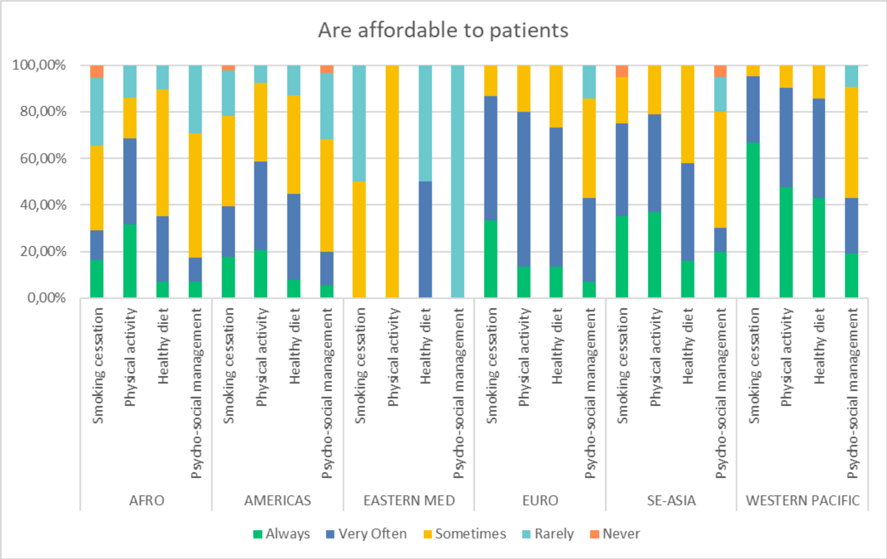

Q10: The following lifestyle interventions programmes

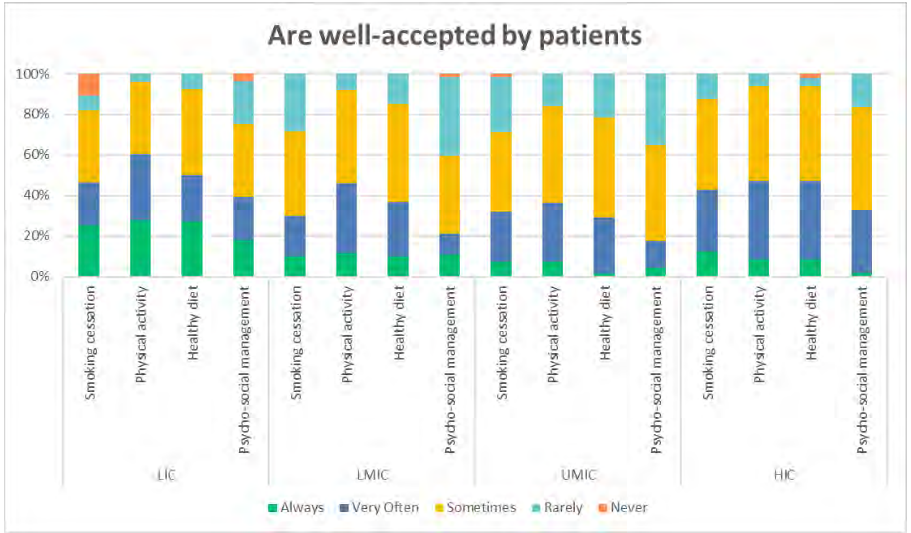

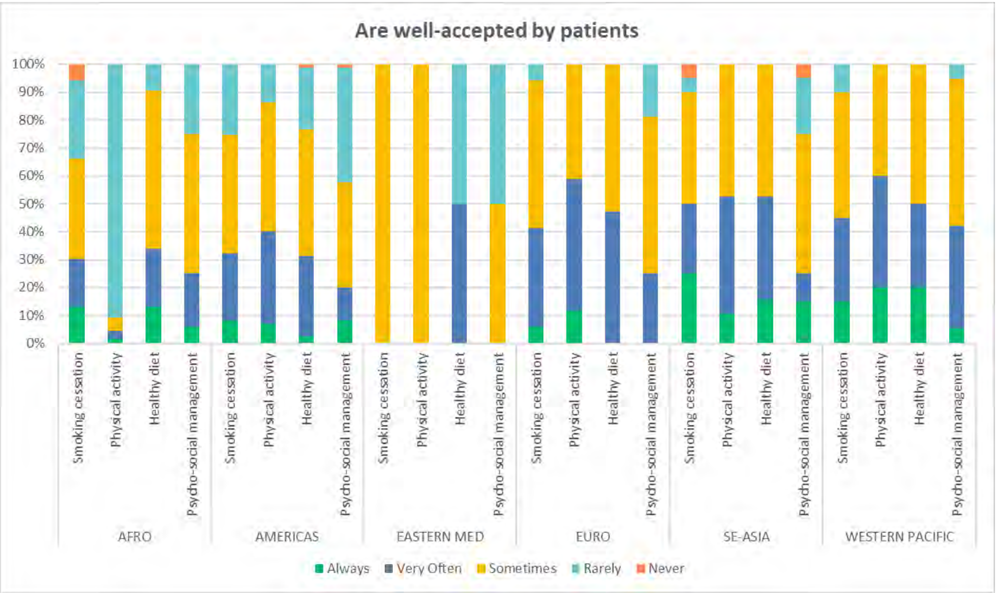

Q11: What percentage of your patients adhere in the long term to

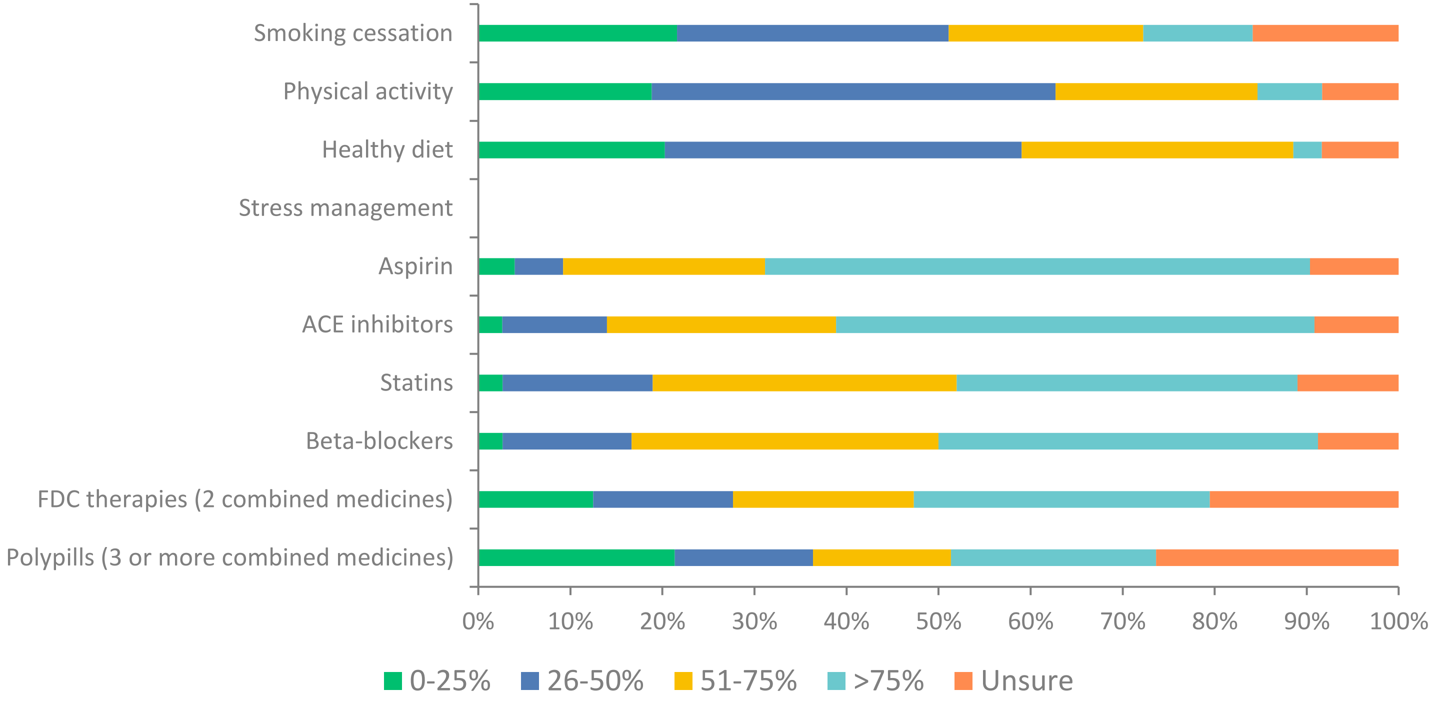

Q11: What percentage of your patients adhere in the long term to

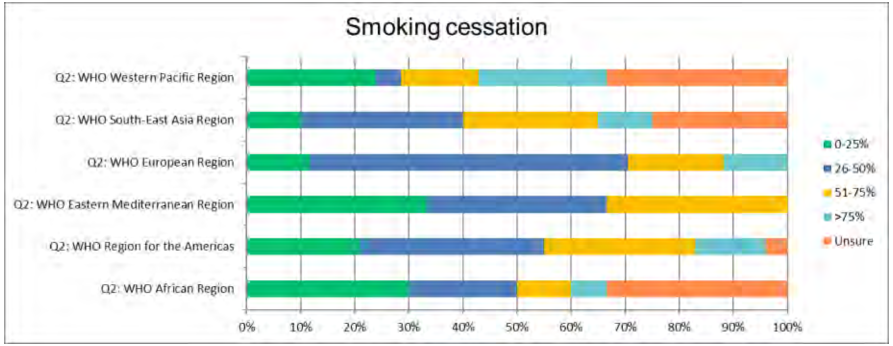

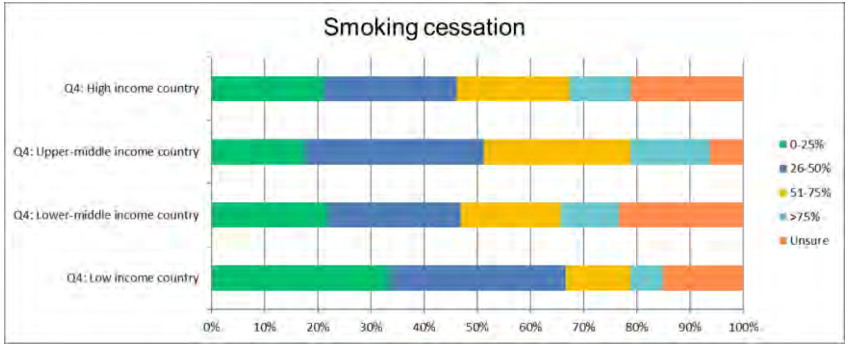

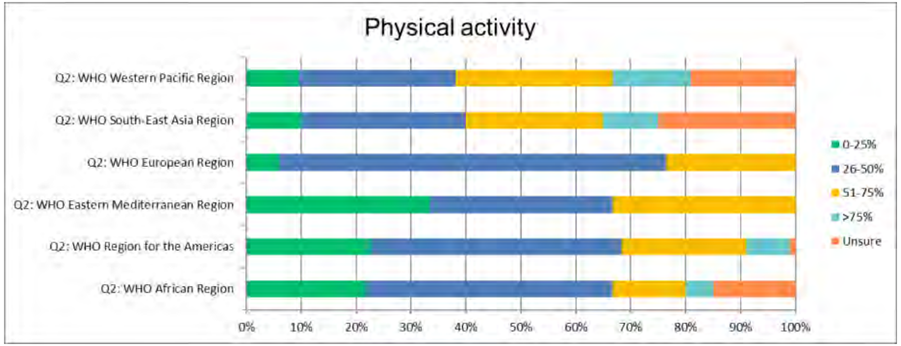

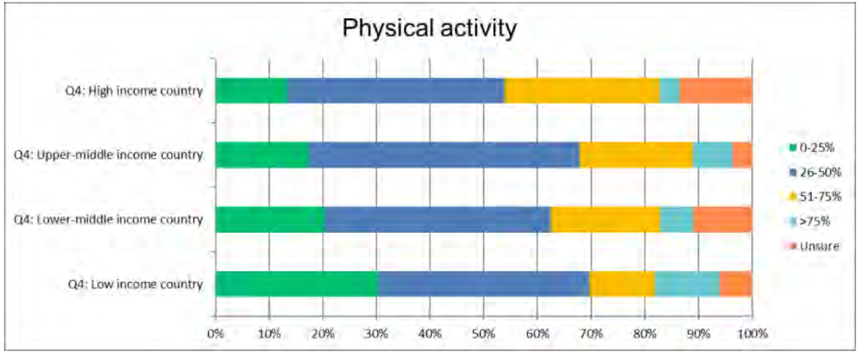

Q11: What percentage of your patients adhere in the long term to

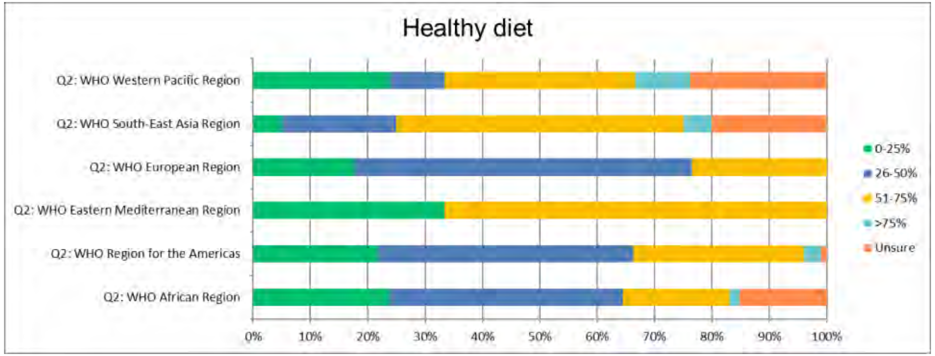

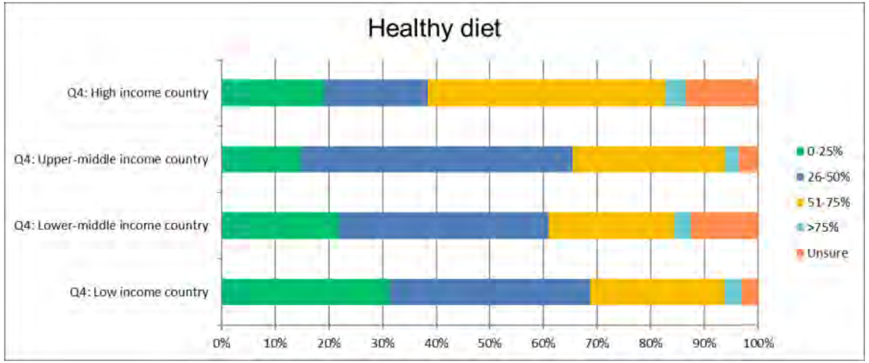

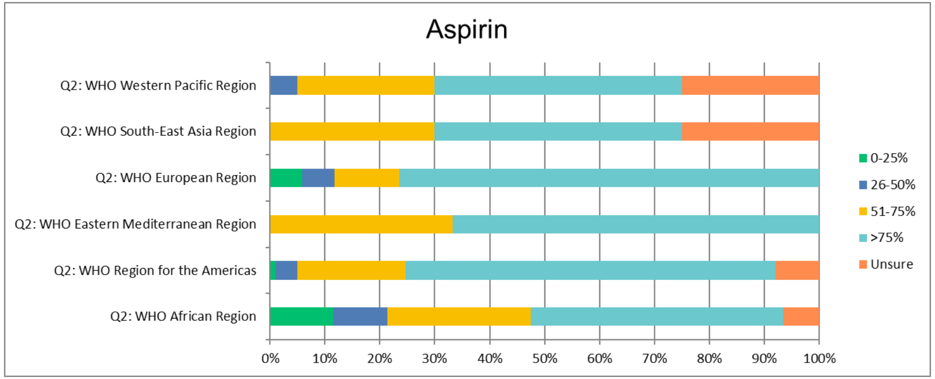

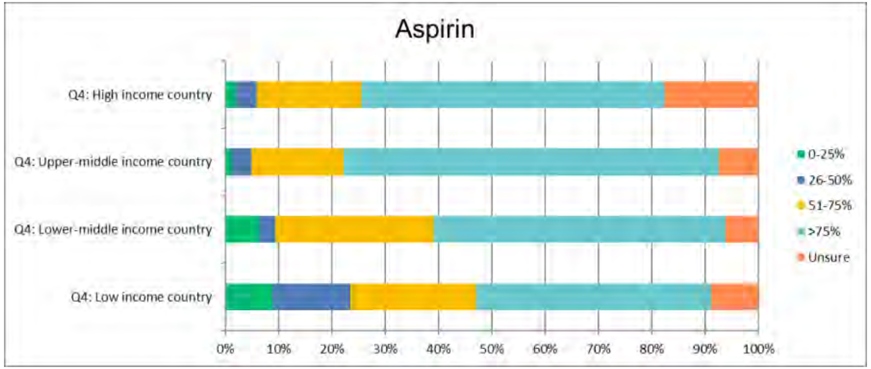

Q11: What percentage of your patients adhere in the long term to

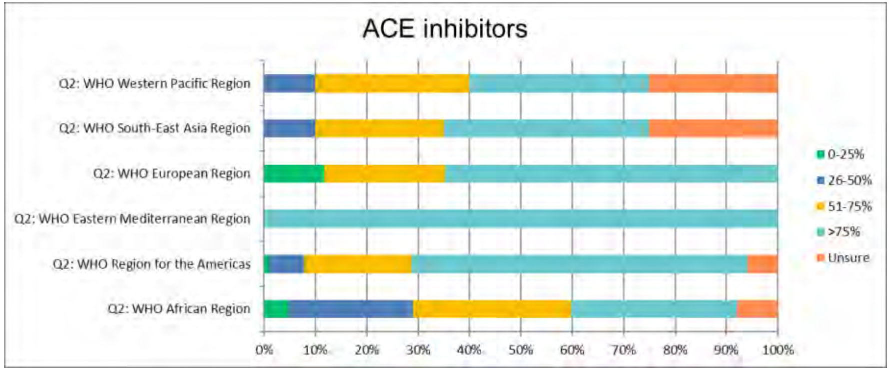

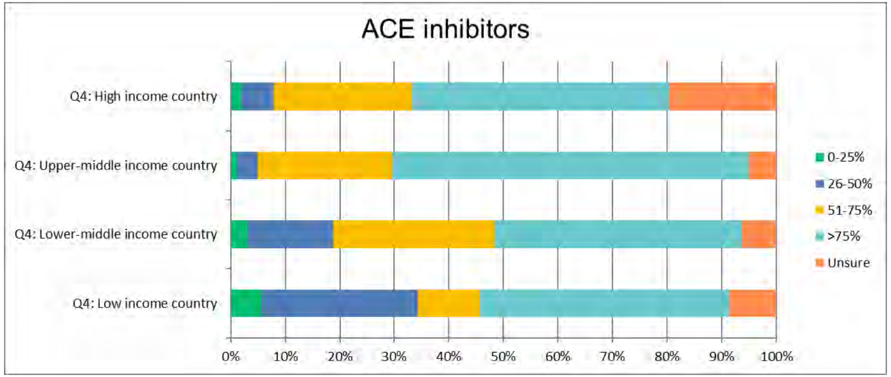

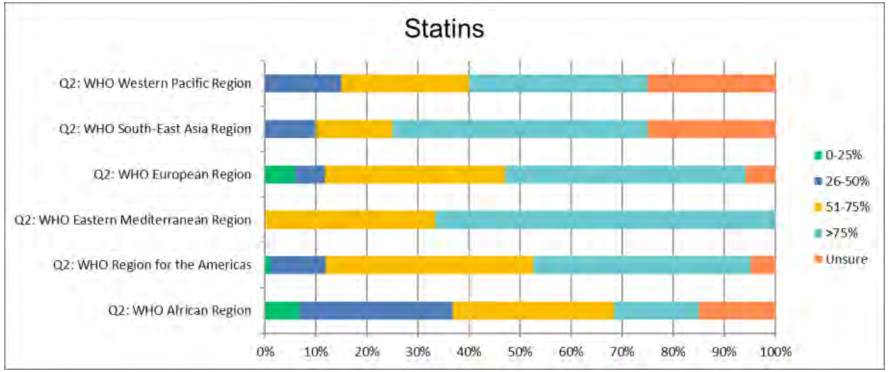

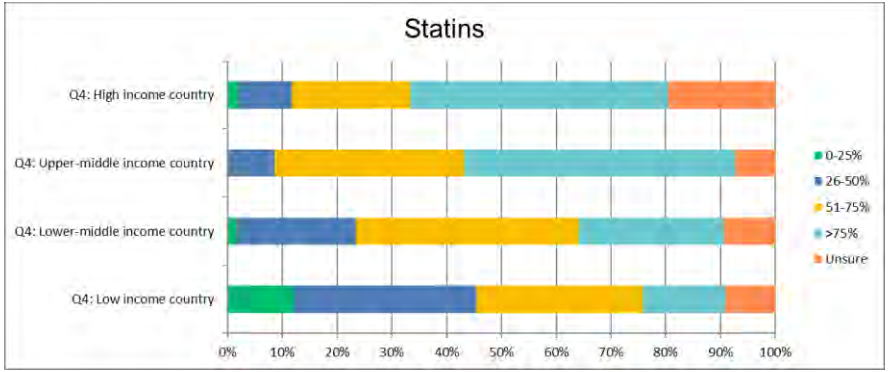

Q11: What percentage of your patients adhere in the long term to

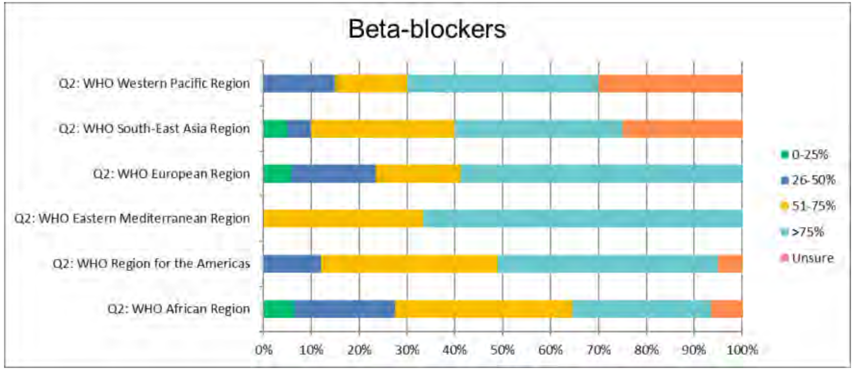

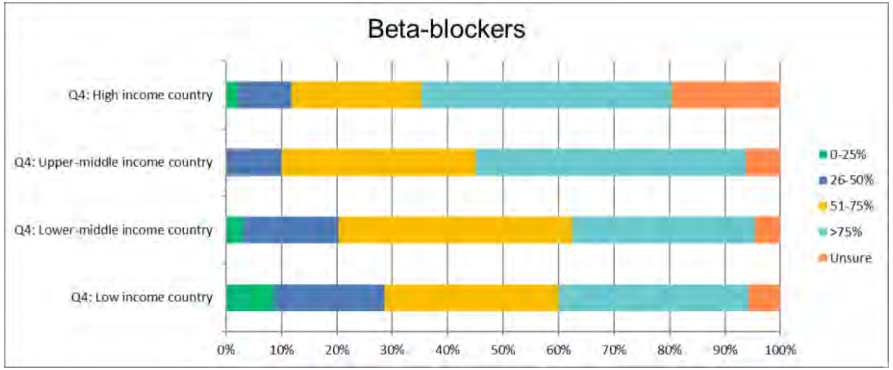

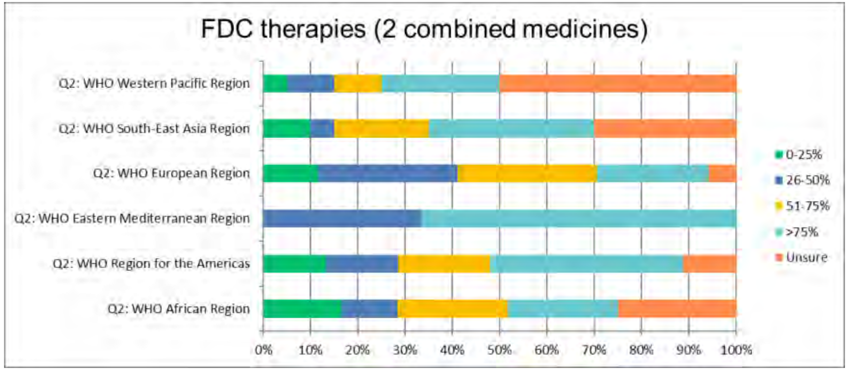

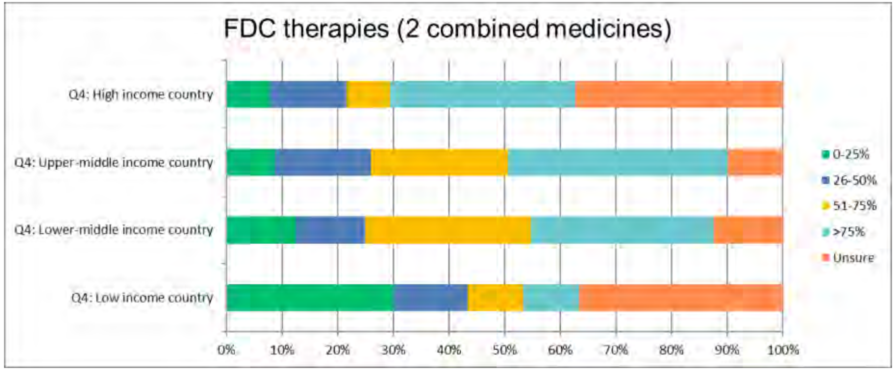

Q11: What percentage of your patients adhere in the long term to

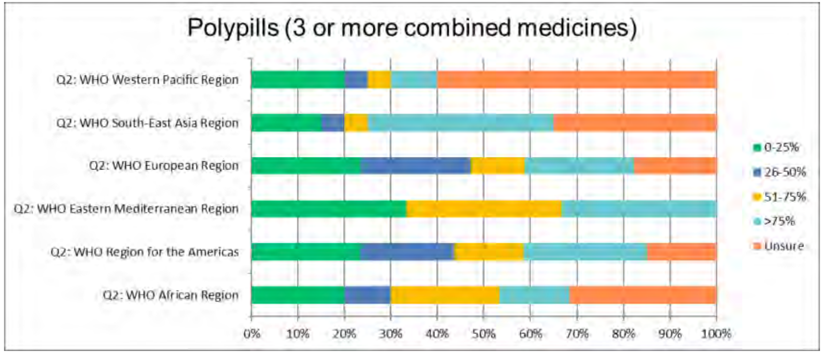

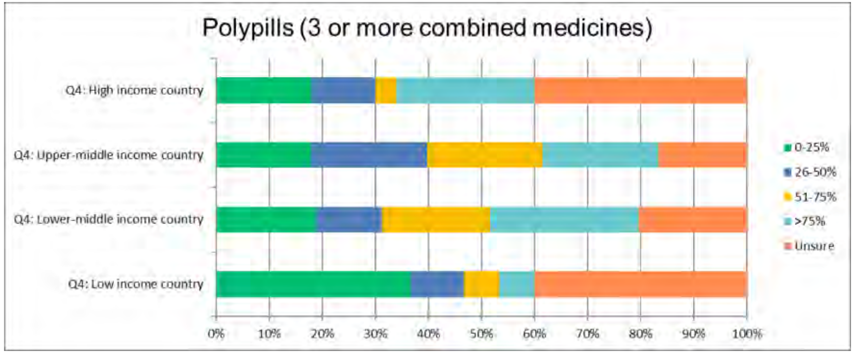

Q12: In your country, do you have excise taxes for

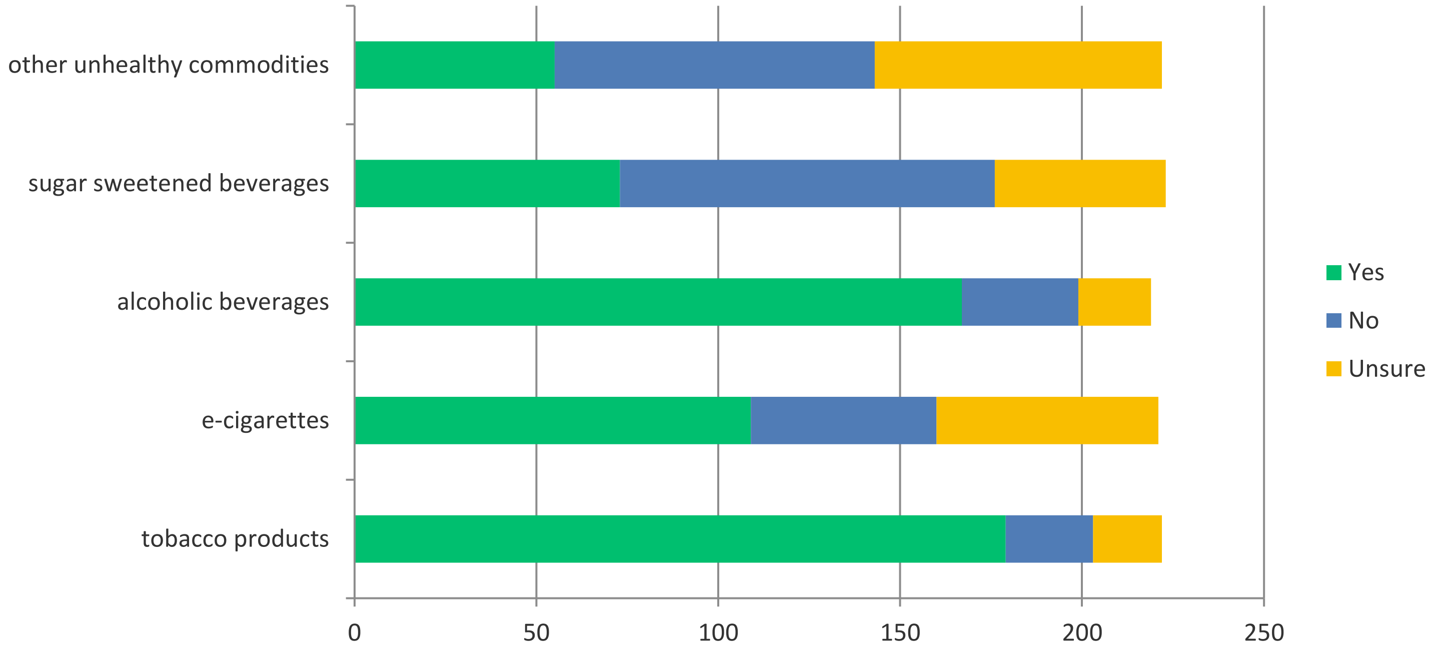

Q12: In your country, do you have excise taxes for

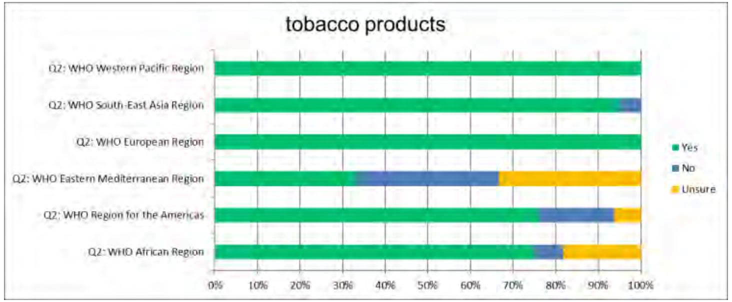

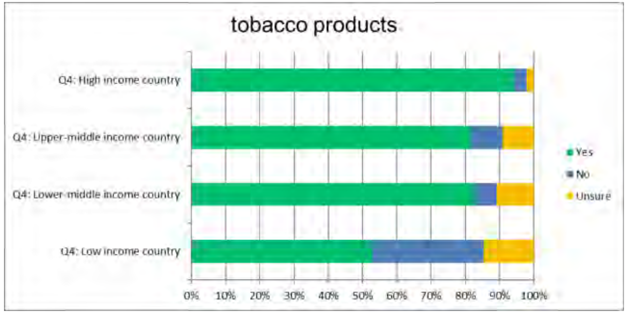

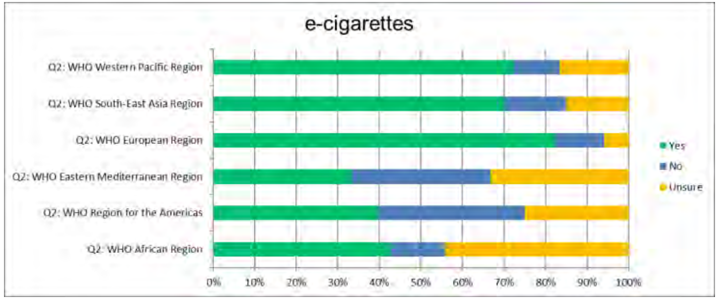

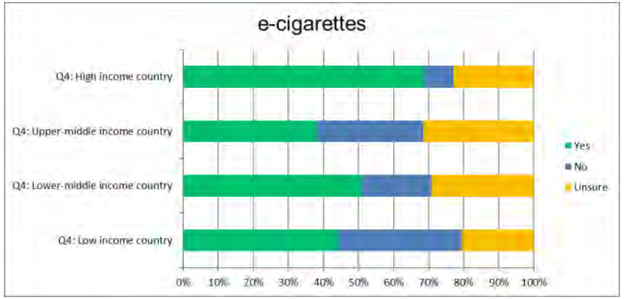

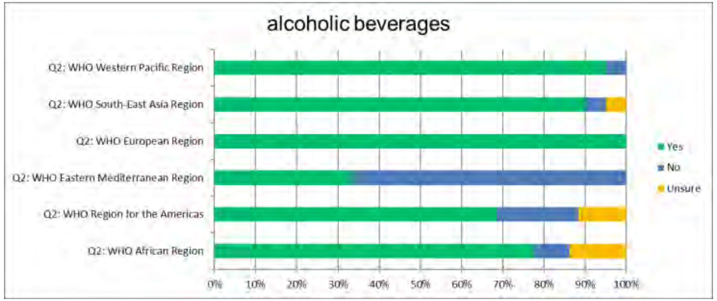

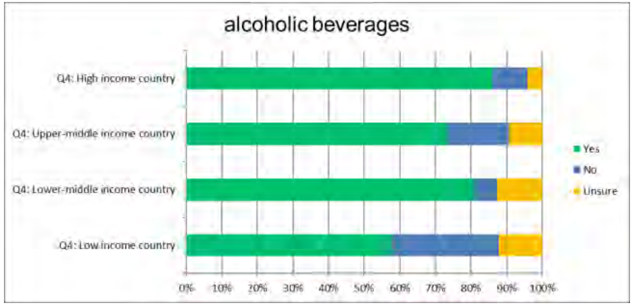

Q12: In your country, do you have excise taxes for

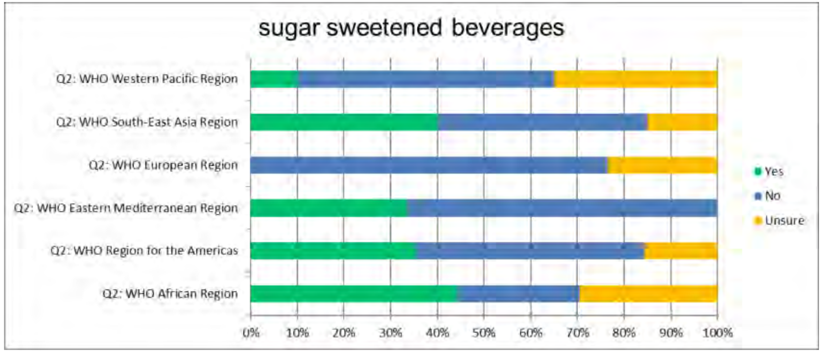

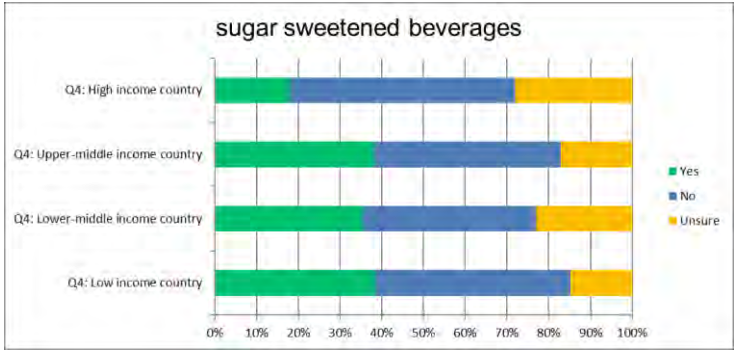

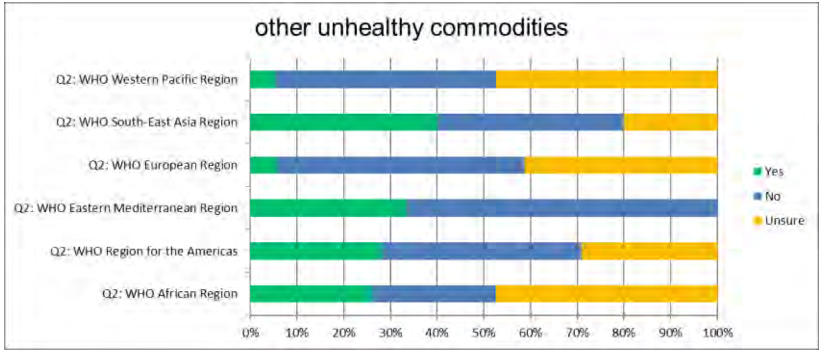

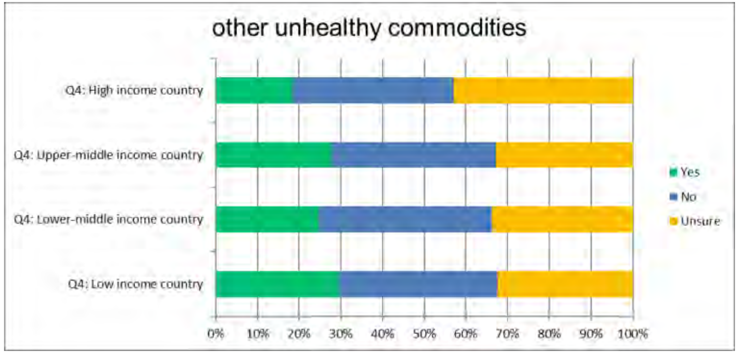

Q13: In your country, do you have comprehensive bans on advertising, promotion and sponsorship for

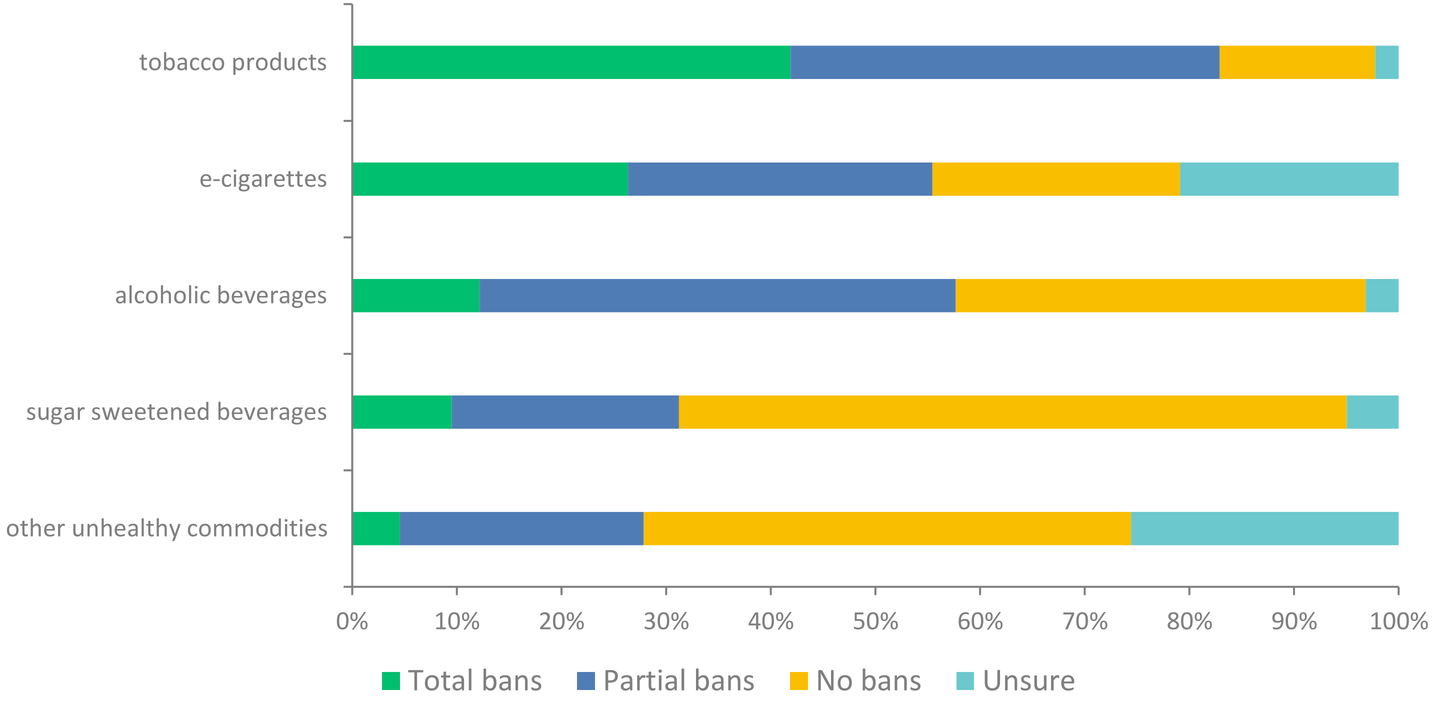

Q13: In your country, do you have comprehensive bans on advertising, promotion and sponsorship for

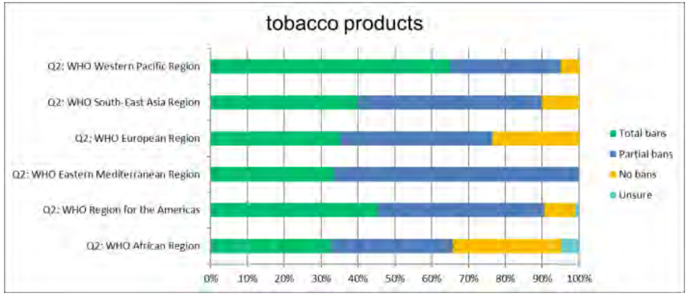

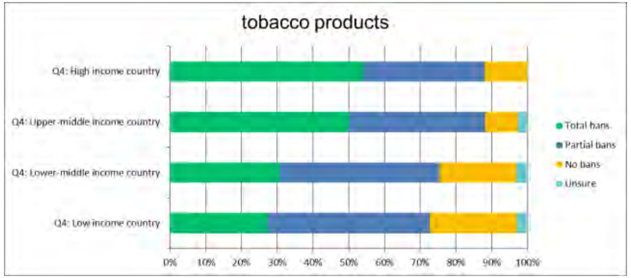

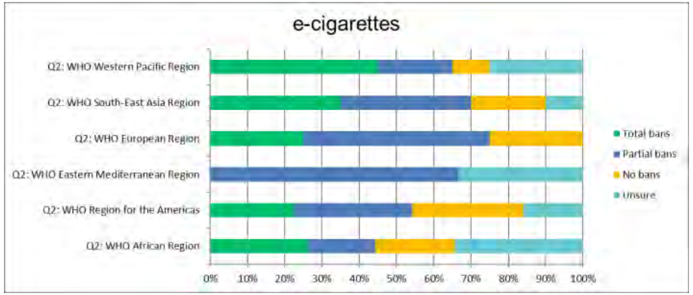

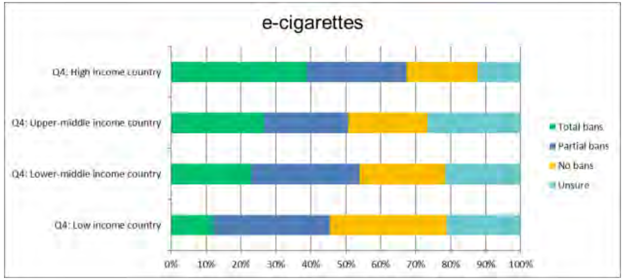

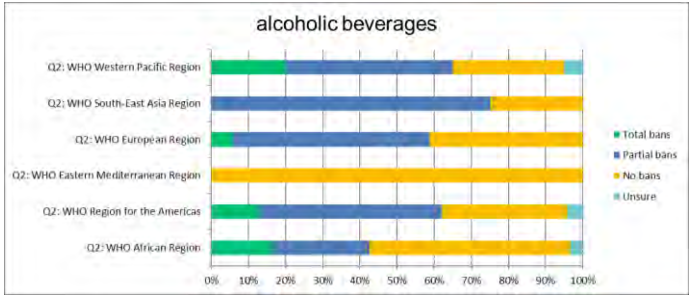

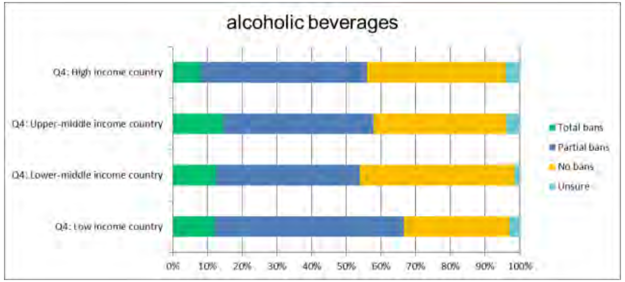

Q13: In your country, do you have comprehensive bans on advertising, promotion and sponsorship for

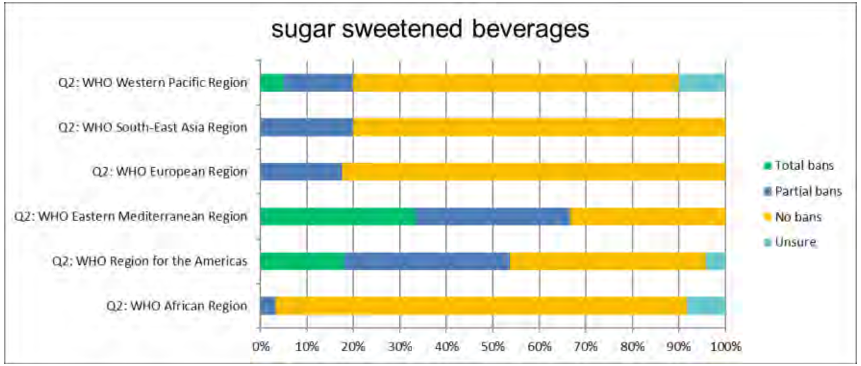

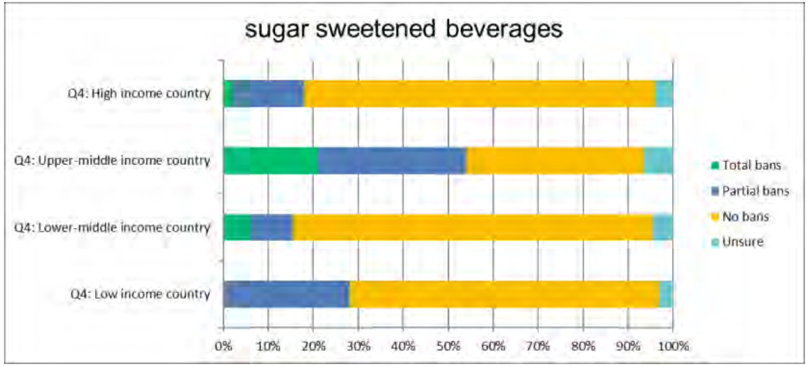

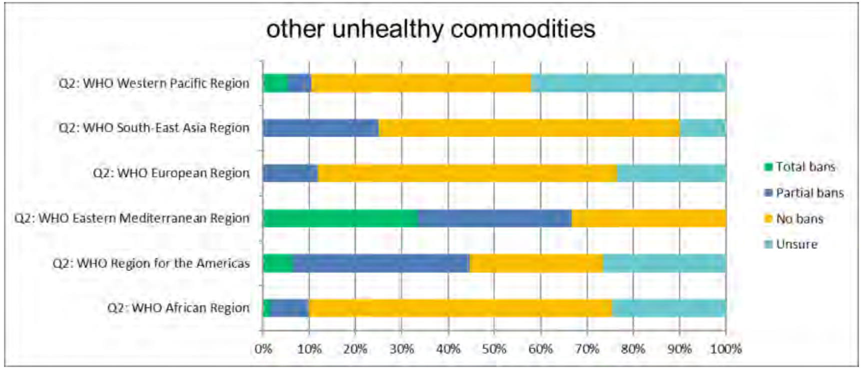

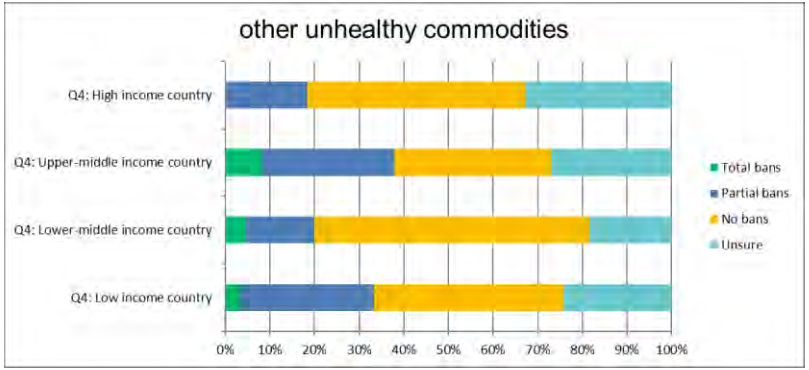

Q14: In your country, do you have specific (localized) guidelines for cardiovascular disease secondary prevention?

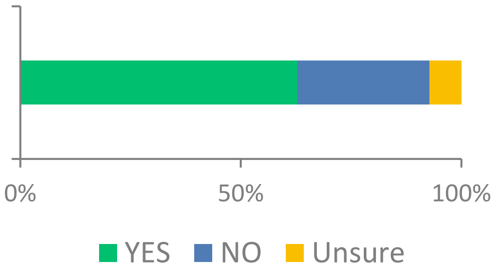

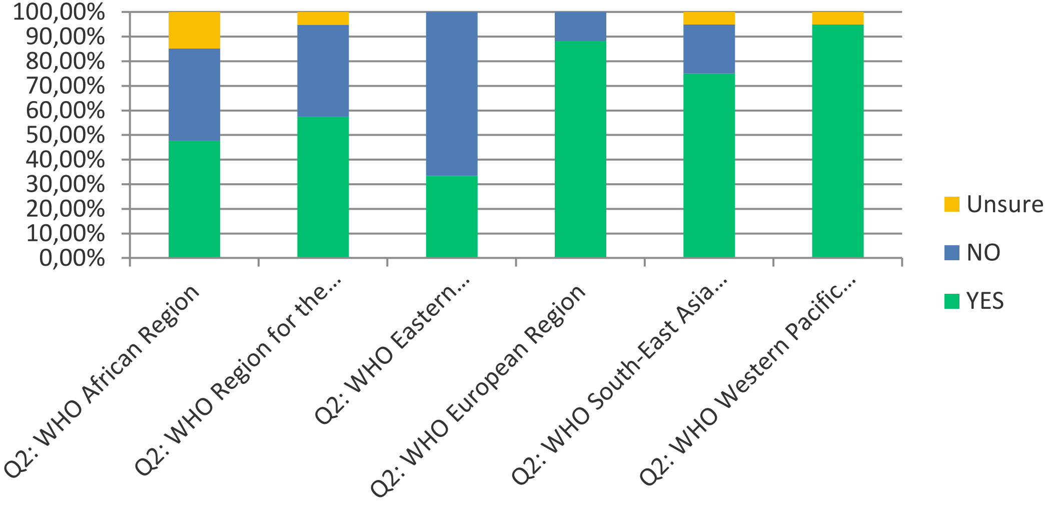

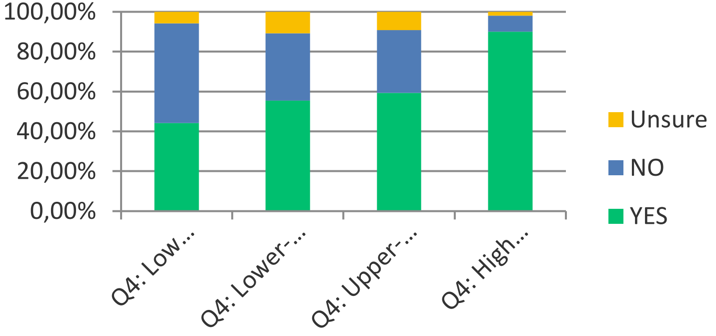

Q15: Please indicate the relevance of these suggested barriers in your setting.

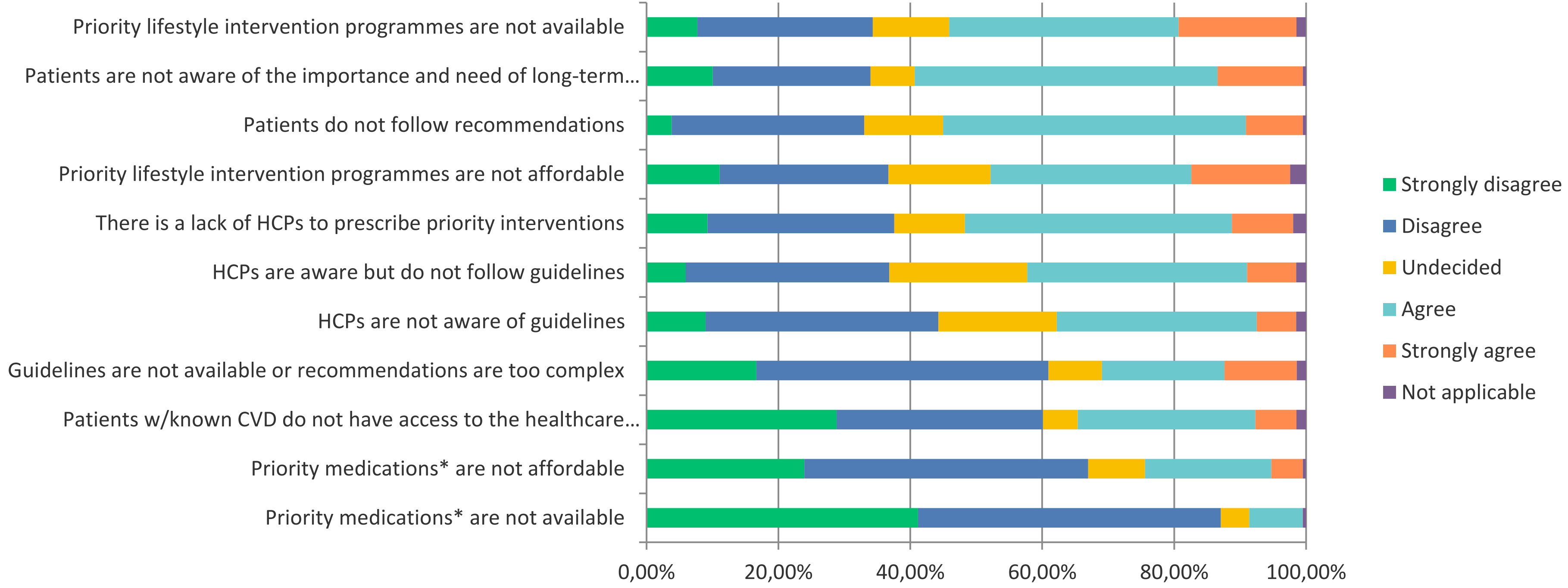

* (aspirin, beta blockers, ACE inhibitors, statins)

Q15: Please indicate the relevance of these suggested barriers in your setting.

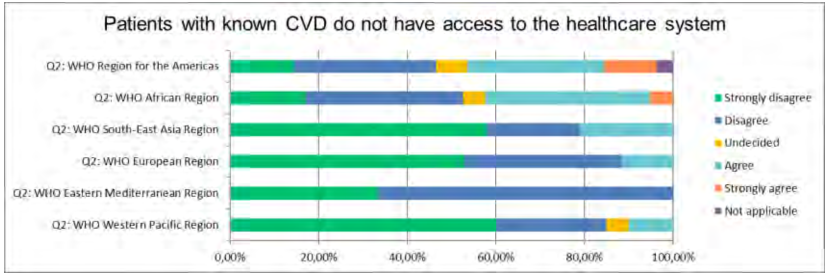

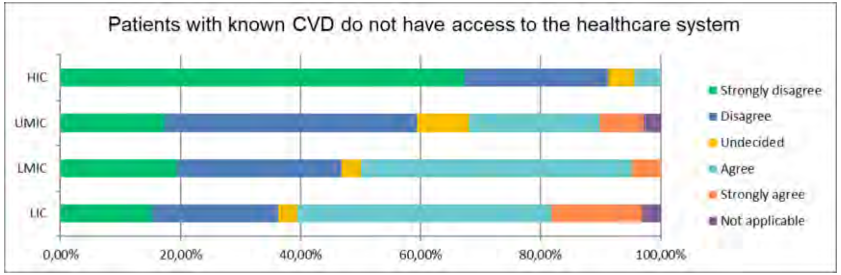

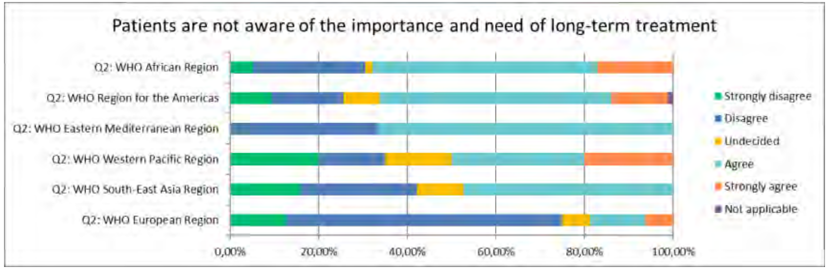

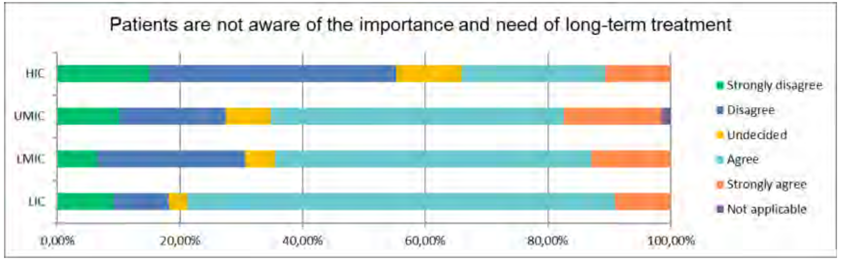

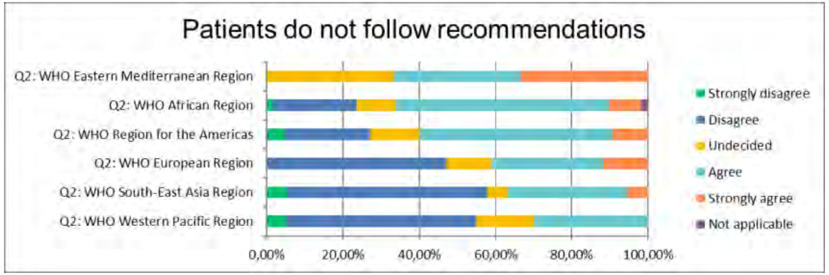

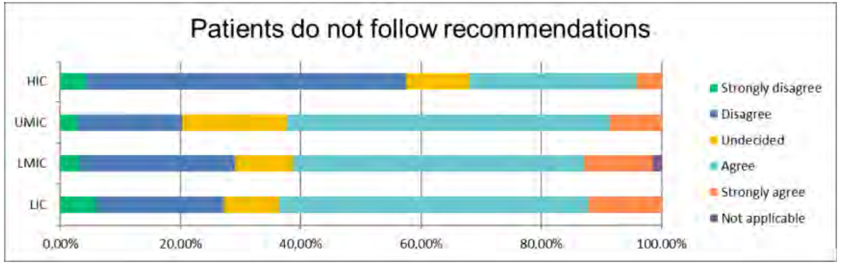

Q15: Please indicate the relevance of these suggested barriers in your setting.

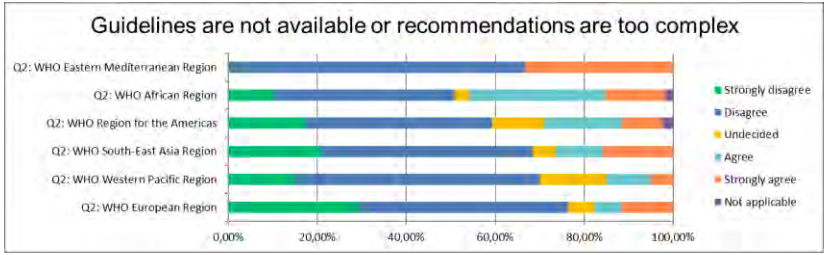

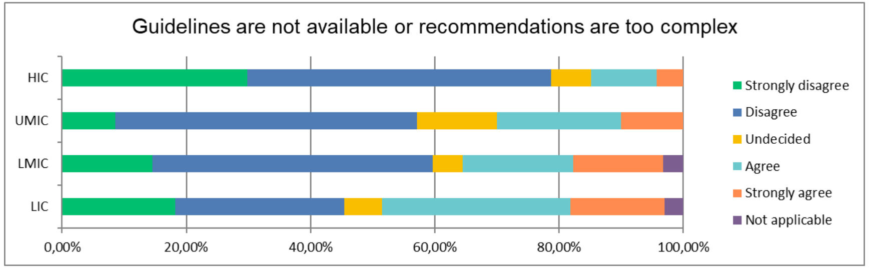

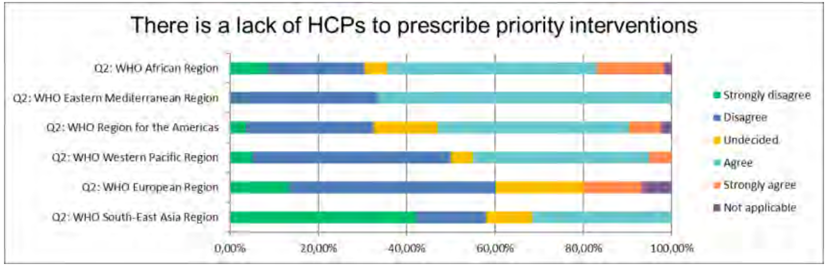

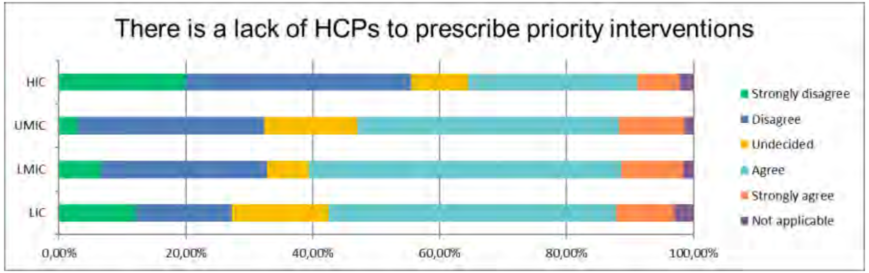

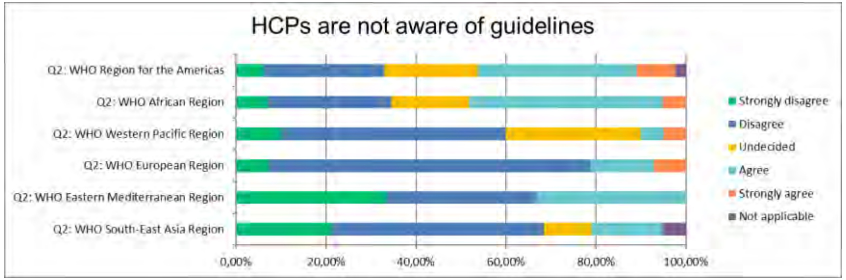

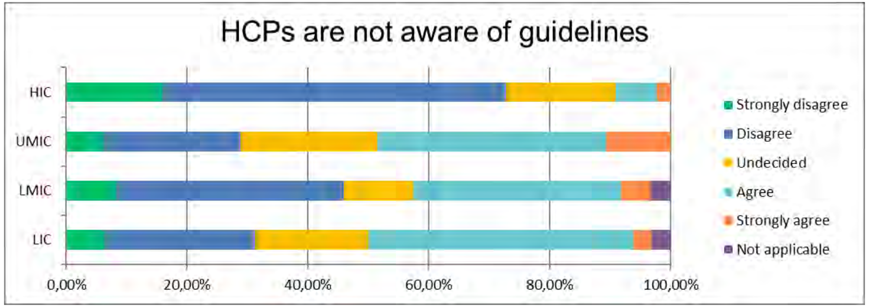

Q15: Please indicate the relevance of these suggested barriers in your setting.

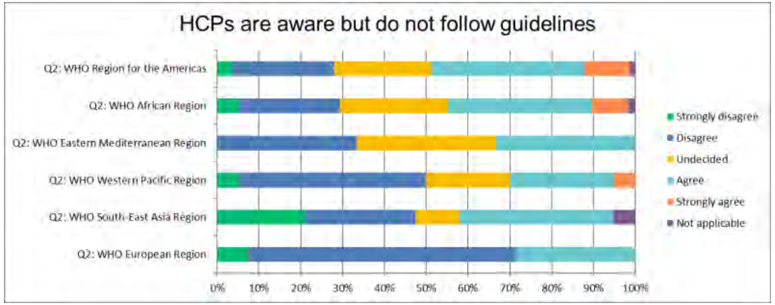

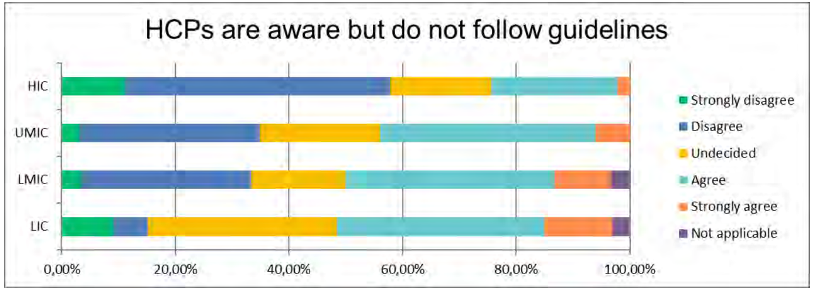

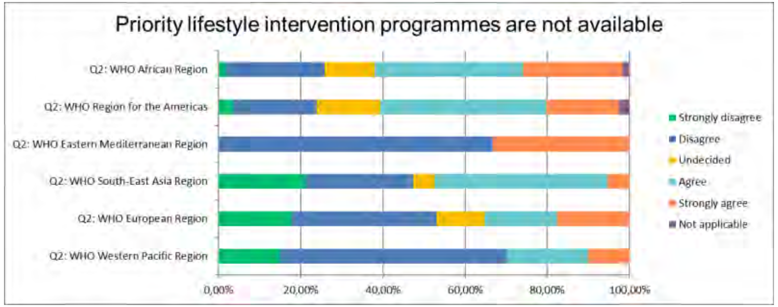

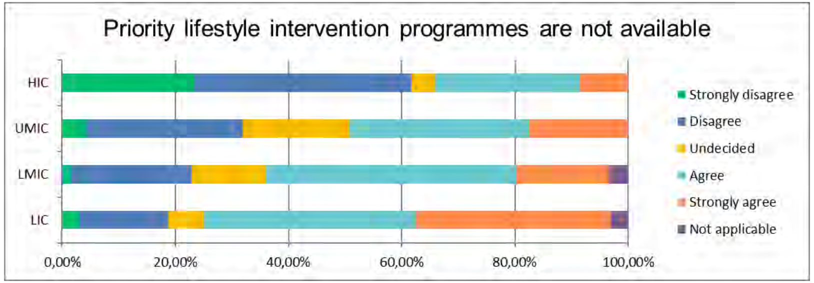

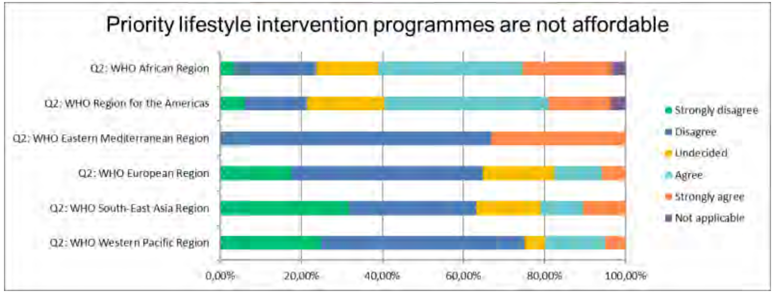

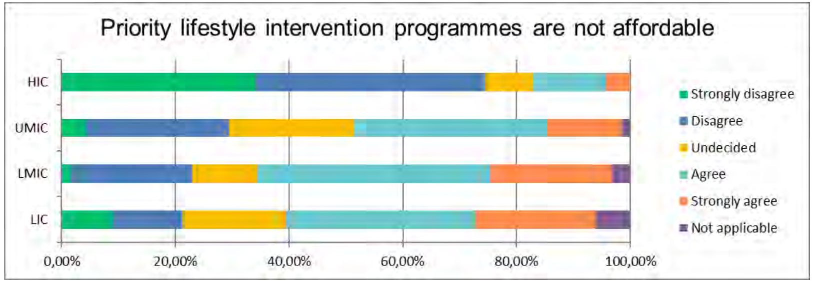

Q15: Please indicate the relevance of these suggested barriers in your setting.

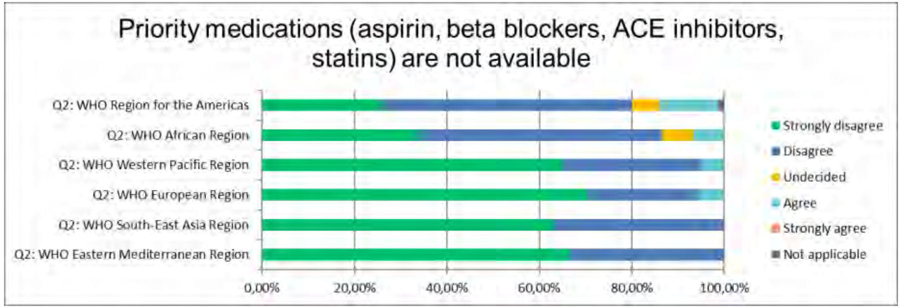

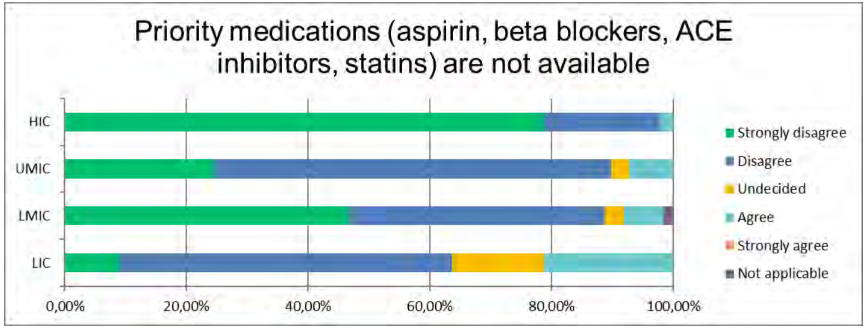

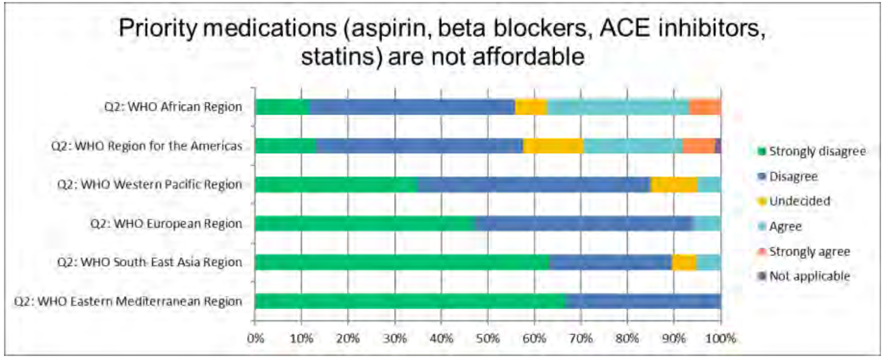

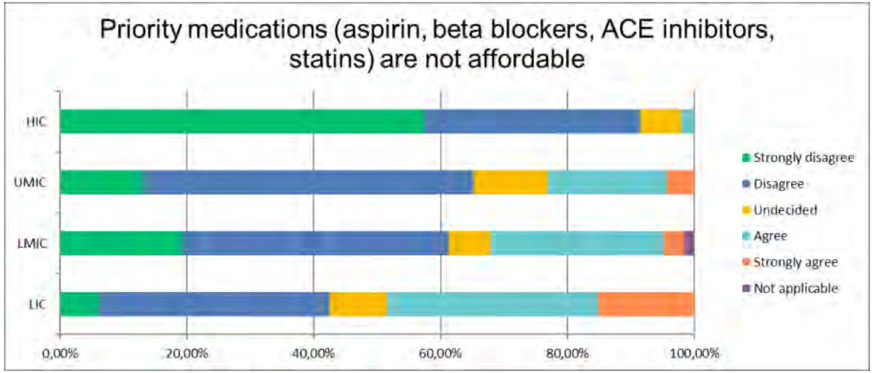

Q18: HEALTH SYSTEM - Please indicate which of these solutions would be relevant in your setting.

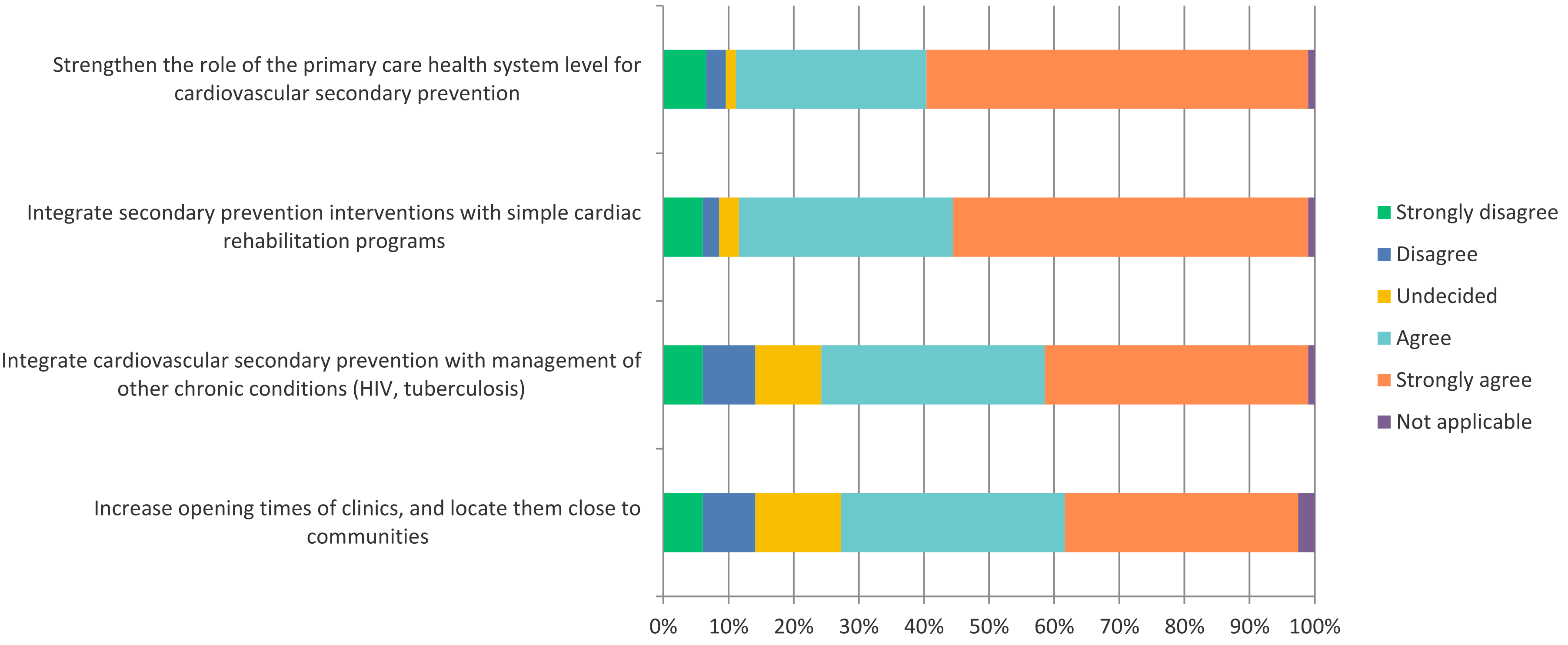

Q19: PATIENTS - Please indicate which of these solutions would be relevant in your setting.

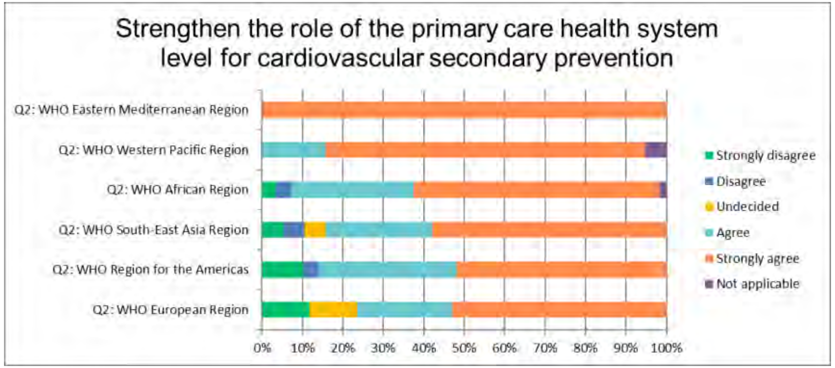

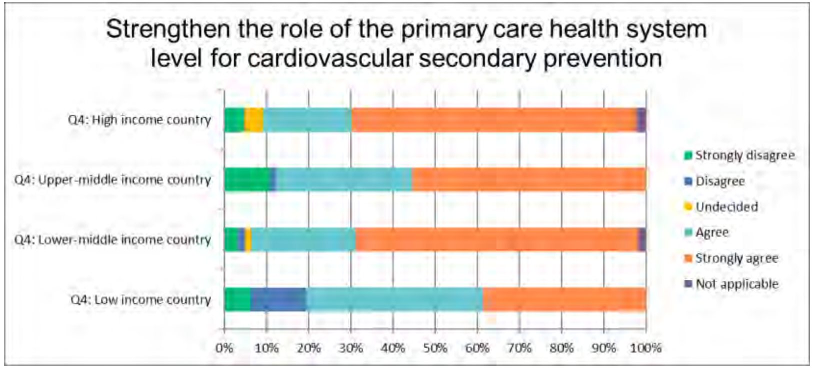

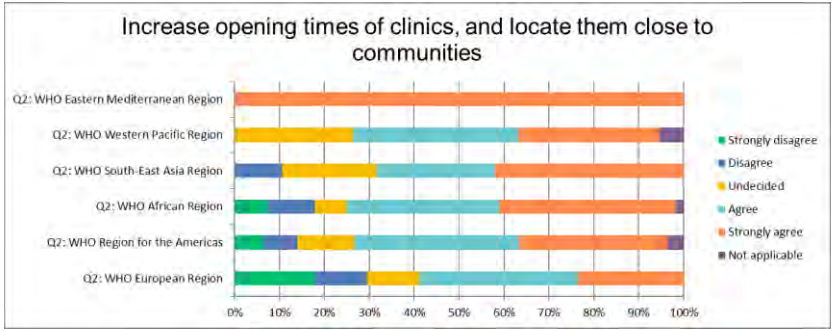

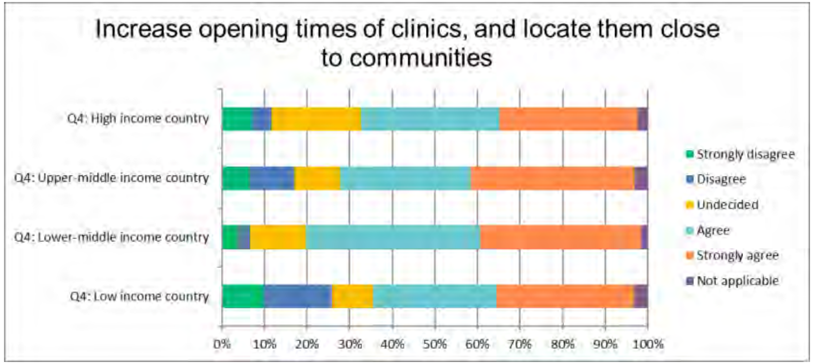

Q18: HEALTH SYSTEM - Please indicate which of these solutions would be relevant in your setting.

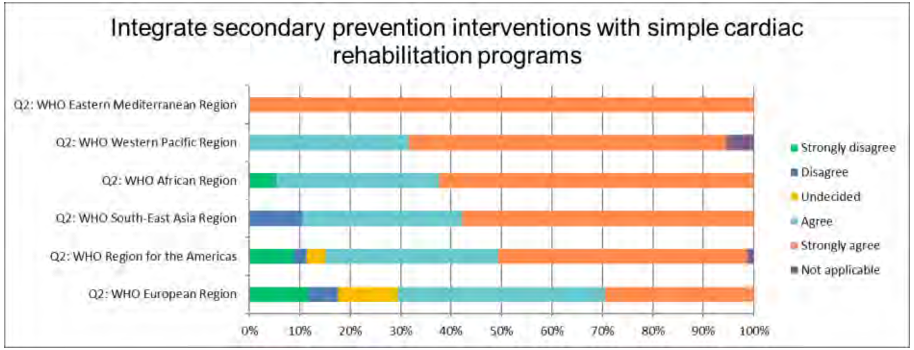

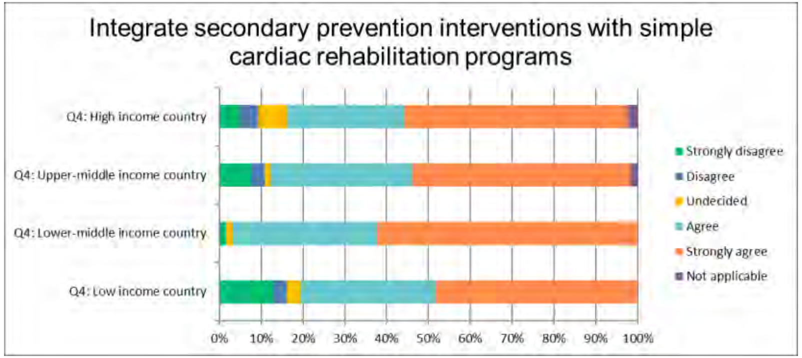

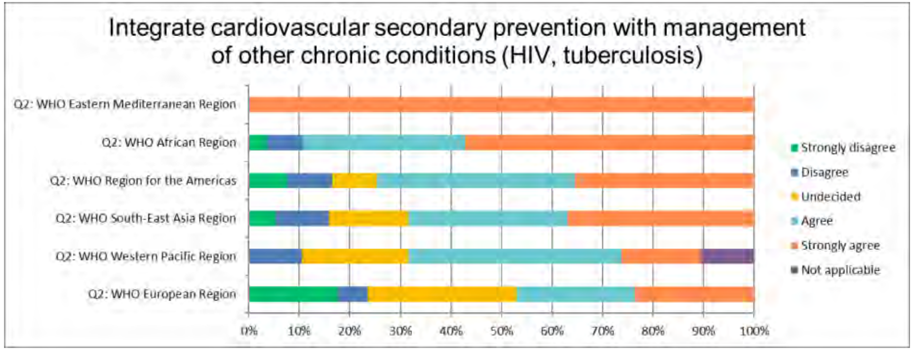

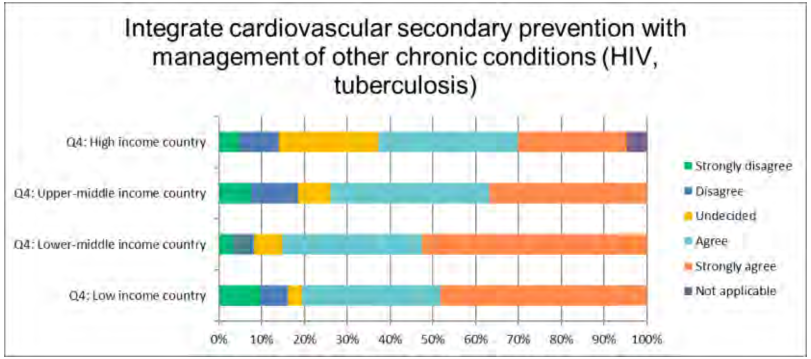

Q20: HCPs - Please indicate which of these solutions would be relevant in your setting.

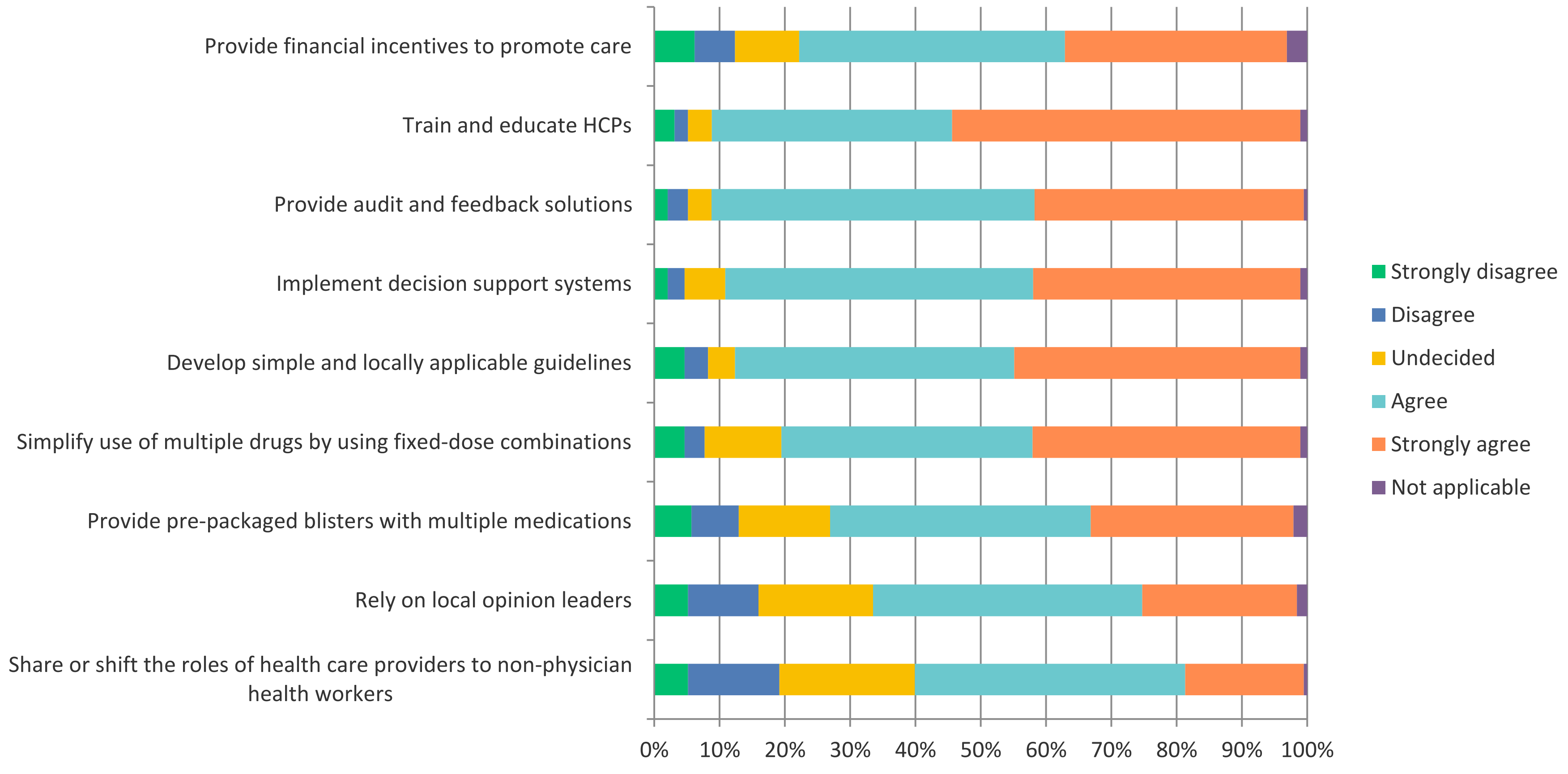

Q20: HCPs - Please indicate which of these solutions would be relevant in your setting.

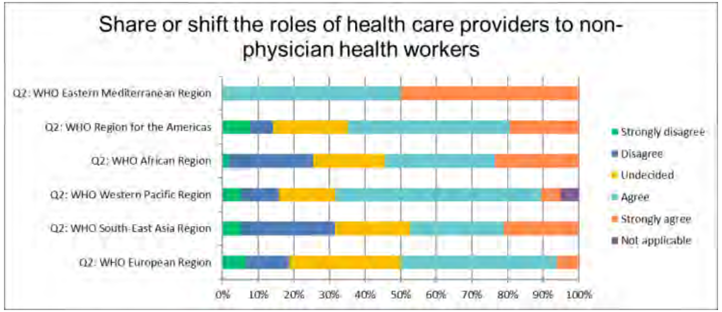

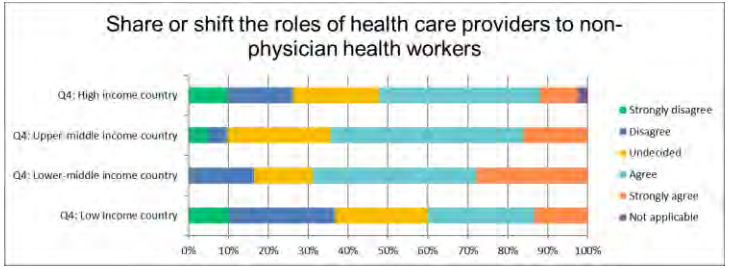

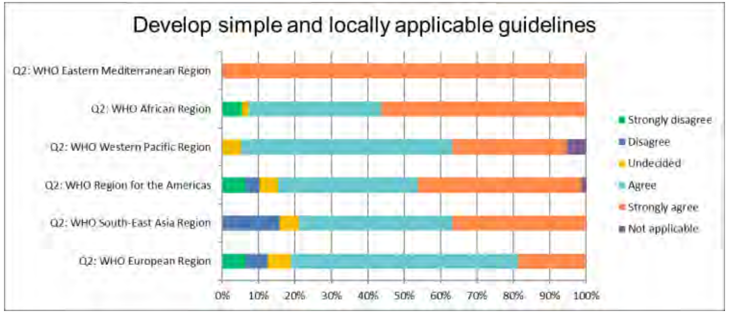

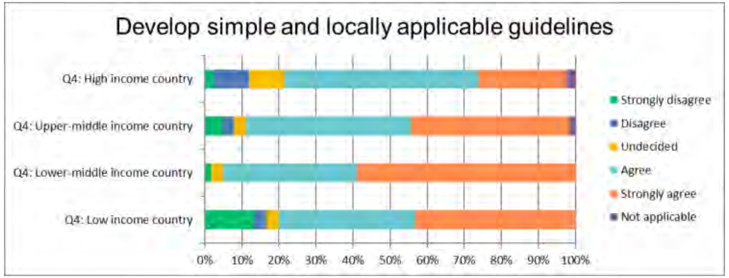

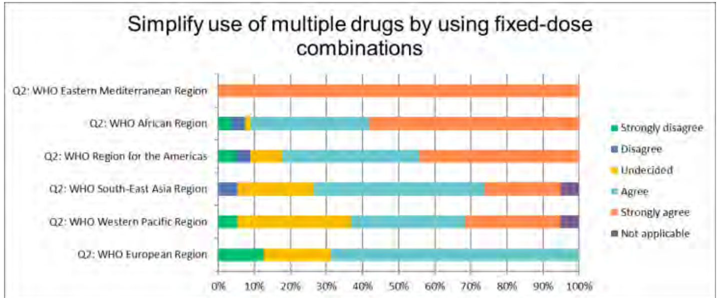

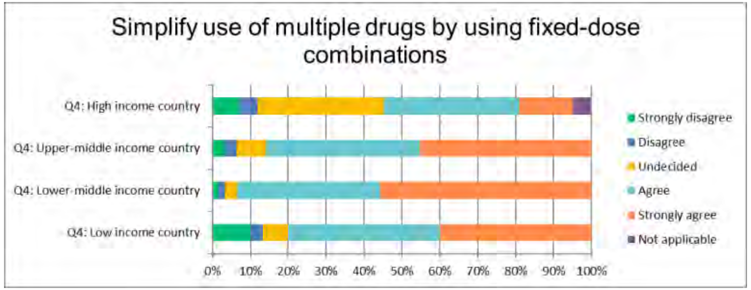

Q20: HCPs - Please indicate which of these solutions would be relevant in your setting.

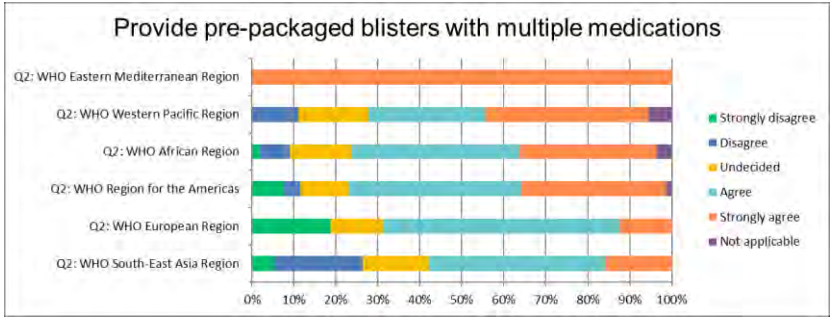

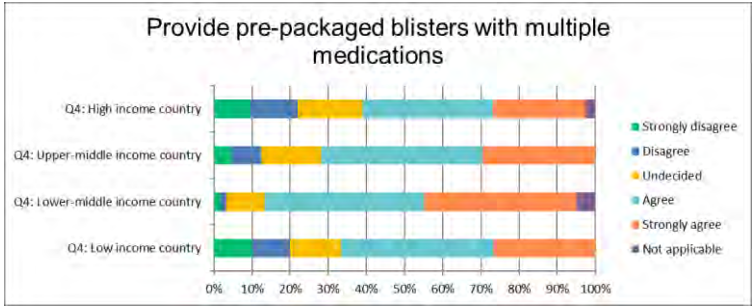

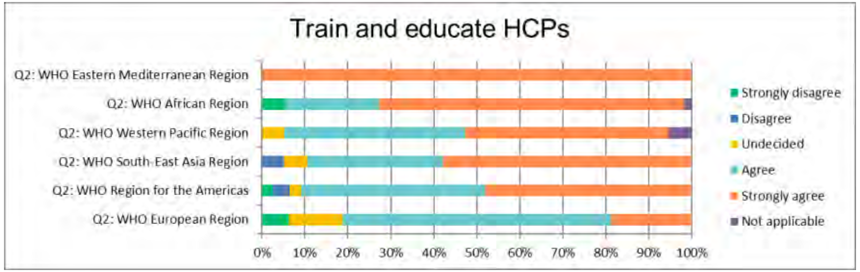

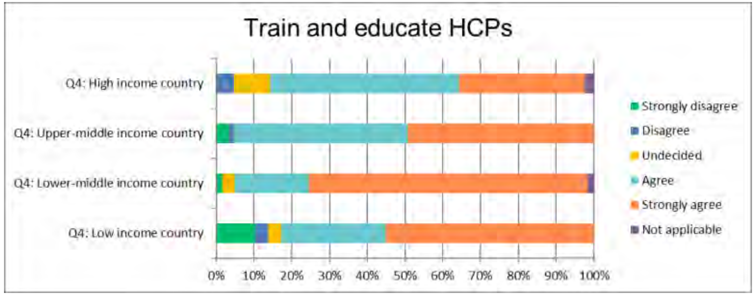

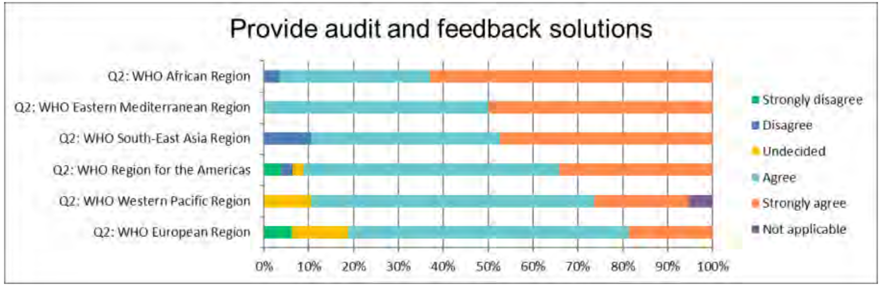

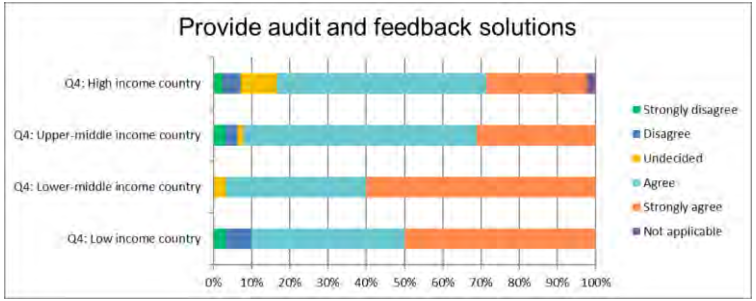

Q20: HCPs - Please indicate which of these solutions would be relevant in your setting.

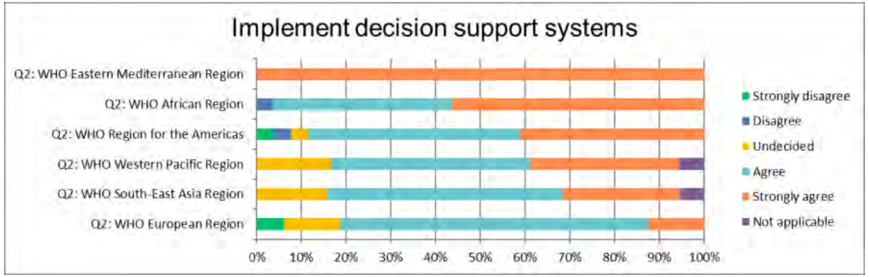

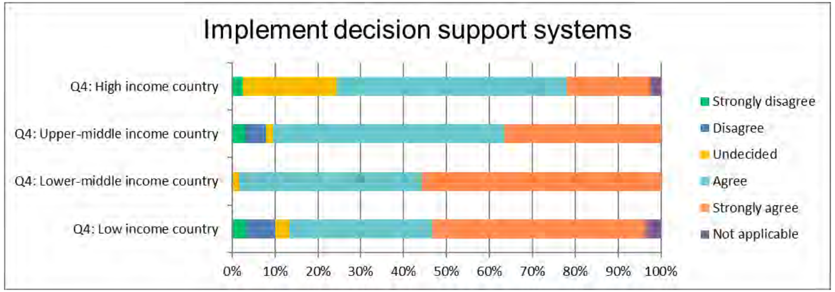

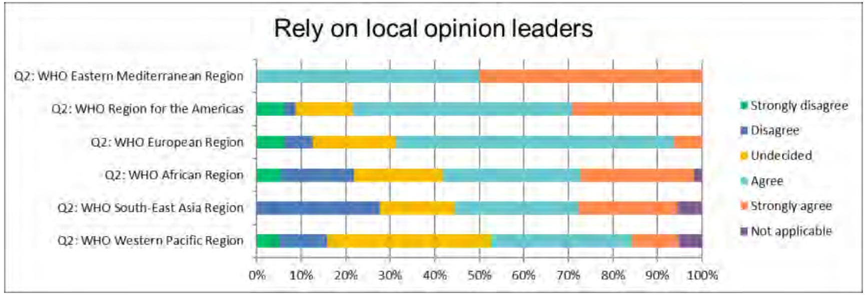

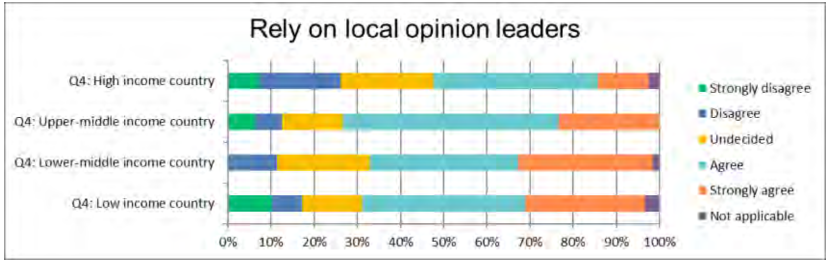

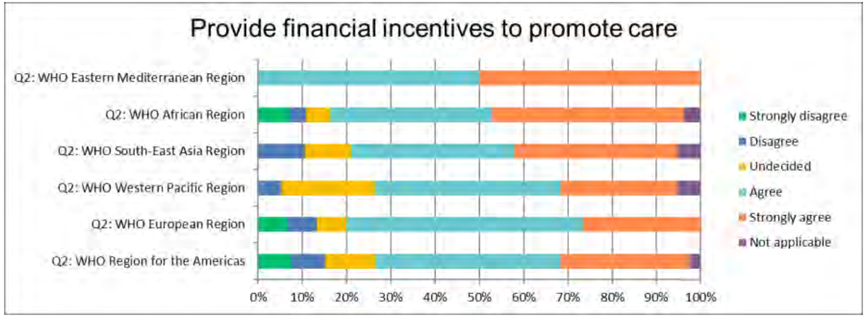

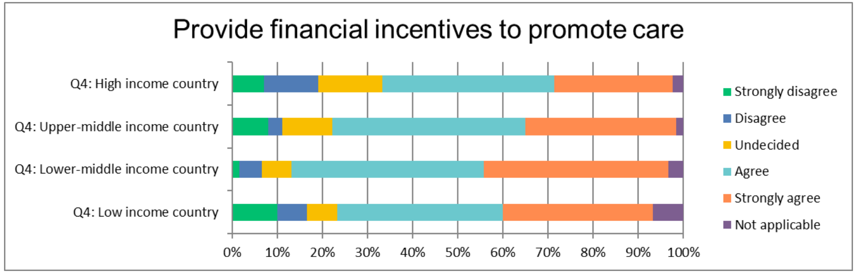

Q21: PRIORITY INTERVENTIONS (lifestyle programmes and medication) - Please indicate which of these solutions would be relevant in your setting.

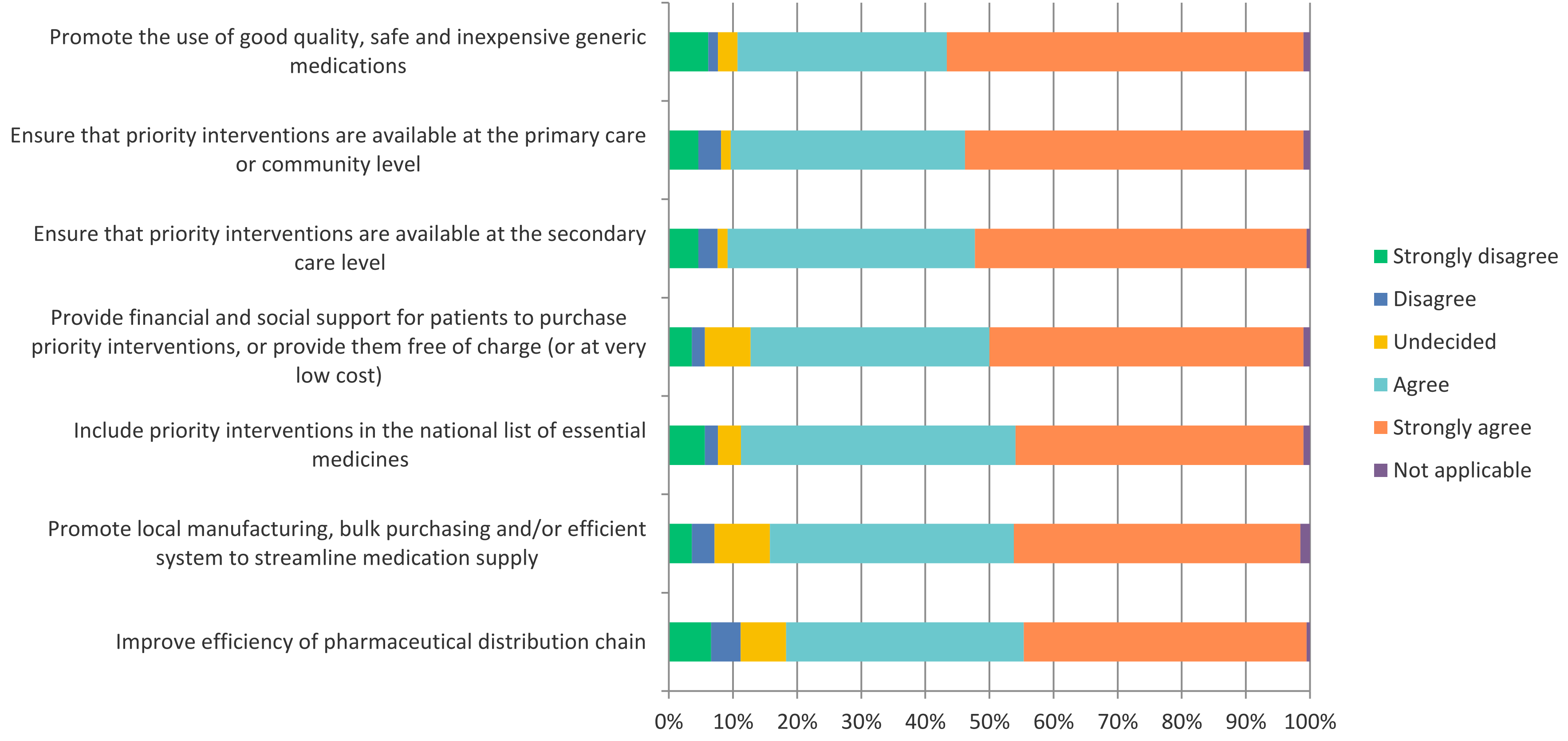

Q21: PRIORITY INTERVENTIONS (lifestyle programmes and medication) - Please indicate which of these solutions would be relevant in your setting.

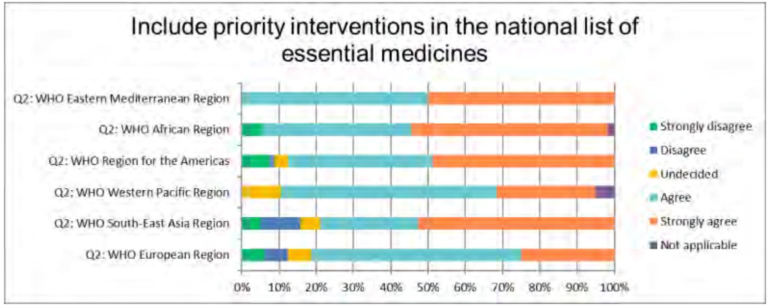

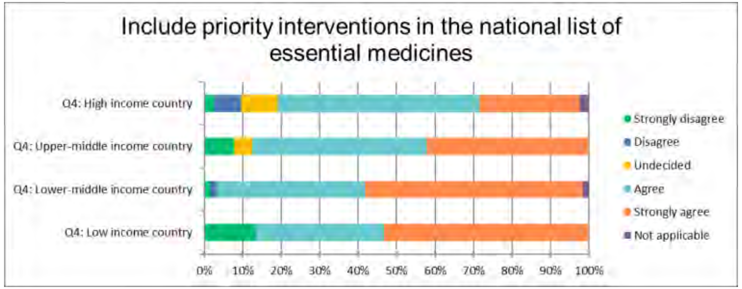

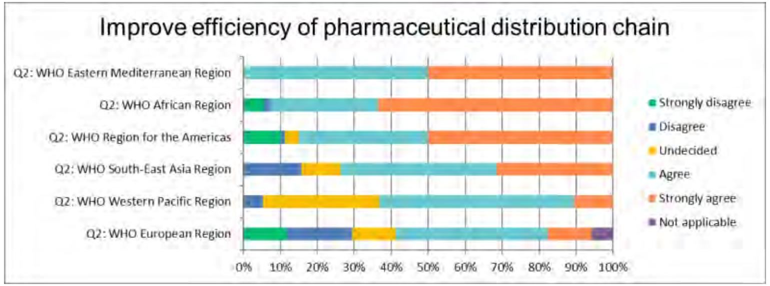

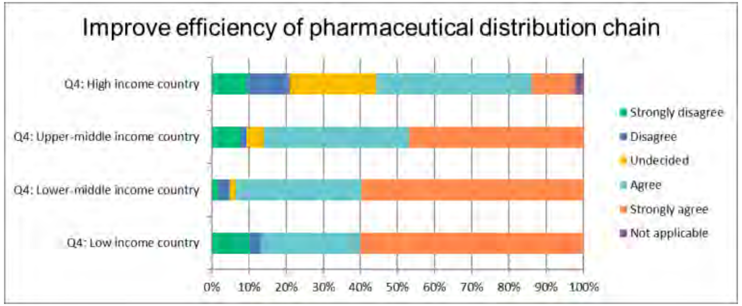

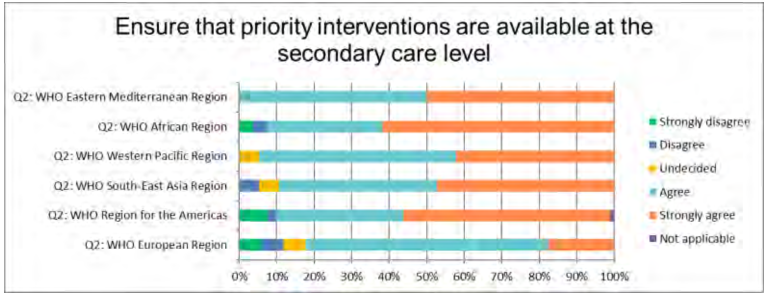

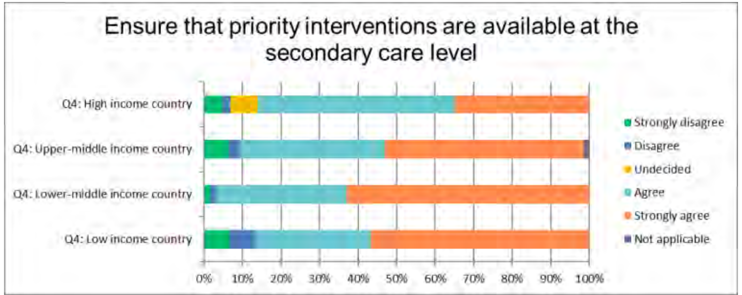

Q21: PRIORITY INTERVENTIONS (lifestyle programmes and medication) - Please indicate which of these solutions would be relevant in your setting.

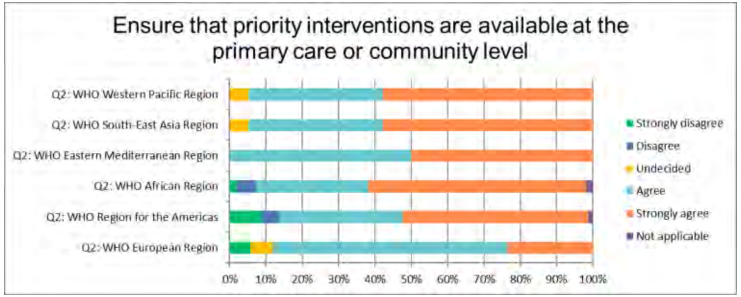

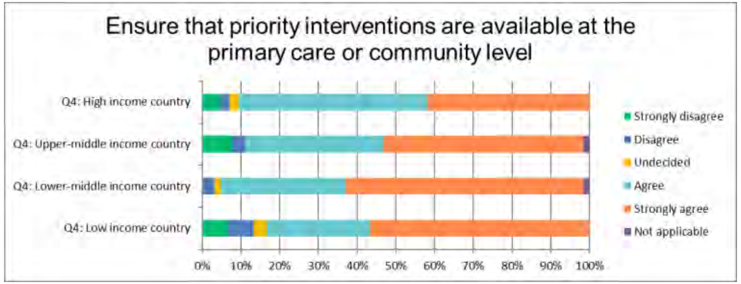

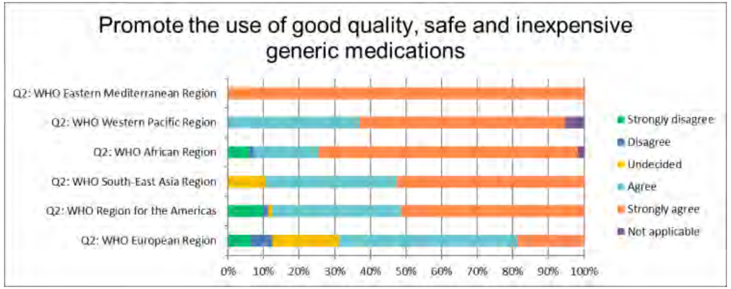

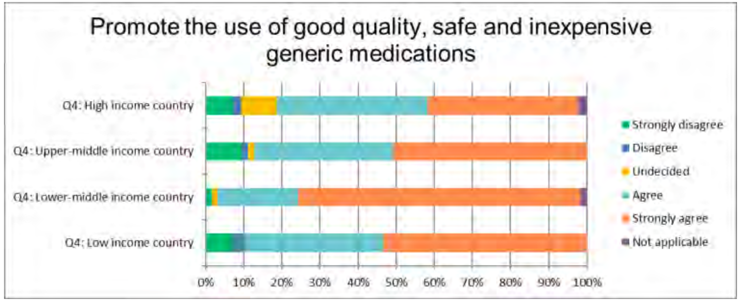

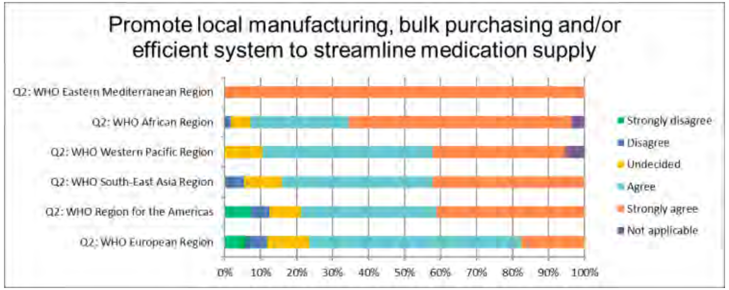

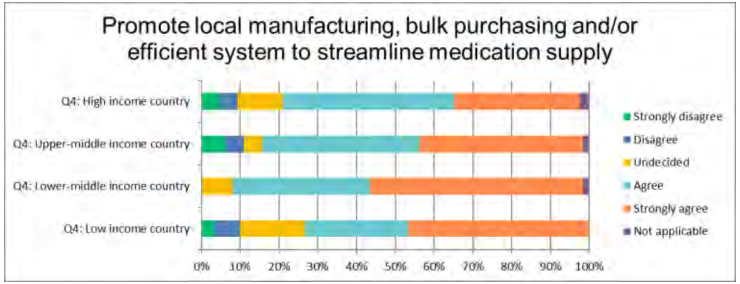

Q21: PRIORITY INTERVENTIONS (lifestyle programmes and medication) - Please indicate which of these solutions would be relevant in your setting.

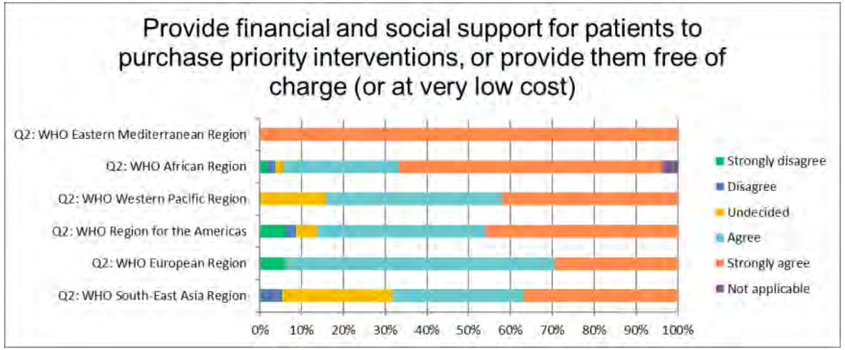

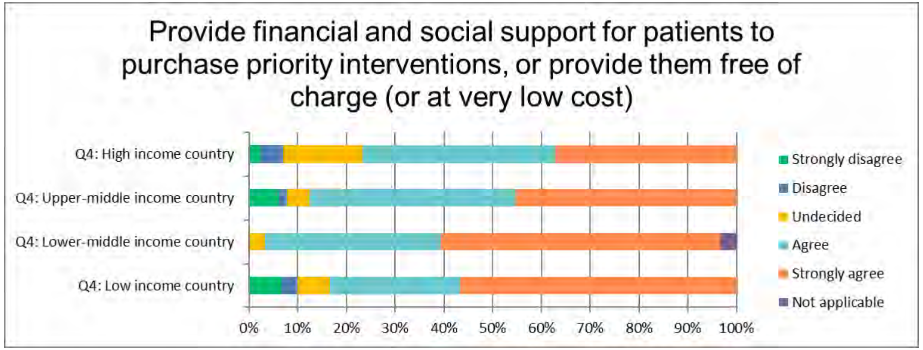

Supplement: Supplementary material. — Supplementary summary of WHF member survey. [file gh-19-1-1278-s1.pdf]
